# An oncogene addiction phosphorylation signature and its derived scores inform tumor responsiveness to targeted therapies

**DOI:** 10.1007/s00018-022-04634-2

**Published:** 2022-12-10

**Authors:** Eleonora Orlando, Matúš Medo, Ariel Bensimon, Aurélie Quintin, Rahel Riedo, Selina M. Roth, Carsten Riether, Thomas M. Marti, Daniel M. Aebersold, Michaela Medová, Ruedi Aebersold, Yitzhak Zimmer

**Affiliations:** 1 Department of Radiation Oncology, Inselspital, Bern University Hospital and University of Bern, Bern, Switzerland; 2 Department for BioMedical Research, Radiation Oncology, University of Bern, MEM-E807, Murtenstrasse 35, 3008 Bern, Switzerland; 3 Department of Biology, Institute of Molecular Systems Biology, ETH Zürich, HPM H25, Otto-Stern-Weg 3, 8093 Zurich, Switzerland; 4 Tumorimmunology, Department for BioMedical Research, University of Bern, Bern, Switzerland; 5 Department of Medical Oncology, Inselspital, University Hospital and University of Bern, 3010 Bern, Switzerland; 6 Thoracic Surgery, Department for BioMedical Research, University of Bern, Bern, Switzerland; 7 Division of General Thoracic Surgery, Inselspital Bern University Hospital, Bern, Switzerland; 8 Faculty of Science, University of Zürich, Zurich, Switzerland

**Keywords:** Oncogene addiction, Protein phosphorylation, Targeted phosphoproteomics, DNA damage response, Patient-derived xenografts, Treatment response, MET, EGFR, ALK, BRAF

## Abstract

**Purpose:**

Oncogene addiction provides important therapeutic opportunities for precision oncology treatment strategies. To date the cellular circuitries associated with driving oncoproteins, which eventually establish the phenotypic manifestation of oncogene addiction, remain largely unexplored. Data suggest the DNA damage response (DDR) as a central signaling network that intersects with pathways associated with deregulated addicting oncoproteins with kinase activity in cancer cells.

**Experimental:**

**Design:**

We employed a targeted mass spectrometry approach to systematically explore alterations in 116 phosphosites related to oncogene signaling and its intersection with the DDR following inhibition of the addicting oncogene alone or in combination with irradiation in MET-, EGFR-, ALK- or BRAF (V600)-positive cancer models. An NSCLC tissue pipeline combining patient-derived xenografts (PDXs) and ex vivo patient organotypic cultures has been established for treatment responsiveness assessment.

**Results:**

We identified an ‘oncogene addiction phosphorylation signature’ (OAPS) consisting of 8 protein phosphorylations (ACLY S455, IF4B S422, IF4G1 S1231, LIMA1 S490, MYCN S62, NCBP1 S22, P3C2A S259 and TERF2 S365) that are significantly suppressed upon targeted oncogene inhibition solely in addicted cell line models and patient tissues. We show that the OAPS is present in patient tissues and the OAPS-derived score strongly correlates with the ex vivo responses to targeted treatments.

**Conclusions:**

We propose a score derived from OAPS as a quantitative measure to evaluate oncogene addiction of cancer cell samples. This work underlines the importance of protein phosphorylation assessment for patient stratification in precision oncology and corresponding identification of tumor subtypes sensitive to inhibition of a particular oncogene.

**Supplementary Information:**

The online version contains supplementary material available at 10.1007/s00018-022-04634-2.

## Introduction

The concept of oncogene addiction was coined for the first time nearly two decades ago [1]. It proposes that despite the vast burden of genetic lesions characterizing cancer cells, given tumors depend on the driving activity of an individual dominant oncoprotein to sustain their growth and survival, thus revealing a promising therapeutic Achilles’ heel of cancer [2]. The observation that the inhibition of driver oncogenic proteins in normal tissues may be pursued with no severe consequences on cellular fitness underlines the distinctive dependency that appears to arise in certain cancer subtypes [3]. Considering the role of receptor tyrosine kinases (RTKs) in promoting cell growth and viability, it is not surprising that their aberrant activation plays imperative roles in the maintenance of the malignant phenotype of specific subtypes of human cancer; this observation underlies the ongoing clinical integration of tyrosine kinase inhibitors (TKIs) [4]. In this respect, successful application of molecularly targeted anticancer strategies requires the identification of molecular markers for accurate patient stratification and identification of susceptible subtypes among patients, which is a major goal of precision medicine [5–8]. Novel strategies involving the identification of multiplex “biomarker panels” are being conceived, which appear to capture more exhaustively the complexity of specific human disorders compared to standard single markers [9].

While accumulated data confirm oncogene addiction both in preclinical as well as in clinical settings, the global molecular mechanisms governing this phenomenon and the apoptotic events following disruption of the oncoprotein activity are not completely elucidated [10]. In this respect, the ‘oncogenic shock’ model proposes that differential attenuation kinetics among pro-apoptotic and pro-survival signals emanating from the driving oncoprotein following its inhibition results in transient signaling imbalance, characterized by rapid diminution of survival effectors whereas proapoptotic ones persist longer, which eventually commits the perturbed cellular system to apoptotic events [11].

Beyond the well-established roles of RTKs in promoting uncontrolled growth and proliferation of cancer cells, findings from recent years indicate a crosstalk between tyrosine kinases that, often when deregulated, act as oncogenes, and the DNA damage response (DDR) machinery. This crosstalk appears to translate in enhanced sensitivity to DNA-damaging agents (DDAs) upon inhibition of the addicting oncoprotein [12–16], thus revealing a novel and specific signaling circuitry potentially regulated by the addicting kinase in cancer cells.

Particularly, the MET receptor tyrosine kinase (RTK) for hepatocyte growth factor (HGF) represents a model recapitulating both the classically associated functions of RTKs in cancer, as well as their involvement in rewiring the DDR machinery. Aberrant MET signaling has been documented in numerous human malignancies [17–20] and MET amplification and overexpression have been associated with poor clinical outcomes [17–25]. Notably, HGF was shown to protect tumor cells from DDAs-induced double-strand breaks (DSBs) in a phosphatidylinositol 3-kinase/RAC-alpha/serine/threonine-protein kinase (PI3K/AKT)-dependent manner [26, 27]. In line with these data, accumulating evidence suggests that MET inhibition results in radiosensitization in MET-addicted systems [28–30]. In the clinic, MET expression was found to inversely correlate with complete remission of primary tumors of the oropharynx following radiation therapy [31].

In the past years, proteomics techniques have been adopted as methods of choice to investigate dynamic processes involving phosphorylation-related signaling networks in various contexts [32]. Specifically, mass spectrometry (MS)-based phosphoproteomics emerged as a valuable tool capable of capturing cellular phosphorylation events in an unbiased, quantitative and highly sensitive manner [33, 34]. In the context of RTKs, pioneering phosphoproteomics studies unravelling their complex biology focused primarily on EGFR [35]. Phosphoproteomes have also been studied to discover signaling networks in DSBs-induced DDR [36, 37] and to monitor treatment responses and outcomes [38–40]. We reported in a recent MS-based phosphoproteomic study that MET inhibition modulates the cellular response to IR by regulation of multiple key DDR-related phosphorylations [41].

Here we employed the targeted phosphoproteomic technique selected reaction monitoring (SRM), to assess changes in 116 protein phosphorylations at the RTK-DDR intersection following targeted oncogene inhibition as a single treatment and combined with radiation-induced DNA damage. We report a composite phosphorylation signature associated with addiction to four distinct oncogenes, i.e., MET, EGFR, ALK and BRAF, which can be detected also in ex vivo-treated NSCLC patient samples. A score derived from this oncogene addiction phosphorylation signature strongly correlates with phenotypic responses of cancer models towards targeted therapy and can be translated as a base for personalized therapy selection.

## Material and methods

### Cell lines

The human gastric carcinoma cell lines GTL-16 (RRID:CVCL_7668), SNU-638 (RRID:CVCL_0102) and Hs746T (RRID:CVCL_0333) were kindly provided by Dr. Paolo Comoglio (Medical School University of Torino, Italy), Korean Cell Line Bank, Dr. Morag Park (Cancer Research Centre, McGill University, Montreal, Canada) and Dr. Silvia Giordano (Medical School University of Torino, Italy), respectively. The human lung carcinoma cell line EBC-1 (RRID:CVCL_2891) was obtained from Dr. Silvia Giordano (Medical School University of Torino, Italy) and the human lung carcinoma cell lines H1993 (RRID:CVCL_1512), HCC827 (RRID:CVCL_2063) and H1648 (RRID:CVCL_1482) were kindly provided by Dr. Sunny Zachariah (UT Southwestern Medical Center, Dallas, TX). The human lung carcinoma cellular models H1975 (RRID:CVCL_1511) and PC-9 were kindly provided by Dr. Alexandre Arcaro (Pediatric Hematology/Oncology, University of Bern, Switzerland), and the human melanoma cellular model G361 (RRID:CVCL_1220) was obtained from American Type Culture Collection, CRL-1424. The human lung carcinoma cell line A549 (RRID:CVCL_0023) was provided by Dr. Marco Alves (Institute of Immunology and Virology, University of Bern, Switzerland), the human pharyngeal carcinoma cell line Detroit-562 from CLS, Cell Line Services GmbH (Eppelheim, Germany), and the human thyroid carcinoma model KAT-4 was kindly provided by Dr. Kenneth Ain (Chandler Medical Center, University of Kentucky, KY).

A549 and Hs746T cells were cultured in DMEM (GIBCO, Invitrogen) supplemented with 10% foetal calf serum (FCS) (Sigma) and antibiotic–antimycotic (penicillin 100 U/ml, streptomycin sulfate 100 U/ml, amphotericin B as Fungizone 0.25 µg/ml; GIBCO). Detroit-562 were maintained in MEM medium (GIBCO, Invitrogen) supplemented with 10% FCS (Sigma), non-essential amino acid solution (NEAA) (Sigma) and antibiotic–antimycotic. G361 cells were grown in McCoy’s 5A Modified Medium (Invitrogen) supplemented with 10% foetal calf serum (FCS) and antibiotics. All the other cell lines were cultured in RPMI medium (GIBCO, Invitrogen) supplemented with 5–10% FCS (Sigma) and antibiotic–antimycotic.

### Inhibitors

The small molecule inhibitors tepotinib (EMD1214063; 3-(1-(3-(5-(1-methylpiperidin-4-ylmethoxy)-pyrimidin-2-yl)-benzyl)-1,6-dihydro-6-oxo-pyridazin-3-yl)-benzonitrile) (Merck KGaA, Darmstadt, Germany), gefitinib (Selleckchem), AZD9291 (Selleckchem), crizotinib (Selleckchem) and vemurafenib (Selleckchem) were dissolved in dimethyl sulfoxide (DMSO) and kept at − 20 °C. Working solutions were prepared freshly in the corresponding media at indicated concentrations.

Inhibitors were added to cells 24 h before irradiation at the following concentrations unless otherwise specified: tepotinib 50 nM, gefitinib 100 nM, AZD9291 100 nM, crizotinib 100 nM, vemurafenib 100 nM.

### Delivery of irradiation

Cells and organotypic models were irradiated using a ^137^Cs source (Gammacell 40, MDS Nordion, Ottawa, ON, Canada) at a dose rate of 0.86 Gy/min with a single dose of 10 Gy unless otherwise specified.

### Antibodies

Monoclonal antibodies used in this study were directed against phospho-MET (Tyr1234/1235) (Cell Signaling Technology), MET (clone D1C2, Cell Signaling Technology), phospho-EGFR (Tyr845) (Cell Signaling Technology), phospho-ALK (Tyr1064) (Cell Signaling Technology), phospho-ATM (Ser1981) (Cell Signaling Technology), phospho-c-Raf (Ser259) (Cell Signaling Technology), phospho-AKT (Ser473) (Cell Signaling Technology), phospho-MAPK (Thr202/Tyr204) (Cell Signaling Technology), phospho-H2AX (Ser139) (Upstate), Ki67 (Cell Signaling Technology) and ß-actin (Millipore Chemicon).

### Protein extraction and Western blot analysis

Cell lysis was performed in urea lysis buffer (20 mmol/l HEPES, 9.0 mol/l urea, 1 mmol/l sodium orthovanadate, 2.5 mmol/l sodium pyrophosphate, 1 mmol/l β-glycerol-phosphate) followed by sonication. Lysis of tissues was performed by mechanical disruption using TissueLyser (QIAGEN) in urea lysis buffer. The BioRad protein quantification reagent (Bio-Rad Laboratories, Inc., RRID:SCR_008426) was used to determine protein concentrations. A volume corresponding to 50 µg of total proteins was resolved by SDS-PAGE. Separated proteins were then transferred to PVDF membranes. Horseradish peroxidase-conjugated secondary antibodies were detected by an ECL kit (AmershamPharmacia Biotech, Little Chalfont, UK).

### Cell viability

Cells were plated onto 6-well plates (15,000 cells/plate) and pretreated with the indicated specific compound or DMSO 24 h prior irradiation delivery. After irradiation, cells were incubated for 7 days, subsequently fixed and stained by 2% crystal violet dissolved in methanol (1:3 v/v) and acetic acid (2:3 v/v). The assessment of the number of viable cells was performed using ImageJ software (imagej.nih.gov/ij/: RRID:SCR_003070). Statistical analysis and graphical presentation of the data were performed using Prism Graph (version 5.03). Particularly, data for each treatment group were compared to evaluate significance using the Student *t*-test. Differences with *P* values < 0.05 were considered statistically significant (*P < 0.05; **P < 0.01; ***P < 0.001, ****P < 0.0001).

### Animals

NOD/LtSz-scid IL2R null (NSG) mice have been previously described [42]. Mice were housed under specific pathogen-free conditions in individually ventilated cages with food and water ad libitum and were regularly monitored for pathogens. Animal experiments were approved by the local experimental animal committee of the Canton of Bern and performed according to Swiss laws for animal protection.

### Patient-derived xenografts

Fresh lung specimens were obtained from lung cancer patients at the University Hospital of Bern, Switzerland (inclusion criteria were diagnosed NSCLC and tissue availability). All patients gave informed written consent for the use of the surgical material for research purposes, which was approved by the Ethics Committee of the Canton of Bern (KEK-BE:2018-01801). Non-necrotic tissue was cut into small fragments (100 mm^3^) to maintain the tissue integrity. Tumor fragments were then transplanted s.c. into the flanks of recipient female NSG mice of 6–8 weeks of age (animal license number BE 76/17). Tumor growth was monitored by measuring the tumor dimensions with a caliper at different time points after transplantation. Tumor volume was calculated according to the formula *V* = *π* × *abc*/6, where *a*, *b*, and *c* are orthogonal diameters. Once tumors reached a size of 1 cm^3^, animals were sacrificed, and tumors were explanted for further characterization and establishment of organotypic models.

### Organotypic models

Upon tumor extraction, organotypic tissue cultures (OTCs) were generated by a Vibratome VT1200 (Leica Microsystems) as previously reported [43]. 300 μm-thick OTCs were maintained in CutPrime medium supplemented with antibiotic–antimycotic (penicillin 100 U/ml, streptomycin sulfate 100 U/ml, amphotericin B as Fungizone 0.25 µg/ml; GIBCO) in a humidified 5% CO_2_ atmosphere at 37 °C. OTCs were kept in culture for one day before treatment with either vehicle (DMSO), tepotinib (50 nM), gefitinib (100 nM) or crizotinib (500 nM), corresponding to 24 h before delivery of irradiation (10 Gy) in case of a combined treatment. Altogether, OTCs for SRM-based phosphoproteome analysis were kept in culture for 48 h, whereas the assessment of Ki-67 positive nuclei was performed upon keeping OTCs in culture for 72 h, corresponding to 48 h upon treatment. Fresh medium containing drugs was replaced daily.

### Immunohistochemistry

OTCs were fixed in 10% formalin overnight at 4 °C. Sections were deparaffinized and rehydrated and antigen retrieval was performed in Tris–EDTA buffer (pH 9). After blocking of endogenous peroxidase and blocking of the sections in goat serum, primary antibodies for Ki67 and phosphorylated MET, EGFR and ALK were applied and detected with the Vectastain ABC Kit (Vector Laboratories) and 3,3′-Diaminobenzidine (DAB) (Sigma-Aldrich), according to the manufacturer’s instructions. Slides were counterstained with haematoxylin. Images were obtained with a Pannoramic 250 Flash III Scanner (3D Histech) at 100 × magnification. The percentage of Ki67-positive nuclei was assessed by three independent observers blinded to each other’s results.

### Selected reaction monitoring mass spectrometry

#### Selection of targeted phosphopeptides

The pool of 61 candidate SRM targets previously investigated by Bensimon et al. [41] was expanded with 55 additional phosphopeptides previously associated with DNA damage response and/or receptor tyrosine kinase signaling. In detail, the selection was based on discovery shotgun phosphoproteomics experiments of the EBC-1 cell line treated with METi, IR and their combination (PTMScan™, Cell Signaling Technology; unpublished data). First, the following two criteria have been applied for phosphopeptide selection: (1) at least 2.5-fold change in at least one of the two conditions METi or METi + IR as compared to control, and (2) GO terms “DNA repair", "DNA damage checkpoint", "double-strand break repair", "chromatin remodeling", "cellular response to DNA damage stimulus", "regulation of signal transduction by p53 class mediator", "cell cycle", apoptotic process", "cell proliferation", "signal transduction", "DNA replication", "cell migration", "cell–cell adhesion", "gene expression", or presence of the ATM/ATR substrate phosphorylation motif. Afterwards, technical constraints to the resulting list of peptides were applied (peptides shorter than 25 aa, with less than three phosphorylated sites, detected in previously published MS datasets) and heavy peptides were tested in a shotgun mode for quality and light/endogenous for detectability. The full list of 116 phosphopeptides selected for measurement is provided in Table S1. For each targeted phosphopeptide, mass spectrometric assays were generated to selectively detect and quantify these phosphorylations (Table S2). A total of 116 phosphopeptides were measured across the various perturbations in three independent replicates.

#### Sample preparation for MS analysis

Cells were washed, scraped in ice-cold PBS and spun down for 5 min at 1000 rpm, and resulting pellets were stored at − 80 °C until further processing. Cell pellets were resuspended in 8 M urea solution containing 0.1 M ammonium bicarbonate (ABC) and disrupted by sonicating for 10 min. Resulting extracts were spun for 10 min at 1200 rpm and protein concentration was determined (BCA Protein Assay (Thermo Scientific, Rockford IL, USA)).

Lysis of OTCs was performed by pressure cycling technology (PCT) as previously described [44]. Each tissue slice was placed in a microTube (Pressure BioSciences, Inc., South Easton, MA, USA) with 150 μl lysis buffer containing 8 M urea, 0.1 M ABC, protease inhibitor cocktail (Roche, Switzerland) and PhosSTOP phosphatase inhibitor cocktail (Roche, Switzerland). Tissue lysis was performed by a barocycler NEP2320-45 k using 60 cycles, each one consisting of 50 s at 45 000 p.s.i. high pressure alternated by 10 s of atmospheric pressure at 33 °C.

Disulfide bonds were reduced with 5 mM tris(2-carboxyethyl)phosphine at 37 °C for 30 min and free thiols were alkylated with iodoacetamide at a final concentration of 10 mM at room temperature for 30 min in the dark. The solution was subsequently diluted with 0.1 M ABC to a final concentration of 1.5 M urea and digested overnight at 37 °C with sequencing-grade modified trypsin (Promega, Madison WI, USA) at a protein-to-enzyme ratio of 50:1. The digestion was halted by acidification with trifluoroacetic acid (Thermo Scientific) to a final pH < 3. Peptides were desalted on a C18 Sep-Pak cartridge (Waters, Ireland) and dried under vacuum. Phosphopeptides were isolated from 1 mg of total peptide mass with TiO_2_ using a modified protocol from [45, 46].

#### Data acquisition

The solution used to resuspend peptides from MS analysis was added with a mix containing heavy-labelled synthetic reference peptides (Thermo Scientific). Development and validation of SRM assays were performed using unpurified synthetic versions of the target peptides labelled with heavy isotopes at the C-terminal Lys (+ 8 Da) or Arg (+ 10 Da) [47]. To extract their SRM coordinates, we first performed shotgun analysis on synthetic peptide mixtures. For each target peptide, the ten most intense SRM transitions were selected using Skyline [48]. For assay refinement, the five most intense transitions for each peptide were selected and the peptide retention time for scheduled SRM acquisition was annotated.

Light and the corresponding heavy transitions were measured by scheduled SRM to assess co-elution of endogenous (light) peptides and their spiked-in (heavy) surrogates.

Samples were measured on a triple quadrupole/ion trap mass spectrometer (5500 QTrap, ABSciex) connected with a nanoelectrospray ion source. Chromatographic separation of peptides was achieved with an Eksigent Nano LC system (Eksigent Technologies, SCIEX) connected to a 15-cm fused-silica emitter with 75-µm inner diameter (MSwil, Switzerland) packed in-house with a Magic C18 AQ 3-µm resin (WICOM International GmbH, Switzerland). Peptide mixtures were loaded on the column and analyzed by LC–MS/MS upon separation with a linear gradient of acetonitrile/water from 2 to 40% acetonitrile in 55 min at a flow rate of 300 nl/min. Scheduled SRM mode was applied at a unit resolution (0.7 *m*/*z* half-maximum peak width) for both Q1 and Q3 analyzers. Collision energy (CE) was calculated according to the following formula: CE = 0.044 × *m*/*z* precursor + 5.5 for doubly charged ions and CE = 0.051 × *m*/*z* precursor + 0.55 for triply charged ions [49]. SRM peaks were manually inspected with Skyline [48].

#### Data analysis and deposition

For targeted data analysis, Skyline [48] was employed. Internal standards were used for confident peptide identification by comparison of fragmentation and chromatographic properties with synthetic peptides (rdotp > 0.9). Peptides’ peak integration was verified manually. Statistical analysis was performed using MSstats [50]. The MSstats output data are available as Supplementary data 1–3 and the mass spectrometry proteomics data have been deposited to the ProteomeXchange Consortium via the PRIDE [51] partner repository with the dataset identifier PXD037406.

#### Functional characterization of selected phosphopeptides

The functional enrichment of the 104 unique SRM-analyzed phosphopeptides was obtained using the String database (https://string-db.org, version 11.0; RRID:SCR_005223), yielding a list of 899 enriched biological processes (false discovery rate below 0.05) characterized by their GO terms, descriptions, phosphopeptide counts, and phosphopeptide set sizes (i.e., the total numbers of phosphopeptides participating in each process) (Supplementary data 4). We used the list of enriched biological processes to categorize the input phosphopeptides into the following four distinct groups: cell cycle (processes with "cell cycle" in their names), DNA damage (processes with "DNA damage" or "DNA repair" in their names), kinase activity (processes with "kinase activity" or "kinase signaling" in their names), and apoptosis (processes with "cell death" or "apoptotic process" in their names); the selected processes are summarized in Supplementary data 5. To allow for a unique attribution, we assigned each phosphopeptide to the group with the most specific relevant GO term. The resulting group sizes are 27, 19, 19, and 15 for "cell cycle", "kinase activity", "apoptosis", and "DNA damage", respectively. The remaining 24 SRM-analyzed phosphopeptides are not assigned to any of the four processes. The group assignment is displayed in Supplementary data 5. Figure S1 illustrates the SRM-analyzed phosphopeptides with their interaction strengths obtained using the String database; phosphopeptides whose interaction strength is 0.8 or more, are connected with links.

Upstream kinases of the OAPS phosphorylation sites were assigned using the PhosphoSitePlus database (PhosphositePlus, v6.6.0.4), including “Kinase, in vitro” and/or ‘Putative in vivo kinase” evidence.

### Oncogene addiction phosphorylation signature (OAPS)-derived scores

To quantify a sample’s response to the inhibition of a particular oncogene, we developed a score directly based on the identified protein phosphorylation signature. The score is defined as the negative log-fold change upon the oncogene inhibition (estimated by MSstats) averaged over all eight signature protein phosphorylations (ACLY S455, IF4B S422, IF4G1 S1231, LIMA1 S490, MYCN S62, NCBP1 S22, P3C2A S259 and TERF2 S365; see main text for details on the signature construction). In particular,

$$\mathrm{OAPS}=-\frac{1}{8}\sum_{p=1}^{8}\mathrm{LFC}\left(p\right),$$

where *p* is used to enumerate the eight phosporylations listed above and LFC is the estimated log-fold change upon treatment. For example, if the log-fold change for each phosphorylation is -1, the score is + 1.

## Results

### MET-positive cellular models differ in their responses to METi alone and in combination with IR

We have recently reported that in specific MET-overexpressing cell models, MET inhibition (METi) largely affects numerous phosphorylation circuits in cancer cells and regulates also cellular response to ionizing radiation (IR) by modulating multiple key effectors of the DDR machinery [41]. To investigate whether this phenomenon could relate to an addicted state of these cells to the MET RTK and thus serve as a lead for selection of METi-responsive tumors in clinical settings, we aimed at detailed analysis of METi- and/or IR-induced phosphorylation signatures in nine distinct cellular models that commonly feature constitutively active MET receptor and are thus plausible candidates for METi-targeted therapies: the non-small cell lung cancer (NSCLC) lines EBC-1, H1993, H1648, and A549, the gastric carcinoma models GTL-16, SNU638, Hs746T, the thyroid carcinoma cell line KAT-4 and the pharyngeal carcinoma cells Detroit 562.

**Fig. 1:**
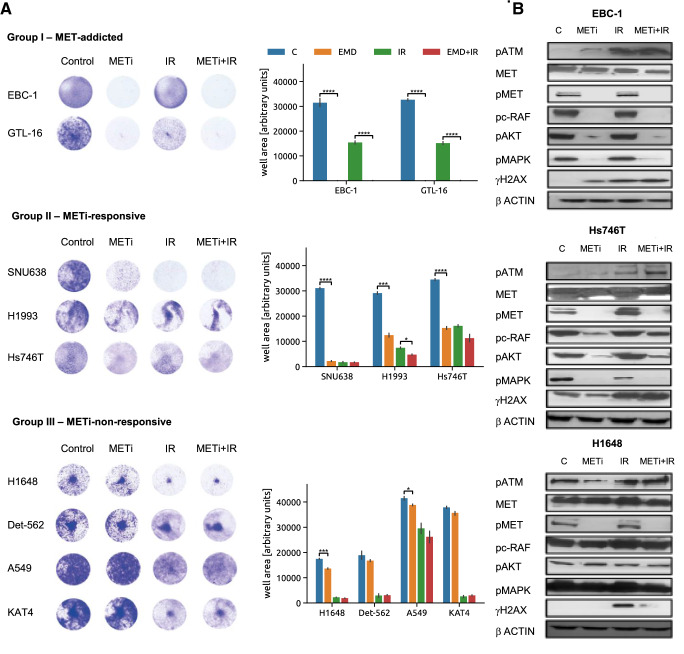
Phenotypic responses of MET-positive cellular models. **A** Representative pictures (left) and quantification (right) of viability assays to assess sensitivity of MET-positive cellular models to METi exerted by the MET inhibitor tepotinib, IR and the combination treatment. Statistical analysis was performed by GraphPad Prism. p values were calculated by Student *t*-test (*P < 0.05; **P < 0.01; ***P < 0.001, ****P < 0.0001). **B** Western Blot analysis of MET total levels and phosphorylation (pMET (MET Y1234/5), phosphorylation status of MET downstream effectors (pc-Raf (c-Raf S259)), pAKT (AKT S473), and pMAPK (MAPK T202/204)) as well as DDR players (pATM (ATM S1981), pH2AX (H2AX S139)) in EBC-1, Hs746T and H1648 cell lines indicative for the three groups of cellular models. β-actin was used as loading control

To classify these models based on their MET signaling dependency, we first assessed their phenotypic responses to METi, IR and the METi + IR combined treatment by a cell viability assay and divided them into three tentative groups sharing similar responses (Fig. 1A). EBC-1 and GTL-16 cells that were previously reported to be particularly sensitive to METi and to modulate their DDR signaling upon METi showed a complete loss in cell viability upon METi alone as well as upon the combined perturbation; we have thus classified them as representative for MET-addicted tumors (Group I). A second group, including the H1993, Hs746T and SNU638 cells, was termed ‘METi-responsive’ (Group II). The viability of these cells has also been significantly reduced upon METi but only minimal viability changes have been observed upon the combined treatment as compared to IR alone (Fig. 1A). Finally, H1648, A549, Detroit 562 and KAT4 did not show any or only marginal reduction in cell viability upon METi alone or in combination with IR (Fig. 1A) despite reduction in phospho-MET (Tyr1234/5; pMET) levels upon METi (Fig. 1B), thus representing ‘METi-unresponsive’ systems (Group III).

Although MET phosphorylation was inhibited by METi in cell lines representative of each of the groups, the phosphorylation status of MET downstream effectors (i.e., pAKT, pcRAF and pMAPK) as well as major markers of DDR activation (i.e., pATM and H2AX) upon METi alone and with IR was modulated differentially (Fig. 1B). MET-addicted EBC-1 cells displayed reduction in the phosphorylation status of MET downstream effectors upon METi. This was accompanied by increased phosphorylation of the two key DDR players ATM and H2AX that was even further enhanced when combined with IR. METi-responsive Hs746T cells displayed reduction in phosphorylated MET downstream effectors upon METi, but neither concomitant increase in phosphorylated ATM and H2AX, nor synergistic regulation of these phosphorylations upon combination with IR was observed (Fig. 1B). Finally, the METi-unresponsive H1648 cells did not show any reduction in phosphorylation of MET downstream effectors or any significant changes in pATM and pH2AX levels upon METi even though the levels of pMET were reduced (Fig. 1B). Alike to the METi-responsive models, no synergistic regulation of these two DDR-related phosphorylations upon H1648 exposure to METi and IR could be observed (Fig. 1B). As the impact of METi on key signalling nodes involved in cell survival, proliferation and response to DNA damage may represent a hallmark of response of MET-addicted models, we further explored the differences between the three groups of MET-expressing cell lines by targeted phosphoproteomics.

### Identification of a ‘MET addiction phosphorylation signature’ that precedes onset of apoptotic markers upon METi

To reveal METi-mediated phosphorylations regulated by METi and/or IR solely in the context of a MET-addicted phenotype, selected reaction monitoring (SRM), a targeted proteomics approach that allows the measurement of a predefined peptide set of interest in a highly sensitive and reproducible manner in a complex sample matrix [52, 53], has been employed. Samples from the nine MET-positive cell lines were prepared following METi (24 h treatment), IR or the combination of the two perturbations at two time points (1 h and 8 h post IR) to monitor both early and later signaling events propagated by IR alone or in combination with METi. A total of 116 phosphopeptides (Table S1) involved in DDR, cell cycle regulation, kinase activity and apoptotic processes (Figure S1, Supplementary data 5) were quantified and cell line-specific responses were deconvoluted from group-specific ones (i.e. MET-addicted, METi-responsive and METi-unresponsive) (Supplementary data 1).

**Fig. 2:**
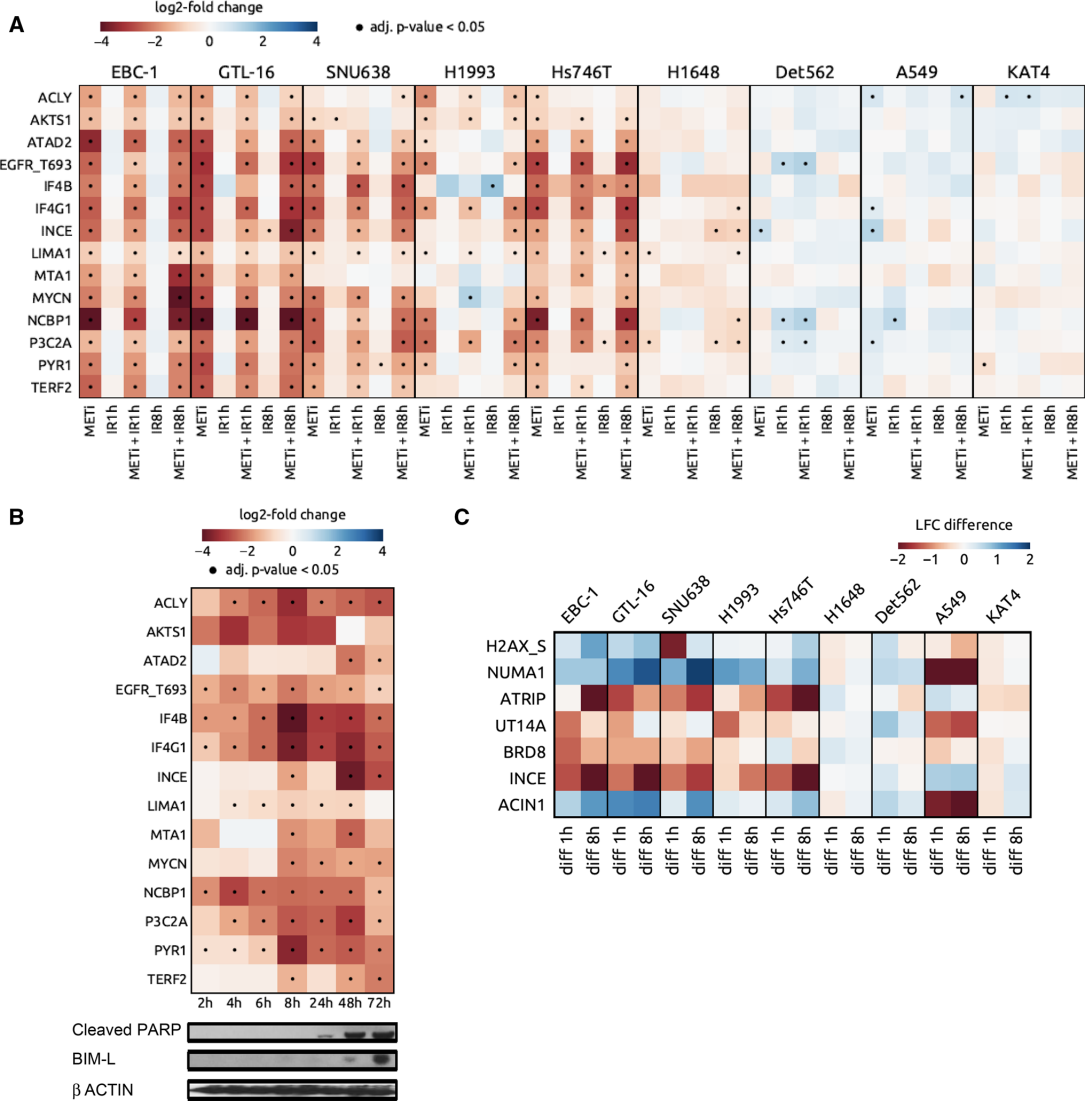
Targeted phosphoproteomics in MET-positive cancer cell lines. **A** Heat map displaying shared phosphorylations emerging in the MET-addicted systems EBC-1 and GTL-16 upon exposure to the MET inhibitor tepotinib (MET addiction phosphorylation signature) and their modulation upon METi, IR and their combination 1 or 8 h post IR across the MET-positive cell line cohort. Blue, upregulated phosphopeptides. Red, downregulated phosphopeptides. Dot, adjusted *p* value < 0.05. **B** Time-dependent emergence of the MET addiction phosphorylation signature in the MET-addicted system EBC-1 upon METi relative to the apoptotic markers cleaved PARP and Bim-L. Blue, upregulated phosphopeptides. Red, downregulated phosphopeptides. Dot, adjusted *p*-value < 0.05. **C** Phosphopeptides commonly regulated in Group I cell lines and displaying significant differences between the treatment by IR and METi + IR

Expectedly, all tested cell lines responded to IR by an increase in phosphorylations on known IR-induced sites, such as NBN S343, SMC1A S957, UT14A S453, TP53B S831, SMC3 S1083, PPM1G S183, ATM S2996 and PRKDC S3205, although the extent of regulation was cell line-dependent (Supplementary data 1). To define shared phosphorylation signatures stemming from the inhibition of the addicting MET oncoprotein, we selected common responses of the two MET-addicted models (Group I) to METi alone, termed them ‘MET addiction phosphorylation signature (APS)’ and studied their regulation across all the cell lines (Groups I-III; Fig. 2A). This signature is composed of 14 phosphorylations that all significantly decrease following METi treatment in Group I cells. Subsets of these phosphorylation events were significantly regulated upon METi in Group II (9–13 phosphosites) whereas only 1–4 significant phosphorylation changes were present in Group III cells, depending on cell line.

To investigate temporal dimension profile of the MET APS, a time-course analysis of METi-induced phosphorylation changes in EBC-1 cells has been performed (Fig. 2B, Supplementary data 2). We could observe significant down-regulation of 5 out of the 14 phophorylations (EGFR T693, IF4B S422, IF4G1 S1231, NCBP1 S22 and PYR1 S1859) in the EBC-1 model already 2 h upon the start of the METi treatment, followed by a significant phosphorylation decrease of ACLY S455, LIMA1 S490 and P3C2A S259 2 h later (Fig. 2B). Eight hours upon METi addition to the media, the entire MET APS with the sole exception of the ATAD2 S327 phosphorylation has been inhibited (Fig. 2B). Importantly, decrease in these phosphorylations is evident considerably earlier than the apoptotic process monitored by cleavage of PARP and BIM-L detected only upon 24 to 48 h of treatment (Fig. 2B). This implies that a significant decrease in these phosphorylations might represent an early indication of the subsequent apoptotic event triggered by METi.

As to the crosstalk between MET signaling and DDR, MET-addicted cell lines shared 7 IR-induced phosphorylation events regulated significantly by the METi + IR treatment as compared to IR alone 1 or 8 h post IR (Fig. 2C). Three of these phosphorylations reported also by Bensimon et al. [41], H2AX S139, NUMA1 S395 and ACIN1 S243, were significantly upregulated, whereas ATRIP S224, UT14A S453, BRD8 S696 and INCE S453 were downregulated upon the combined perturbation (Fig. 2C). Importantly, such regulation of these phosphopeptides was only partially and differentially detected in MET-addicted and METi-responsive but completely absent in METi-unresponsive models (Fig. 2C).

### MET-, EGFR-, ALK- and BRAF-addicted systems share an ‘oncogene addiction phosphorylation signature’

We further investigated the relevance of the phosphorylation signature identified in the context of MET addiction in three additional models of oncogene addiction that have acquired widespread attention in clinical practice: the RTKs EGFR and ALK in NSCLC and the serine/threonine kinase BRAF in melanoma [54–56].

**Fig. 3:**
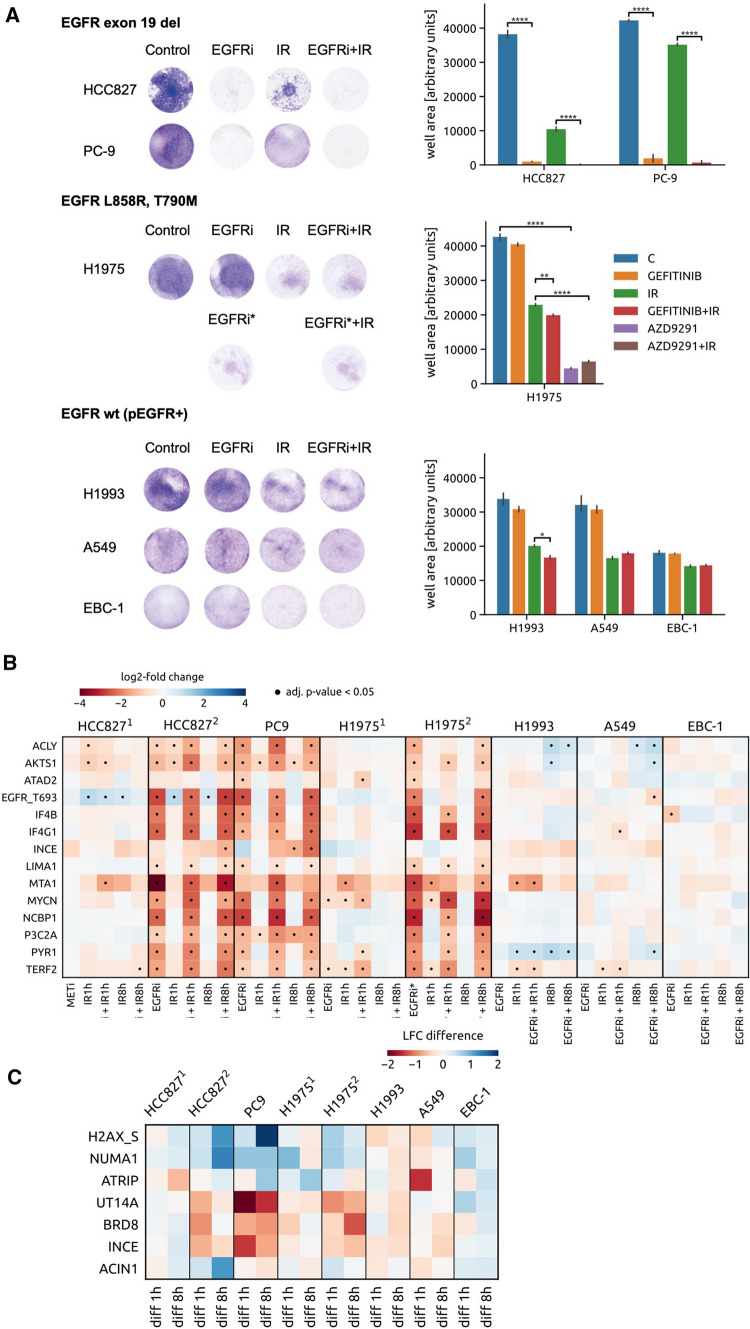
Phenotypic and protein phosphorylation responses of EGFR-positive cancer cell lines towards EGFR targeting. **A** Representative pictures (left) and quantification (right) of viability of a panel of EGFR-positive cells towards EGFR inhibition by gefitinib (EGFRi) or AZD9291 (EGFRi*), IR and their combination. Viability assays were performed to assess sensitivity of EGFR-positive models to EGFRi exerted by gefitinib, IR and the combinatorial treatment; pMET-positive models were exposed to METi, alone and in combination with IR, and the T790M-harboring mutation cells were exposed to third generation EGFR TKI AZD9291, alone and in combinatorial regime with IR. Statistical analysis was performed by GraphPad Prism. *p* values were calculated by Student *t*-test (*P < 0.05; **P < 0.01; ***P < 0.001, ****P < 0.0001). **B** Heat map displaying shared phosphorylations occurring in the EGFR-addicted systems HCC827 (HCC827^2^) and PC-9 upon exposure to the EGFR inhibitor gefitinib (EGFRi) and their modulation upon EGFR inhibition, IR and their combination 1 or 8 h post IR across the EGFR-positive cell line cohort. In addition to the gefitinib treatment (H1975^1^), the H1975 cell line has been exposed also to EGFR inhibition by AZD9291 (EGFRi*; H1975^2^). Apart of the gefitinib treatment (HCC827^2^), MET inhibition by tepotinib was tested in MET- and EGFR-expressing HCC827 cells (HCC827^1^). Blue, upregulated phosphopeptides. Red, downregulated phosphopeptides. Dot, adjusted *p*-value < 0.05. **C** Modulation of DDR-related phosphopeptides in the EGFR-positive cell line panel that were identified in MET-addicted systems as significantly differently regulated between IR and METi + IR condition (Fig. 2C). The heat map displays differences between the treatment by IR and EGFRi + IR assessed in the EGFR-positive cell line panel. To demonstrate the difference between EGFR and MET targeting, effects of the treatments including either METi or EGFRi have been assessed for the HCC827 cell line (HCC827^1^ and the HCC827^2^, respectively)

Since the approval of EGFR TKIs considerably reshaped the therapeutic landscape of NSCLC over the last decade [57, 58], we focused on six NSCLC models expressing either wild-type EGFR (i.e. A549, EBC-1 and H1993) or EGFR-mutated forms (i.e. HCC827, H1975 and PC9) (Table S3). Mutations within the EGFR gene can confer either sensitivity to targeted inhibition of EGFR as in the case of in-frame deletions within exon 19 (harbored by HCC827 and PC9) or feature a major mechanism of resistance to first- and second-generation EGFR TKIs with the T790M gate-keeper mutation (H1975) [59, 60] (Table S3). All EGFR-positive cellular models were exposed to EGFR inhibition (EGFRi) by the first-generation TKI gefitinib alone or in combination with IR (Fig. 3A). In addition, the H1975 cells were treated by the third-generation EGFR TKI AZD9291, which targets the T790M-mutated form of the receptor [61]. Analogously to MET targeting, also EGFRi-responsiveness of cells featuring active EGFR receptor largely differs when assessing their viability post treatment, which presumably depends on their EGFR signaling dependency (Fig. 3A). In that respect, EGFRi alone does not affect A549, EBC-1 and H1993 cell fitness whereas HCC827, PC-9 and H1975 display particularly strong EGFR signaling addiction as inferred from significantly decreased viability in the crystal violet assay (Fig. 3A). Importantly, all the EGFR + cells in our panel also express the MET RTK. The three wild-type EGFR-expressing cell lines feature furthermore constitutive ligand-independent MET activation and represent at the same time either MET-addicted (EBC-1), METi-responsive (H1993) or METi-unresponsive (A549) models. By applying either MET or EGFR inhibition, we could demonstrate that the viability of the EGFR + panel of cell lines differs substantially upon EGFRi or METi with or without IR, illustrating again the problem of determining the correct target in clinical samples solely based on oncogene expression or activation (Figure S2).

To associate these distinct phenotypes with changes in cellular phosphoproteomes, samples originated from cells treated with the anti-EGFR TKIs with and without IR were analyzed by SRM as previously described for the MET-positive cell lines. Figure 3B shows regulation of EGFRi-induced phosphorylation events that are shared by the three EGFR-addicted models (HCC827, PC9 and H1975) amended by MET addiction phosphorylations in all EGFR-expressing cell lines. We could detect significant EGFRi-mediated decrease in 11 out of 14 phosphosites comprising the MET APS in all the EGFR-dependent models (Fig. 3B). Importantly, these phosphorylation events could not be observed upon EGFRi in the EGFR-positive but non-addicted systems such as A549, H1993 and EBC-1 and they were also absent upon METi in the MET-expressing but EGFR-addicted HCC827 model (Fig. 3B). In addition, only EGFR-addicted cells displayed considerable differences in phosphorylations upon IR vs. EGFRi + IR, suggesting that these comprise a feature exclusive to dependency on an oncogene (Fig. 3C).

**Fig. 4:**
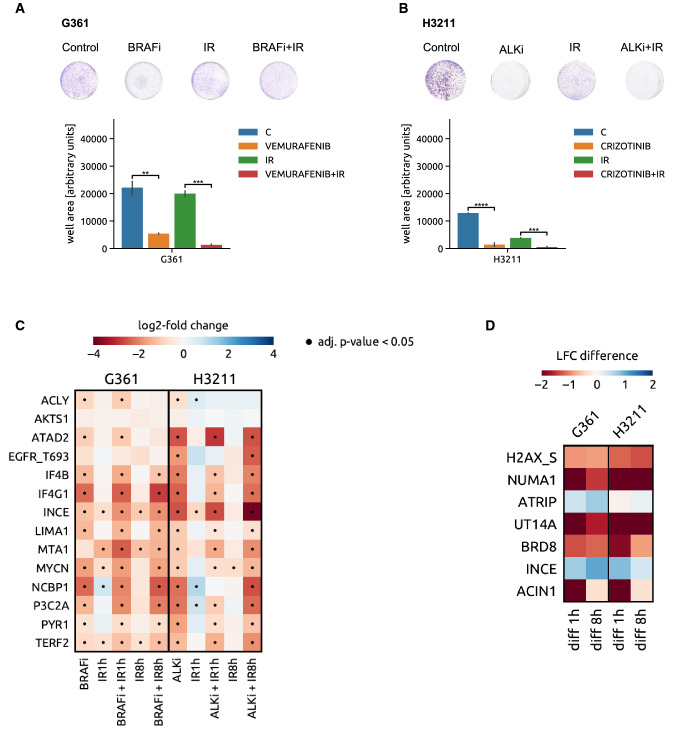
Oncogene targeting-induced responses in ALK- and BRAF-addicted models. **A** Viability of the BRAF V600E-expressing melanoma cell line model G361 upon BRAF inhibition (vemurafenib), IR and their combination (upper panel—representative pictures, lower panel—crystal violet quantification (Student *t*-test, **P < 0.01; ***P < 0.001). **B** Viability of EML4-ALK translocated NSCLC cell line H3211 upon ALK inhibition by crizotinib, IR and their combination (upper panel—representative pictures, lower panel—crystal violet quantification (Student *t*-test, ***P < 0.001, ****P < 0.0001). **C** Heat map of changes in abundance of phosphopeptides that compose the MET oncogene addiction phosphosignature in G361 and H3211 cells upon inhibition of BRAF and ALK, respectively, IR and the combination of the two modalities. *Blue*, upregulated phosphopeptides. Red, downregulated phosphopeptides. Dot, adjusted p-value < 0.05. **D** BRAF and ALK inhibitor-induced modulation (in G361 and H3211 cells, respectively) of DDR-related phosphopeptides that were identified in MET-addicted systems as significantly differently regulated between IR and METi + IR condition (Fig. 2C). The heat map shows a comparison of the phosphorylation levels between BRAFi + IR versus IR alone in the G361 cells (left part) and ALKi + IR versus IR in the H3211 cell line (right panel)

Analogously to METi and EGFRi, the EML4-ALK translocation-based cellular model H3211 was exposed to the ALK inhibitor crizotinib (ALKi) and the BRAF V600E-addicted melanoma cell line G361 to the BRAF inhibitor (BRAFi) vemurafenib (Fig. 4A, B). As shown in Fig. 4C, phosphorylations that are significantly regulated upon inhibition of the addicting oncoprotein in MET- and EGFR-addicted cell lines occur also in the ALK- and BRAF-driven models (11 and 10 out of 14 MET addiction phosphorylations were modulated upon ALK and BRAF inhibition, respectively). The combined treatment modalities also led to significant enhancement of IR-modulated phosphorylation events, reiterating once more the specificity of the signaling at the interface between targeting of the addicting oncogene and DNA damage infliction (Fig. 4D).

**Fig. 5:**
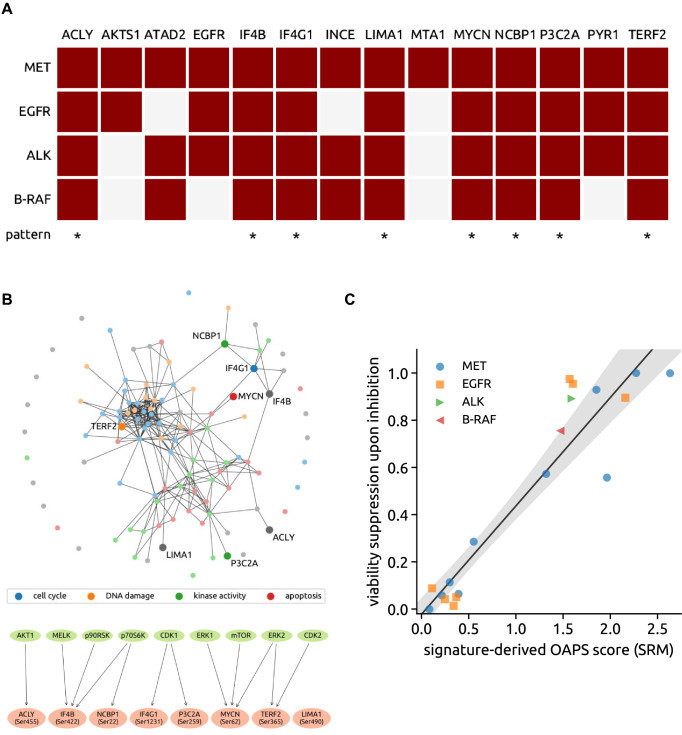
Oncogene addiction phosphorylation signature (OAPS) and the OAPS-derived score. **A** Map of phosphorylation changes elicited by oncogene inhibition in MET-, EGFR-, ALK- and BRAF-addicted cell systems. The 8 phosphopeptides-containing signature shared by all of these models of addiction (*oncogene addiction phosphorylation signature*, OAPS) is visualized in the last row. (Dark red box, downregulation of phosphorylation detected upon inhibition of the respective addicting oncoprotein, white box, significant phosphorylation change not observed.) **B** Upper panel: visualisation of the OAPS-included proteins and their main GO terms cellular functions within the network of all tested phosphopeptides. (GO terms: blue—cell cycle, orange—DNA damage, green—kinase activity, red—apoptosis, gray—other) Lower panel: OAPS phosphosites’s upstream kinases. **C** The viability suppression upon inhibition highly correlates with the signature-derived score computed from phosphoproteomics in cell line systems (correlation 0.94, *p*-value 3 × 10^–10^). The solid line and the shaded area show the linear regression result (intercept − 0.02, slope 0.46, *p*-value 3 × 10^−9^) and its uncertainty (95% CI)

The 8 phosphorylations downregulated upon oncogene inhibition across all the different models for addiction (i.e., MET, EGFR, ALK and BRAF V600E), ACLY S455, IF4B S422, IF4G1 S1231, LIMA1 S490, MYCN S62, NCBP1 S22, P3C2A S259 and TERF2 S365, were collectively termed “oncogene addiction phosphorylation signature” (OAPS; Fig. 5A). Note that this number of phosphorylations downregulated simultaneously in all seven considered inhibitions is of high statistical significance as the expected number of jointly downregulated phosphorylations is only 0.0004 in the null model of independent downregulations (on average, 17% of all 116 phosphorylations are significantly downregulated upon each inhibition).

As shown in Fig. 5B, this signature encompasses distinct cellular processes such as cell cycle, DNA repair or kinase activity and presumably involves the action of upstream kinases such as AKT1, MELK, p90RSK, p70S6K, CDK1, ERK1, mTOR, ERK2 or CDK2. We observed a strong relationship between the phosphoproteomic changes induced by inhibition of each of the four oncogenes and in vitro viability of cell lines [Pearson correlation: 0.94 (*p* value = 3e−09), Spearman correlation: 0.87 (*p* value  = 1e−06)]. We propose a novel score that quantifies this relationship by focusing on the identified signature protein phosphorylations: This “signature-derived score” is the estimated log-fold change of the protein count upon inhibition averaged over all eight signature proteins (Fig. 5C).

These findings suggest that analysis of genomic background of cancer cells may fall short alone to predict responsiveness to selective inhibition of the activity of the expressed oncogenes, supporting the importance of the analysis of the tumor phosphoproteome upon targeting. Thus, in the following we set up to analyze oncogene inhibition-induced phosphoproteome changes utilizing cancer patient specimens.

### A PDX-based ex vivo pipeline validates oncogene addiction phosphorylation signature (OAPS) in NSCLC tumor samples

To investigate whether the postulated OAPS is of a potential clinical relevance, we conducted the SRM analysis in a cohort of 16 tumor biopsies from NSCLC patients. NSCLC tumors often feature heterogenous molecular drivers including MET, EGFR and ALK, rendering these malignancies suitable for validation of oncogene addiction-related changes in protein phosphorylations upon specific inhibition of a potentially addicting oncoprotein.

**Fig. 6:**
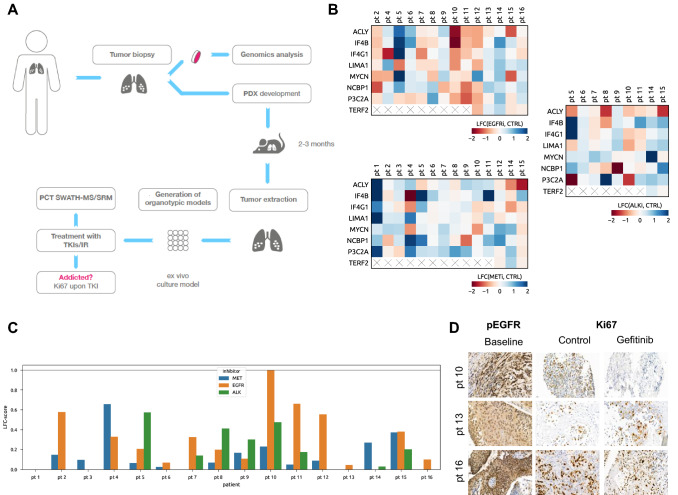
Validation of the oncogene addiction phosphorylation signature (OAPS) in NSCLC tissues. **A** Schematic workflow of the OAPS analysis by SRM in NSCLC tumor biopsies combining PDX models and ex vivo organotypic cultures. **B** Heat maps displaying OAPS changes in NSCLC patient tissues following 16 h of EGFR (gefitinib; upper left panel), MET (tepotinib; lower left panel), and ALK (crizotinib; right panel) inhibition. (*LFC*—log fold change in phosphorylation upon inhibition of addicting oncoprotein; *cross*—phosphopeptide not detected) **C** OAPS-derived score computed from phosphoproteomics in patient tissues (the score for up to three different inhibitors has been assessed for each patient). **D** Representative pictures of immunohistochemical staining (*pEGFR*—EGFR activation; *Ki67*—cell proliferation marker) of selected gefitinib-treated patient tissues (baseline—tissues stained prior start of the treatment, control—untreated tissues (OTCs) collected and stained at day 3, gefitinib—gefitinib-treated OTCs collected and stained at day 3 (48 h upon the start of EGFR inhibition)

We established organotypic cultures (OTCs) derived from NSCLC surgical specimens, treated them ex vivo for 16 h with tepotinib, gefitinib or crizotinib (with and without IR) and subsequently measured perturbation-induced fluctuations in phosphorylations by SRM. As for targeted proteomics measurements on ex vivo-perturbed patient tissues a substantial amount of starting material is required, we employed PDXs as an intermediate step between NSCLC biopsy/resection and ex vivo treatments to expand the available tumor material (Fig. 6A) [62]. In addition, NSCLC OTCs were immunohistochemically stained 3 days after treatment initiation for Ki67 to assess antiproliferative effects of the inhibitors on NSCLC cells as a readout for treatment response. Due to the limited tissue availability, a concomitant immunohistochemistry for total and phosphorylated EGFR, MET or ALK levels was performed only for a subgroup of the cases.

As reported in Fig. 6B, considerable interpatient variability in phosphorylation changes of the 8 proteins representing oncogene-addicted signature upon treatment with the three different inhibitors has been observed. OAPS-derived scores for the 16 NSCLC specimen were ranging from 0 (patient 1 and 7) to 0.65 (patient 4) for tepotinib, 0 (patient 14) to 0.99 (patient 10) for gefitinib and 0 (patient 6) to 0.57 (patient 5) for crizotinib (Fig. 6C). Unfortunately, we could not directly correlate these scores with post-treatment Ki67 levels as in many cases the tissue quality for IHC was substantially compromised. Nevertheless, we have identified a pEGFR-positive NSCLC tumor (patient 10) with a 0.99 gefitinib-treatment score, which showed a 98% reduction in Ki67 levels upon EGFRi, thus suggesting dependency on EGFR to sustain viability (Fig. 6D). In contrast, in two other tumors (patient 13 and 16) with a high expression of phosphorylated EGFR but with low gefitinib-related scores (0.04 and 0.10, respectively), ex vivo-assessed viability upon EGFRi did not decrease (patient 13) or decreased by ca. 30% only (patient 16). These are presumably EGFRi-non-responsive or EGFR-mutated tumors that would likely not profit from gefinitib-directed therapy. Similarly, within our tumor panel we have observed differential responses towards the METi tepotinib (Figure S3), which reflected well the tepotinib-related phosphorylation scores. As demonstrated in Figure S4, the addition of targeted therapy to a DNA damage-based treatment also affects IR-induced DDR phosphorylations in the OTC setting.

Collectively, these observations highlight that the analysis of the phosphorylation signature via OAPS-derived scores may constitute an approach, which assesses responsiveness of clinical samples to targeted perturbations more reliable than genomic profiling.

## Discussion

Massive sequencing efforts of cancer genomes have enabled the documentation of an enormous number of genetic aberrations of thousands of tumors across most of cancer types. However and despite the vast progress associated with cancer genomics and transcriptomics, a further understanding on the level of the functional consequences elicited by the genomic alterations is critically required to optimally guide precision oncology-based therapeutic interventions. Accordingly, a main goal of the current study was to discover and test a phosphoproteomic signature that is associated with tumor responses to the targeting of given driving oncogenes alone and in combination with a DNA-damaging modality.

Inhibitors of RTKs have become a mainstay in the management of various cancers [63–66]. Nevertheless, their tangible success is vastly limited by numerous complex factors spanning all the way from a need for very refined patient stratification to an acquired therapy resistance [66–69]. As to a precise determination of patients that would profit from a given TKI therapy, the presence of the target itself is the most frequently used determinant, followed by its elevated levels or presence of particular driving mutations [66, 70, 71]. These approaches, however, do not seem to be either accurate or sufficient as they are not adequate to always identify the exact tumor driver or set of drivers with the often consequence of limited treatment success or failure. Here we present a strategy, which employs the so-called oncogene addiction phosphorylation signature (OAPS) score, to estimate the likelihood that a particular tumor is addicted to (or driven by) a certain activated oncogene. Furthermore, given the reported observations that link driving kinase oncogenes with DDR signalling [72–75], we were aiming at assessing changes of the selected phoshosites also in the context of IR stress when combined with molecular targeting.

Previously, we have reported that aberrant activation of the MET receptor modulates the cellular response to IR by rewiring key DNA damage response (DDR)-related phosphorylations in some tumor cell lines featuring MET activation [41]. Assuming that a MET–DDR interface underlies MET dependency, here we monitored 116 DDR- and RTK signalling-associated phosphosites in a panel of MET-positive, MET-responsive as well as non-responsive tumor models following targeted MET inhibition. This analysis revealed 14 METi-modulated phosphorylation events that were present solely in MET-addicted models, thus representing ‘MET-as-a-driver’ footprints.

Some of these phosphorylations are regulated through MET downstream effectors. IF4G1 S1231 phosphorylation is controlled by MET through MAPK [76] and downregulation of this phosphosite following METi affects translation of HIF-1α in MET-addicted cellular models under hypoxia [77]. NCBP1 S22, PYR1 S1859 and IF4B S422 were all shown to be regulated through the Ribosomal protein S6 kinase family [78–80], implying the role of MET in controlling cellular metabolism in MET-addicted models. The telomeric binding protein TERF2 S365 and P3C2A S259 sites were previously reported to be phosphorylated by CDK2 [81] and CDK1 [82], respectively, recapitulating the fine regulation exerted by METi on cell cycle progression in METi-responsive systems. Indeed, a differential decay of these phosphorylations over time upon METi seems to fit this molecular scenario.

On the other hand, no putative upstream kinases were reported so far for LIMA1 S490, ATAD2 S327, INCENP S263 or MTA1 T564. Notably, our data seem to indicate a synergism played by several branches propagated by MET and its key downstream effectors, as well as cell cycle-related events in regulating the METi-induced decay in the observed phosphorylations. The identified protein phosphorylation pattern thus appears to represent a composite depiction of METi-driven rewiring processes in MET-addicted systems.

Significant regulation of 8 out of 14 phosphosites (termed ‘oncogene addiction phosphorylation signature’, OAPS) that are modulated by METi in MET-addicted systems was detected also in EGFR-, ALK- and BRAF-addicted cellular tumor models following EGFR, ALK and BRAF targeting, respectively. This observation probably justifies referring to the described OAPS as a generic phospho-signature that is associated with responses of tumor cells to targeting some of the most common driving kinase oncogenes. The presence of the corresponding OAPS distinguished the addicted models from tumor cells, which, although being proficient to these oncogenes, are not driven by them. This observation underlines how different oncoproteins similarly hijack the cellular viability apparatus by controlling shared key signaling nodes.

Interestingly, some of the phosphorylations emerged as specifically characterizing the response of MET-addicted systems to METi, suggesting that, although different addicting oncoproteins assume control of shared key nodes in signaling pathways during tumorigenesis, there could be some crucial differences in subsequent phosphorylation and different roles of downstream targets. For example, ANXA2 Y24, a SRC-regulated site [83], has recently been reported to correlate with invasiveness and metastatic events in pancreatic ductal adenocarcinoma [84], underlining the established role of MET in driving invasive growth during tumorigenesis [85].

Previously, non-targeted MS measurements have been performed by us as well as by others to capture phosphoproteomic changes occurring upon oncogene inhibition in cancer cells. Many of these efforts focused solely on tyrosine phosphorylations [86–88] but in some of the data-dependent acquisition (DDA)-based studies modulations of particular OAPS phosphosites were detected. These included for example TERF2 S365 in PC-9 and H1975 cells following AZD9291 treatment [89] or ACLY Ser455 and NCBP1 Ser22 phosphorylation in erlotinib-treated H1975 cell line [90]. In addition, changes in other phosphorylation sites of OAPS proteins have also been recorded, such as various phosphorylations of EIF4G1, LIMA1, ACLY, EIF4B and TERF2 upon EGFR or MET inhibition [41, 89, 90]. The occurrence of some of the OAPS phosphorylations in these DDA datasets adds to the weight of our SRM-based OAPS signature. At the same time, the absence of the other OAPS components favors the use of a targeted approach to assess oncogene addiction as compared to shotgun proteomics that suffers from missing values which possess additional challenge to data evaluation [91].

Of translational importance, we could show that the presence of the OAPS can be directly monitored in patient tumor biopsies upon distinct targeted treatments ex vivo. In a limited cohort of specimens originating from 16 NSCLC patients, most of them featuring strong EGFR phosphorylation, we could identify a sample (patient 10) with a very high (0.99) OAPS score for EGFR inhibition by gefitinib. We presume that this patient, unlike other patients with strong pEGFR tumor proficiency but with low OAPS gefitinib scores (e.g., patients 13 and 16, for instance) would very likely be a strong responder towards EGFR targeting.

Based on our findings and upon technical adjustments that would facilitate the workflow, OAPS score-based patient stratification could become a plausibly fast and accurate novel precision medicine tool to identify tumor-driving oncoproteins for particular cancers, serving therefore to predict clinical responses towards given targeted interventions. Previous studies have reported favorable characteristics of targeted MS platforms for their application in clinical practice, particularly due to low cost, high specificity, the ability to develop orthogonal assays and multiplexing capabilities [92]. By employing targeted phosphoprotemics via SRM, we could successfully and quantitatively stratify all 16 tumor samples as likely oncogene driven/non-driven based on their phospho-specific responses towards up to 3 different targeted therapies. On the contrary, due to technical limitations and non-sufficient availability of tissue, only a handful of these specimens could be stratified as potentially responsive/non-responsive (but not explicitly driven/non-driven) by IHC-based assessment of Ki-67 positivity.

While the identified OAPS emerges as a generic footmark to pharmacologic inhibition across the four kinases studied in this work, MET, EGFR, ALK and BRAF, its manifestation following combination of kinase targeting with IR appears to be more restrictive and associates primarily with MET and EGFR. In that respect, we can observe for example upregulation or downregulation of phosphorylation on NUMA1 S395 and H2AX S139, and INCENP S263, respectively, following MET inhibition (tepotinib) combined with IR as compared to IR alone in the MET-driven lines EBC-1 and GTL-16. The same trends in significant increases and decreases of these phosphorylations upon IR were also measured for the EGFR-driven lines HCC827, PC9 and H1975 following treatment combination with EGFR targeting. On the other hand, an equivalent regulation of these phosphosites in the G361 and H3211 driven by BRAF and ALK, respectively, was not observed after exposure of the cells to the corresponding inhibitors (vemurafenib and crizotinib, respectively) when combined with IR versus IR alone.

Collectively, our observations suggest that the major element that determines OAPS as a generic treatment response signature is the sensitivity of the tumor to a particular kinase inhibitor while the contribution of IR is more valid in the case of particular oncogenes and less for other cancer drivers. This finding advocates for additional future investigations, primarily, to test if and how the current findings may apply across further targets and tumors.

Pending further successful validation of our approach in extended tumor cohorts and additional cancer entities, we propose that an ex vivo-based phosphoproteomic platform to assess OAPS scores of individual tumors samples could be easily implemented in clinical cancer centers in the future. This would enable a comparably faster and more accurate workflow of patient stratification as compared to genomic or antibody-based techniques determining expression and/or activation of a particular oncogene in current clinical practice.

## Conclusions

Despite enormous progress in cancer genomics and transcriptomics, further functional proteomics readouts are imminently needed to optimally guide precision oncology-based therapeutic interventions. Using targeted proteomics tools within a set of various cancer models we describe here the discovery and characterization of a composite phosphorylation signature associated with tumor addiction to four distinct oncogenes, MET, EGFR, ALK and BRAF.

A score derived from this phospho signature correlates in a robust manner with phenotypic responses of preclinical models as well as treated ex vivo patient tissues towards targeted therapy and can be, therefore, further translated as a base for personalized therapy.

### Supplementary Information

Below is the link to the electronic supplementary material.

Supplementary file1 (PDF 1107 KB)

Supplementary file2 (PDF 73 KB)

Supplementary file3 (PDF 1600 KB)

Supplementary file4 (PDF 58 KB)

Supplementary file5 (TXT 1031 KB)

Supplementary file6 (TXT 144 KB)

Supplementary file7 (TXT 22 KB)

Supplementary file8 (TXT 14 KB)

Supplementary file9 (TXT 1 KB)

## Data Availability

All data generated or analyzed during this study are included in this published article and its supplementary information files.

## Abbreviations

ALK: Anaplastic lymphoma kinase
APS: Addiction phosphorylation signature
ATM: Ataxia telangiectasia-mutated
CDK: Cyclin-dependent kinase
DDA: DNA-damaging agent
DDR: DNA damage response
EGFR: Epidermal growth factor receptor
GO: Gene ontology
HGF: Hepatocyte growth factor
HIF-1α: Hypoxia-inducible factor 1α
IHC: Immunohistochemistry
IR: Ionizing radiation
MAPK: Mitogen-activated protein kinase
MET: Hepatocyte growth factor receptor
MS: Mass spectrometry
NSCLC: Non-small cell lung cancer
OAPS: Oncogene addiction phosphorylation signature
OTC: Organotypic culture
PDX: Patient-derived xenograft
PI3K: Phosphatidylinositol 3-kinase
RTK: Receptor tyrosine kinase
SRM: Selected reaction monitoring
TKI: Tyrosine kinase inhibitor
