# Correction: An oncogene addiction phosphorylation signature and its derived scores inform tumor responsiveness to targeted therapies

**DOI:** 10.1007/s00018-023-04725-8

**Published:** 2023-03-10

**Authors:** Eleonora Orlando, Matúš Medo, Ariel Bensimon, Aurélie Quintin, Rahel Riedo, Selina M. Roth, Carsten Riether, Thomas M. Marti, Daniel M. Aebersold, Michaela Medová, Ruedi Aebersold, Yitzhak Zimmer

**Affiliations:** 1 Department of Radiation Oncology, Inselspital, Bern University Hospital and University of Bern, Bern, Switzerland; 2 Department for BioMedical Research, Radiation Oncology, University of Bern, MEM-E807, Murtenstrasse 35, 3008 Bern, Switzerland; 3 Department of Biology, Institute of Molecular Systems Biology, ETH Zürich, HPM H25, Otto-Stern-Weg 3, 8093 Zurich, Switzerland; 4 Tumorimmunology, Department for BioMedical Research, University of Bern, Bern, Switzerland; 5 Department of Medical Oncology, Inselspital, University Hospital and University of Bern, 3010 Bern, Switzerland; 6 Thoracic Surgery, Department for BioMedical Research, University of Bern, Bern, Switzerland; 7 Division of General Thoracic Surgery, Inselspital Bern University Hospital, Bern, Switzerland; 8 Faculty of Science, University of Zürich, Zurich, Switzerland

**Correction: Cellular and Molecular Life Sciences (2022) 80:6** 10.1007/s00018-022-04634-2

In the published article reference 41 was missed in the third paragraph, under discussion section in the page 15, and the below mentioned reference has been incorrectly processed, and the error in the below references has been now updated.

Previously, we have reported that aberrant activation of the MET receptor modulates the cellular response to IR by rewiring key DNA damage response (DDR)-related phosphorylations in some tumor cell lines featuring MET activation [41]. Assuming that a MET–DDR interface underlies MET dependency, here we monitored 116 DDR- and RTK signalling-associated phosphosites in a panel of MET-positive, MET-responsive as well as non-responsive tumor models following targeted MET inhibition. This analysis revealed 14 METi-modulated phosphorylation events that were present solely in MET-addicted models, thus representing ‘MET-asa-driver’ footprints.

The original article has been updated.
