# Supplementary material for: An oncogene addiction phosphorylation signature and its derived scores inform tumor responsiveness to targeted therapies
Source: Cell Mol Life Sci. 2022 Dec 10;80(1):6. doi: 10.1007/s00018-022-04634-2 (PMC9734221; doi:10.1007/s00018-022-04634-2)
Supplement: Supplementary file 1 — Supplementary file1 (PDF 1107 KB) [file 18_2022_4634_MOESM1_ESM.pdf]

Supplementary figures

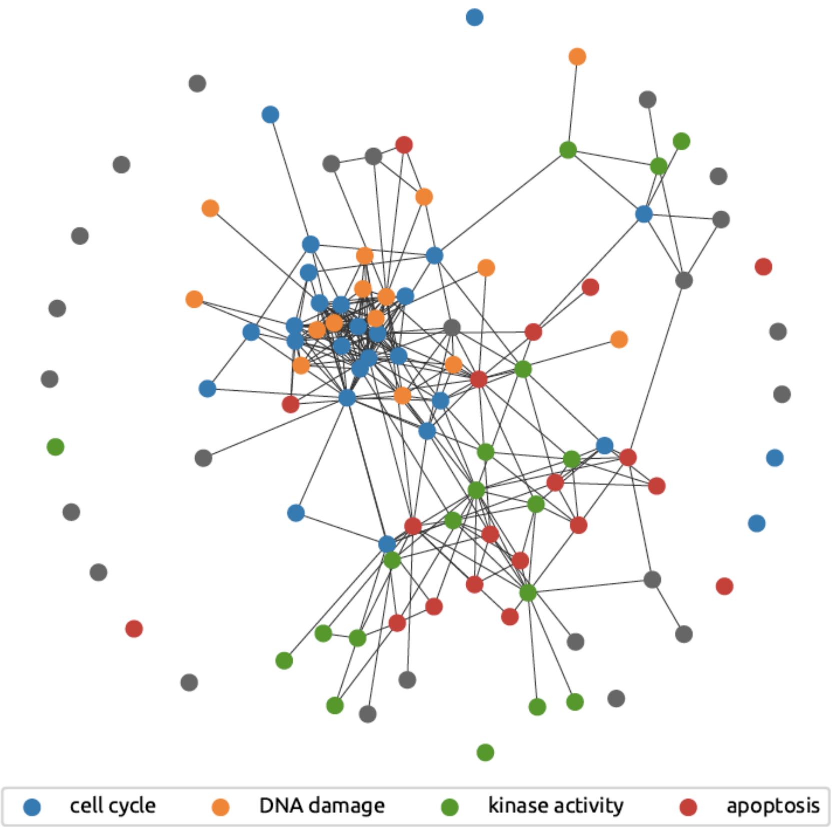

Supplementary figure 1. STRING-based visualization of relationships and functional GO terms of the 104 unique proteins, whose phosphopeptides were analyzed by SRM.

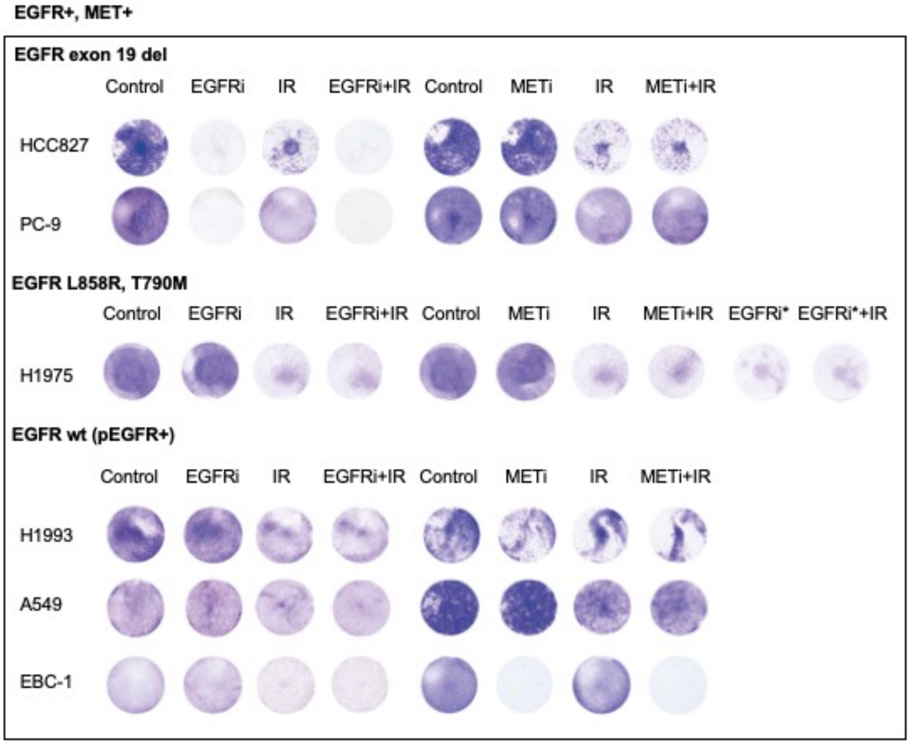

Supplementary figure 2. Representative pictures of viability of a panel of EGFR-positive cells towards EGFR inhibition by gefitinib (EGFRi), AZD9291 (EGFRi*) or EMD1214063 (METi), IR and their combination. Note that parts of the figure have been reproduced from Figure 3A.

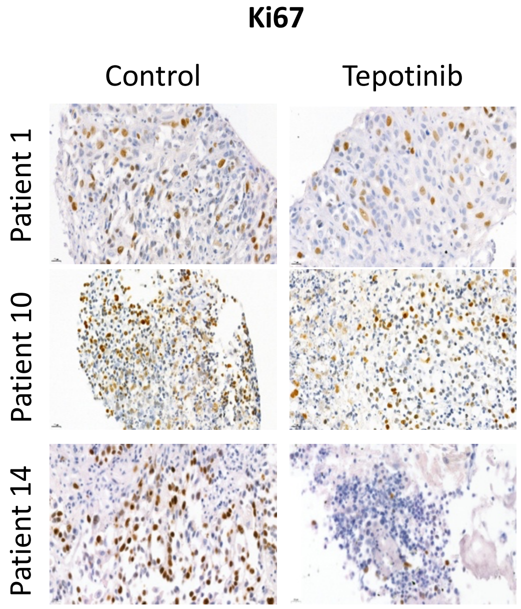

Supplementary figure 3. Representative pictures of immunohistochemical staining (*Ki67* – cell proliferation marker) of selected tepotinib-treated patient tissues (*control* – untreated tissues (OTCs) collected and stained at day 3, *tepotinib* – tepotinib-treated OTCs collected and stained at day 3 (48 hrs upon the start of MET inhibition).

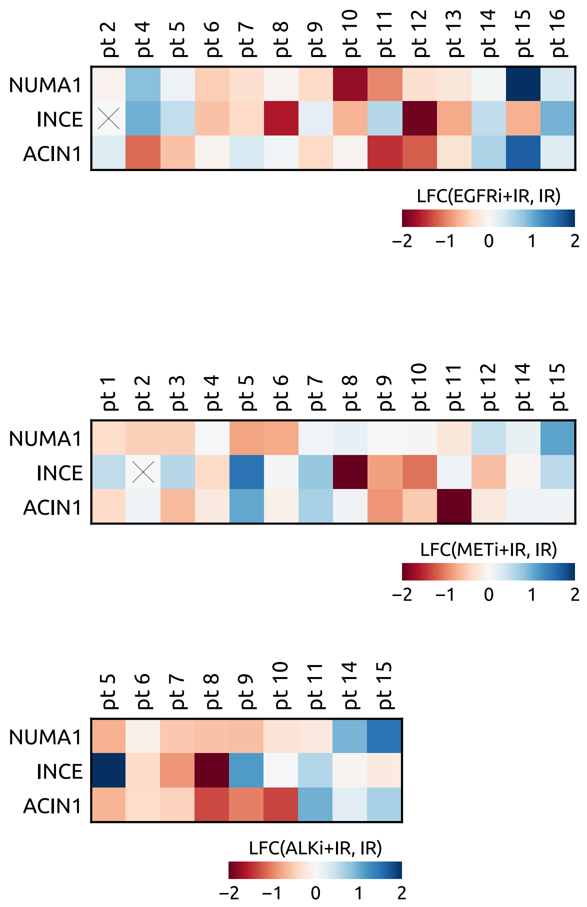

Supplementary figure 4. Heat maps visualizing NUMA1, INCE a ACIN1 phosphorylation levels ratios of EGFRi (gefitinib)+IR- (*top panel*), METi+IR- (*middle panel*) or ALKi+IR- (*low panel*) versus IR-treated patients' tissues.
