# Supplementary material for: An oncogene addiction phosphorylation signature and its derived scores inform tumor responsiveness to targeted therapies
Source: Cell Mol Life Sci. 2022 Dec 10;80(1):6. doi: 10.1007/s00018-022-04634-2 (PMC9734221; doi:10.1007/s00018-022-04634-2)
Supplement: Supplementary file 2 — Supplementary file2 (PDF 73 KB) [file 18_2022_4634_MOESM2_ESM.pdf]

Supplementary Table 1. List of phosphopeptides measured by SRM.

**Protein**	**Site**	**Peptide**
ACIN1	S243	LSEGS(phos)QPAEEEEDQETPSR
ACLY	S455	TAS(phos)FSESR
AIM1	S329	VMPNS(phos)PQNGVLVK
AKTS1	S247	LNTS(phos)DFQK
ANXA2	Y24	LSLEGDHSTPPSAY(phos)GSVK
ANXA2	Y188	RAEDGSVIDY(phos)ELIDQDAR
ARAF	S582	SAS(phos)EPSLHR
ATAD2	S327	KPNIFYSGPAS(phos)PARPR
ATF2	T69T71	NDSVIVADQT(phos)PT(phos)PTR
ATM	S1981	SLAFEEGS(phos)QSTTISSLSEK
ATM	S2996	NLS(phos)DIDQSFNK
ATR	T1989	GVELCFPENET(phos)PPEGK
ATRIP	S224	LAAPSVSHVS(phos)PR
BRAF	S365	SSS(phos)APNVHINTIEPVNIDDLIR
BRCA1	S1239	VNNIPS(phos)QSTR
BRCA2	T703	LQLFIT(phos)PEADSLSCLQEGQCENDPK
BRD8	S696	EESGTIFGS(phos)QIK
CASP8	S219	EQDSES(phos)QTLDK
CDK1	Y15	IGEGT(phos)YGVVYK
CDN1B	S10	VSNGS(phos)PSLER
CHD4	S1349	QVNYNDGS(phos)QEDR
CHK1	S317	VTS(phos)GGVSESPSGFSK
CHK1	S280	YSSS(phos)QPEPR
CHK2	S379	ILGETS(phos)LMR
CIRBP	S146	DYYSSRS(phos)QSGGYSDR
CTNB1	S552	TS(phos)MGGTQQQFVEGVR
DLG3	T759	QT(phos)YEQANK
DOT1L	S1001S1009	NSLPAS(phos)PAHQLSSS(phos)PR
DRG2	Y332	Y(phos)ALVWGTSTK
DYR1A	Y321	IYQY(phos)IQSR
EDC3	T173	HPNQAT(phos)PK
EFHD2	S74	ADLNQGIGEPQS(phos)PSR
EGFR	T693	ELVEPLT(phos)PSGEAPNQALLR
EGFR	Y1197	GSTAENAEY(phos)LR
EI2BE	S544	GGS(phos)PQMDDIK
EMSY	S173	SPRPAS(phos)PASNVVVLPSGSTVYVK
ERBB2	S1054	S(phos)GGGDLTLGLEPSEEEAPR
EZH2	S362S367	LPNNS(phos)SRPST(phos)PTINVLESK
F169A	S350	FQDSEFSSS(phos)QGEDEK
F262	S466	RNS(phos)FTPLSSSNTIR
GSK3A	Y279	GEPNVSY(phos)ICSR

H2AX	S139	KATQAS(phos)QEY
H2AX	T136S139	KAT(phos)QAS(phos)QEY
HDGF	T200	NST(phos)PSEPGSGR
HJURP	S642	LPSS(phos)PLGCR
HN1L	T76	GSGIFDEST(phos)PVQTR
HNRPK	S284	DYDDMS(phos)PR
IF4B	S422	TGS(phos)ESSQTGTSTTSSR
IF4G1	S1231	EAALPPVS(phos)PLK
INCENP	S263	IAQVS(phos)PGPR
IRS1	S1101	HSS(phos)ETFSSTPSATR
JIP4	S251	VSNS(phos)PEPQK
JUND	S73	LAS(phos)PELER
KAPCA	T198	TWT(phos)LCGTPEYLAPEIILSK
KAT5	S90	NGLPGSRPGS(phos)PER
KAT7	S57	LSQSSQDSS(phos)PVR
KI67	S308	SGGSGHAVAEPAS(phos)PEQELDQNK
KS6A1	Y220	AYS(phos)FCGTVEYMAPEVVNR
LAP2	Y1104	AQIPEGDY(phos)LSYR
LARP1	S774	SLPTTVPES(phos)PNYR
LIMA1	S490	ETPHS(phos)PGVEDAPIAK
LTOR5	S26	NPSIVGVLCTDS(phos)QGLNLGCR
MAGG1	S51	DGFAEEAPSTS(phos)R
MCRS1	T103	APST(phos)PVPPSPAPAPGLTK
MET	S1016	ATFPEDQFPNSS(phos)QNGSCR
MET	Y1235	DMYDKEYY(phos)SVHNK
MET	Y1234Y1235	DMYDKEY(phos)Y(phos)SVHNK
MINT	T1947	ELQEAAAVPTT(phos)PR
MISP	S394	ALS(phos)SDSILSPAPDAR
MK03	Y204	IADPEHDHTGFLTEY(phos)VATR
MP2K3	T222	T(phos)MDAGCKPYMAPER
MP2K6	T211	T(phos)IDAGCKPYMAPER
MRGBP	S195	VLTANSNPSS(phos)PSAAK
MTA1	T564	SVSSVLSSLT(phos)PAK
MYCN	S62	FELLPTPPLS(phos)PSR
NBN	S343	TTTPGPSLS(phos)QGVSVDEK
NCBP1	S22	KTS(phos)DANETEDHLESLICK
NPM	T219	DSKPSST(phos)PR
NSUN2	S743	AGEPNS(phos)PDAEEANSPDVTAGCDPAGVHPPR
NUMA1	S395	LSQLEEHLS(phos)QLQDNPPQEK
ODPAT	S293	YHGHS(phos)MSDPGVSYR
P3C2A	S259	VSNLQVS(phos)PK
PARN	S557	NNS(phos)FTAPSTVGK
PDCD4	S457	FVS(phos)EGDGGR
PDCD5	Y80	Y(phos)GQLSEK

PDPK1	S241	ANS(phos)FVGTAQYVSPELLTEK
PML	S527	AVSPPHLDGPPS(phos)PR
PPM1G	S183	SGGGTGEEPGS(phos)QGLNGEAGPEDSTR
PRKDC	S3205	LTPLPEDNS(phos)MNVDQDGDPSDR
PTPRK	S856	YLCEGTES(phos)PYQTGQLHPAIR
PYR1	S1859	IHRAS(phos)DPGLPAEEPK
RAD50	S365	LFDVCGS(phos)QDFESDLDR
RB	S807	IPGGNIYIS(phos)PLK
RPTOR	S863	VLDTSSLTQSAPAS(phos)PTNK
RPTOR	S859S863	VLDTSSLTQS(phos)APAS(phos)PTNK
RS3	T221	DEILPTT(phos)PISEQK
SHC1	Y427	ELFDDPSY(phos)VNVQNLDK
SIR1	S47	S(phos)PGEPGGAAPER
SMC1A	S957	GTMDDISQEEGSS(phos)QGEDSVSGSQR
SMC3	S1083	GSGS(phos)QSSVPSVDQFTGVGIR
STAT3	S727	FICVTPTTCSNTIDLPMS(phos)PR
STAT6	Y641	GY(phos)VPATIK
STMN1	S25	ASGQAFELILS(phos)PR
STMN1	S38	ESVPEFPLS(phos)PPK
TERF2	S365	DLVLPTQALPAS(phos)PALK
TFPT	T207	AGNALT(phos)PELAPVQIK
TIF1B	S473	S(phos)GEGEVSGLMR
TIF1B	S824	FSAVLVEPPPMSLPGAGLSS(phos)QELSGGPGDGP
TP53B	S831	SGTAETEPVEQDSS(phos)QPSLPLVR
TP53B	T855	LDQELQQPQT(phos)QEK
TP53B	S1317	TSS(phos)GTSLSAMHSSGSSGK
TPR	T2137	TVPST(phos)PTLVVPHR
TSC2	S939	STS(phos)LNERPK
UIMC1	S140	GSHIS(phos)QGNEAEER
UT14A	S453	DSGS(phos)QEVLSELR
WDR62	S33	GQSS(phos)PPPAPPICLR
