# Supplementary material for: An oncogene addiction phosphorylation signature and its derived scores inform tumor responsiveness to targeted therapies
Source: Cell Mol Life Sci. 2022 Dec 10;80(1):6. doi: 10.1007/s00018-022-04634-2 (PMC9734221; doi:10.1007/s00018-022-04634-2)
Supplement: Supplementary file 3 — Supplementary file3 (PDF 1600 KB) [file 18_2022_4634_MOESM3_ESM.pdf]

Supplementary table 2. SRM assays parameters				
**731.859827**	854.509428	30.65	sp|Q9Y4K1|AIM1_HUMAN.VMPNS[Pho]PQNGVLVK.+2y8.light	37.7
**731.859827**	515.355159	30.65	sp|Q9Y4K1|AIM1_HUMAN.VMPNS[Pho]PQNGVLVK.+2y5.light	37.7
**731.859827**	458.333696	30.65	sp|Q9Y4K1|AIM1_HUMAN.VMPNS[Pho]PQNGVLVK.+2y4.light	37.7
**731.859827**	359.265282	30.65	sp|Q9Y4K1|AIM1_HUMAN.VMPNS[Pho]PQNGVLVK.+2y3.light	37.7
**731.859827**	616.805378	30.65	sp|Q9Y4K1|AIM1_HUMAN.VMPNS[Pho]PQNGVLVK.+2y11+2.light	37.7
**735.866926**	862.523627	30.65	sp|Q9Y4K1|AIM1_HUMAN.VMPNS[Pho]PQNGVLVK.+2y8.heavy	37.7
**735.866926**	523.369358	30.65	sp|Q9Y4K1|AIM1_HUMAN.VMPNS[Pho]PQNGVLVK.+2y5.heavy	37.7
**735.866926**	466.347895	30.65	sp|Q9Y4K1|AIM1_HUMAN.VMPNS[Pho]PQNGVLVK.+2y4.heavy	37.7
**735.866926**	367.279481	30.65	sp|Q9Y4K1|AIM1_HUMAN.VMPNS[Pho]PQNGVLVK.+2y3.heavy	37.7
**735.866926**	620.812477	30.65	sp|Q9Y4K1|AIM1_HUMAN.VMPNS[Pho]PQNGVLVK.+2y11+2.heavy	37.7
**738.678291**	1000.567337	36.99	sp|Q15910|EZH2_HUMAN.LPNNS[Pho]SRPST[Pho]PTINVLESK.+3y9.light	38.2
**738.678291**	689.382831	36.99	sp|Q15910|EZH2_HUMAN.LPNNS[Pho]SRPST[Pho]PTINVLESK.+3y6.light	38.2
**738.678291**	700.983603	36.99	sp|Q15910|EZH2_HUMAN.LPNNS[Pho]SRPST[Pho]PTINVLESK.+3y18+3.light	38.2
**738.678291**	668.632682	36.99	sp|Q15910|EZH2_HUMAN.LPNNS[Pho]SRPST[Pho]PTINVLESK.+3y17+3.light	38.2
**738.678291**	558.74532	36.99	sp|Q15910|EZH2_HUMAN.LPNNS[Pho]SRPST[Pho]PTINVLESK.+3b10 -98+2.light	38.2
**741.349691**	1008.581536	36.99	sp|Q15910|EZH2_HUMAN.LPNNS[Pho]SRPST[Pho]PTINVLESK.+3y9.heavy	38.2
**741.349691**	697.39703	36.99	sp|Q15910|EZH2_HUMAN.LPNNS[Pho]SRPST[Pho]PTINVLESK.+3y6.heavy	38.2
**741.349691**	703.655003	36.99	sp|Q15910|EZH2_HUMAN.LPNNS[Pho]SRPST[Pho]PTINVLESK.+3y18+3.heavy	38.2
**741.349691**	671.304081	36.99	sp|Q15910|EZH2_HUMAN.LPNNS[Pho]SRPST[Pho]PTINVLESK.+3y17+3.heavy	38.2
**741.349691**	558.74532	36.99	sp|Q15910|EZH2_HUMAN.LPNNS[Pho]SRPST[Pho]PTINVLESK.+3b10 -98+2.heavy	38.2
**798.080585**	937.498923	40.89	sp|Q7Z589|EMSY_HUMAN.SPRPAS[Pho]PASNVVVLPSGSTVYVK.+3y9.light	41.2
**798.080585**	942.479349	40.89	sp|Q7Z589|EMSY_HUMAN.SPRPAS[Pho]PASNVVVLPSGSTVYVK.+3y18+2.light	41.2
**798.080585**	469.2531	40.89	sp|Q7Z589|EMSY_HUMAN.SPRPAS[Pho]PASNVVVLPSGSTVYVK.+3y9+2.light	41.2
**798.080585**	572.76097	40.89	sp|Q7Z589|EMSY_HUMAN.SPRPAS[Pho]PASNVVVLPSGSTVYVK.+3b11+2.light	41.2
**798.080585**	622.295177	40.89	sp|Q7Z589|EMSY_HUMAN.SPRPAS[Pho]PASNVVVLPSGSTVYVK.+3b12+2.light	41.2
**800.751985**	945.513122	40.89	sp|Q7Z589|EMSY_HUMAN.SPRPAS[Pho]PASNVVVLPSGSTVYVK.+3y9.heavy	41.2
**800.751985**	946.486449	40.89	sp|Q7Z589|EMSY_HUMAN.SPRPAS[Pho]PASNVVVLPSGSTVYVK.+3y18+2.heavy	41.2
**800.751985**	473.260199	40.89	sp|Q7Z589|EMSY_HUMAN.SPRPAS[Pho]PASNVVVLPSGSTVYVK.+3y9+2.heavy	41.2
**800.751985**	572.76097	40.89	sp|Q7Z589|EMSY_HUMAN.SPRPAS[Pho]PASNVVVLPSGSTVYVK.+3b11+2.heavy	41.2
**800.751985**	622.295177	40.89	sp|Q7Z589|EMSY_HUMAN.SPRPAS[Pho]PASNVVVLPSGSTVYVK.+3b12+2.heavy	41.2
**603.595791**	747.810538	25.99	sp|Q8TEK3|DOT1L_HUMAN.NSLPAS[Pho]PAHQLSSS[Pho]PR.+3y13+2.light	31.3
**603.595791**	698.82209	25.99	sp|Q8TEK3|DOT1L_HUMAN.NSLPAS[Pho]PAHQLSSS[Pho]PR.+3y13 -98+2.light	31.3
**603.595791**	699.284156	25.99	sp|Q8TEK3|DOT1L_HUMAN.NSLPAS[Pho]PAHQLSSS[Pho]PR.+3y12+2.light	31.3
**603.595791**	498.876117	25.99	sp|Q8TEK3|DOT1L_HUMAN.NSLPAS[Pho]PAHQLSSS[Pho]PR.+3y13+3.light	31.3
**603.595791**	315.166296	25.99	sp|Q8TEK3|DOT1L_HUMAN.NSLPAS[Pho]PAHQLSSS[Pho]PR.+3b3.light	31.3
**606.93188**	752.814673	25.99	sp|Q8TEK3|DOT1L_HUMAN.NSLPAS[Pho]PAHQLSSS[Pho]PR.+3y13+2.heavy	31.3
**606.93188**	703.826225	25.99	sp|Q8TEK3|DOT1L_HUMAN.NSLPAS[Pho]PAHQLSSS[Pho]PR.+3y13 -98+2.heavy	31.3
**606.93188**	704.288291	25.99	sp|Q8TEK3|DOT1L_HUMAN.NSLPAS[Pho]PAHQLSSS[Pho]PR.+3y12+2.heavy	31.3
**606.93188**	502.212207	25.99	sp|Q8TEK3|DOT1L_HUMAN.NSLPAS[Pho]PAHQLSSS[Pho]PR.+3y13+3.heavy	31.3
**606.93188**	315.166296	25.99	sp|Q8TEK3|DOT1L_HUMAN.NSLPAS[Pho]PAHQLSSS[Pho]PR.+3b3.heavy	31.3
**588.969244**	683.408652	30.36	sp|Q96EZ8|MCRS1_HUMAN.APST[Pho]PVPPSPAPAPGLTK.+3y7.light	30.5
**588.969244**	515.318774	30.36	sp|Q96EZ8|MCRS1_HUMAN.APST[Pho]PVPPSPAPAPGLTK.+3y5.light	30.5
**588.969244**	426.252903	30.36	sp|Q96EZ8|MCRS1_HUMAN.APST[Pho]PVPPSPAPAPGLTK.+3y9+2.light	30.5
**588.969244**	342.207964	30.36	sp|Q96EZ8|MCRS1_HUMAN.APST[Pho]PVPPSPAPAPGLTK.+3y7+2.light	30.5
**588.969244**	633.264369	30.36	sp|Q96EZ8|MCRS1_HUMAN.APST[Pho]PVPPSPAPAPGLTK.+3b6.light	30.5
**591.640643**	691.422851	30.36	sp|Q96EZ8|MCRS1_HUMAN.APST[Pho]PVPPSPAPAPGLTK.+3y7.heavy	30.5
**591.640643**	523.332973	30.36	sp|Q96EZ8|MCRS1_HUMAN.APST[Pho]PVPPSPAPAPGLTK.+3y5.heavy	30.5
**591.640643**	430.260002	30.36	sp|Q96EZ8|MCRS1_HUMAN.APST[Pho]PVPPSPAPAPGLTK.+3y9+2.heavy	30.5
**591.640643**	346.215063	30.36	sp|Q96EZ8|MCRS1_HUMAN.APST[Pho]PVPPSPAPAPGLTK.+3y7+2.heavy	30.5
**591.640643**	633.264369	30.36	sp|Q96EZ8|MCRS1_HUMAN.APST[Pho]PVPPSPAPAPGLTK.+3b6.heavy	30.5
**762.358703**	824.354975	18.1	sp|Q9NV56|MRGBP_HUMAN.VLTANSNPSS[Pho]PSAAK.+2y8.light	39
**762.358703**	726.378079	18.1	sp|Q9NV56|MRGBP_HUMAN.VLTANSNPSS[Pho]PSAAK.+2y8 -98.light	39
**762.358703**	727.302212	18.1	sp|Q9NV56|MRGBP_HUMAN.VLTANSNPSS[Pho]PSAAK.+2y7.light	39
**762.358703**	473.271824	18.1	sp|Q9NV56|MRGBP_HUMAN.VLTANSNPSS[Pho]PSAAK.+2y5.light	39

**762.358703**	656.282464	18.1	sp|Q9NV56|MRGBP_HUMAN.VLTANSNPSS[Pho]PSAAK.+2y13+2.light	39
**766.365802**	832.369174	18.1	sp|Q9NV56|MRGBP_HUMAN.VLTANSNPSS[Pho]PSAAK.+2y8.heavy	39
**766.365802**	734.392278	18.1	sp|Q9NV56|MRGBP_HUMAN.VLTANSNPSS[Pho]PSAAK.+2y8 -98.heavy	39
**766.365802**	735.316411	18.1	sp|Q9NV56|MRGBP_HUMAN.VLTANSNPSS[Pho]PSAAK.+2y7.heavy	39
**766.365802**	481.286023	18.1	sp|Q9NV56|MRGBP_HUMAN.VLTANSNPSS[Pho]PSAAK.+2y5.heavy	39
**766.365802**	660.289563	18.1	sp|Q9NV56|MRGBP_HUMAN.VLTANSNPSS[Pho]PSAAK.+2y13+2.heavy	39
**678.344533**	896.448876	39.56	sp|Q13330|MTA1_HUMAN.SVSSVLSSLT[Pho]PAK.+2y8.light	35.3
**678.344533**	783.364812	39.56	sp|Q13330|MTA1_HUMAN.SVSSVLSSLT[Pho]PAK.+2y7.light	35.3
**678.344533**	696.332783	39.56	sp|Q13330|MTA1_HUMAN.SVSSVLSSLT[Pho]PAK.+2y6.light	35.3
**678.344533**	398.239795	39.56	sp|Q13330|MTA1_HUMAN.SVSSVLSSLT[Pho]PAK.+2y4 -98.light	35.3
**678.344533**	315.202681	39.56	sp|Q13330|MTA1_HUMAN.SVSSVLSSLT[Pho]PAK.+2y3.light	35.3
**682.351632**	904.463075	39.56	sp|Q13330|MTA1_HUMAN.SVSSVLSSLT[Pho]PAK.+2y8.heavy	35.3
**682.351632**	791.379011	39.56	sp|Q13330|MTA1_HUMAN.SVSSVLSSLT[Pho]PAK.+2y7.heavy	35.3
**682.351632**	704.346982	39.56	sp|Q13330|MTA1_HUMAN.SVSSVLSSLT[Pho]PAK.+2y6.heavy	35.3
**682.351632**	406.253994	39.56	sp|Q13330|MTA1_HUMAN.SVSSVLSSLT[Pho]PAK.+2y4 -98.heavy	35.3
**682.351632**	323.21688	39.56	sp|Q13330|MTA1_HUMAN.SVSSVLSSLT[Pho]PAK.+2y3.heavy	35.3
**495.260469**	607.367455	32.16	sp|P12270|TPR_HUMAN.TVPST[Pho]PTLVVPHR.+3y5.light	25.8
**495.260469**	508.299041	32.16	sp|P12270|TPR_HUMAN.TVPST[Pho]PTLVVPHR.+3y4.light	25.8
**495.260469**	409.230627	32.16	sp|P12270|TPR_HUMAN.TVPST[Pho]PTLVVPHR.+3y3.light	25.8
**495.260469**	642.32902	32.16	sp|P12270|TPR_HUMAN.TVPST[Pho]PTLVVPHR.+3y11+2.light	25.8
**495.260469**	428.555105	32.16	sp|P12270|TPR_HUMAN.TVPST[Pho]PTLVVPHR.+3y11+3.light	25.8
**498.596559**	617.375724	32.16	sp|P12270|TPR_HUMAN.TVPST[Pho]PTLVVPHR.+3y5.heavy	25.8
**498.596559**	518.30731	32.16	sp|P12270|TPR_HUMAN.TVPST[Pho]PTLVVPHR.+3y4.heavy	25.8
**498.596559**	419.238896	32.16	sp|P12270|TPR_HUMAN.TVPST[Pho]PTLVVPHR.+3y3.heavy	25.8
**498.596559**	647.333154	32.16	sp|P12270|TPR_HUMAN.TVPST[Pho]PTLVVPHR.+3y11+2.heavy	25.8
**498.596559**	431.891195	32.16	sp|P12270|TPR_HUMAN.TVPST[Pho]PTLVVPHR.+3y11+3.heavy	25.8
**603.235488**	654.284179	19.6	sp|Q14011|CIRBP_HUMAN.DYYSSRS[Pho]QSGGYSDR.+3y6.light	31.3
**603.235488**	765.304459	19.6	sp|Q14011|CIRBP_HUMAN.DYYSSRS[Pho]QSGGYSDR.+3y13+2.light	31.3
**603.235488**	716.316011	19.6	sp|Q14011|CIRBP_HUMAN.DYYSSRS[Pho]QSGGYSDR.+3y13 -98+2.light	31.3
**603.235488**	683.772794	19.6	sp|Q14011|CIRBP_HUMAN.DYYSSRS[Pho]QSGGYSDR.+3y12+2.light	31.3
**603.235488**	564.893174	19.6	sp|Q14011|CIRBP_HUMAN.DYYSSRS[Pho]QSGGYSDR.+3y14+3.light	31.3
**606.571578**	664.292448	19.6	sp|Q14011|CIRBP_HUMAN.DYYSSRS[Pho]QSGGYSDR.+3y6.heavy	31.3
**606.571578**	770.308593	19.6	sp|Q14011|CIRBP_HUMAN.DYYSSRS[Pho]QSGGYSDR.+3y13+2.heavy	31.3
**606.571578**	721.320145	19.6	sp|Q14011|CIRBP_HUMAN.DYYSSRS[Pho]QSGGYSDR.+3y13 -98+2.heavy	31.3
**606.571578**	688.776929	19.6	sp|Q14011|CIRBP_HUMAN.DYYSSRS[Pho]QSGGYSDR.+3y12+2.heavy	31.3
**606.571578**	568.229264	19.6	sp|Q14011|CIRBP_HUMAN.DYYSSRS[Pho]QSGGYSDR.+3y14+3.heavy	31.3
**486.718574**	835.37096	2.91	sp|Q96F86|EDC3_HUMAN.HPNQAT[Pho]PK.+2y7.light	26.9
**486.718574**	737.394064	2.91	sp|Q96F86|EDC3_HUMAN.HPNQAT[Pho]PK.+2y7 -98.light	26.9
**486.718574**	738.318196	2.91	sp|Q96F86|EDC3_HUMAN.HPNQAT[Pho]PK.+2y6.light	26.9
**486.718574**	425.179577	2.91	sp|Q96F86|EDC3_HUMAN.HPNQAT[Pho]PK.+2y3.light	26.9
**486.718574**	418.189118	2.91	sp|Q96F86|EDC3_HUMAN.HPNQAT[Pho]PK.+2y7+2.light	26.9
**490.725673**	843.385159	2.91	sp|Q96F86|EDC3_HUMAN.HPNQAT[Pho]PK.+2y7.heavy	26.9
**490.725673**	745.408263	2.91	sp|Q96F86|EDC3_HUMAN.HPNQAT[Pho]PK.+2y7 -98.heavy	26.9
**490.725673**	746.332395	2.91	sp|Q96F86|EDC3_HUMAN.HPNQAT[Pho]PK.+2y6.heavy	26.9
**490.725673**	433.193776	2.91	sp|Q96F86|EDC3_HUMAN.HPNQAT[Pho]PK.+2y3.heavy	26.9
**490.725673**	422.196217	2.91	sp|Q96F86|EDC3_HUMAN.HPNQAT[Pho]PK.+2y7+2.heavy	26.9
**487.256705**	860.42207	13.85	iRT_peptides.LGGNETQVR.+2y8.light	26.9
**487.256705**	803.400606	13.85	iRT_peptides.LGGNETQVR.+2y7.light	26.9
**487.256705**	503.293622	13.85	iRT_peptides.LGGNETQVR.+2y4.light	26.9
**547.298038**	817.441408	30.97	iRT_peptides.YILAGVESNK.+2y8.light	29.6
**547.298038**	704.357344	30.97	iRT_peptides.YILAGVESNK.+2y7.light	29.6
**547.298038**	633.32023	30.97	iRT_peptides.YILAGVESNK.+2y6.light	29.6
**644.822606**	1016.525866	23.42	iRT_peptides.AGGSSEPVTGLADK.+2y10.light	33.9
**644.822606**	800.451245	23.42	iRT_peptides.AGGSSEPVTGLADK.+2y8.light	33.9
**644.822606**	604.330067	23.42	iRT_peptides.AGGSSEPVTGLADK.+2y6.light	33.9
**669.838059**	1041.499986	33.03	iRT_peptides.TPVISGGPYYER.+2y9.light	35
**669.838059**	928.415922	33.03	iRT_peptides.TPVISGGPYYER.+2y8.light	35

**669.838059**	841.383893	33.03	iRT_peptides.TPVISGGPYYER.+2y7.light	35
**683.827888**	819.38831	27.68	iRT_peptides.VEATFGVDESANK.+2b8.light	35.6
**683.827888**	966.452701	27.68	iRT_peptides.VEATFGVDESANK.+2y9.light	35.6
**683.827888**	663.29441	27.68	iRT_peptides.VEATFGVDESANK.+2y6.light	35.6
**683.853709**	1069.531286	34.61	iRT_peptides.TPVITGAPYYER.+2y9.light	35.6
**683.853709**	956.447222	34.61	iRT_peptides.TPVITGAPYYER.+2y8.light	35.6
**683.853709**	855.399543	34.61	iRT_peptides.TPVITGAPYYER.+2y7.light	35.6
**726.835713**	1066.484001	41.52	iRT_peptides.DAVTPADFSEWSK.+2y9.light	37.5
**726.835713**	584.269478	41.52	iRT_peptides.DAVTPADFSEWSK.+2y10+2.light	37.5
**726.835713**	533.745639	41.52	iRT_peptides.DAVTPADFSEWSK.+2y9+2.light	37.5
**622.853512**	826.478128	47.87	iRT_peptides.TGFIIDPGGVIR.+2y8.light	32.9
**622.853512**	713.394064	47.87	iRT_peptides.TGFIIDPGGVIR.+2y7.light	32.9
**622.853512**	598.367121	47.87	iRT_peptides.TGFIIDPGGVIR.+2y6.light	32.9
**636.869162**	854.509428	51.07	iRT_peptides.GTFIIDPAAIVR.+2y8.light	33.5
**636.869162**	741.425364	51.07	iRT_peptides.GTFIIDPAAIVR.+2y7.light	33.5
**636.869162**	626.398421	51.07	iRT_peptides.GTFIIDPAAIVR.+2y6.light	33.5
**699.338423**	926.473043	37.16	iRT_peptides.GDLDAASYYAPVR.+2y8.light	36.3
**699.338423**	855.435929	37.16	iRT_peptides.GDLDAASYYAPVR.+2y7.light	36.3
**699.338423**	605.340572	37.16	iRT_peptides.GDLDAASYYAPVR.+2y5.light	36.3
**776.929751**	1051.557107	53.85	iRT_peptides.FLLQFGAQGSPLFK.+2y10.light	39.7
**776.929751**	904.488693	53.85	iRT_peptides.FLLQFGAQGSPLFK.+2y9.light	39.7
**776.929751**	504.318046	53.85	iRT_peptides.FLLQFGAQGSPLFK.+2y4.light	39.7
**519.581214**	755.423256	35.54	sp|O43379|WDR62_HUMAN.GQSS[Pho]PPPAPPIC[CAM]LR.+3y6.light	27
**519.581214**	658.370492	35.54	sp|O43379|WDR62_HUMAN.GQSS[Pho]PPPAPPIC[CAM]LR.+3y5.light	27
**519.581214**	378.215266	35.54	sp|O43379|WDR62_HUMAN.GQSS[Pho]PPPAPPIC[CAM]LR.+3y6+2.light	27
**519.581214**	802.313111	35.54	sp|O43379|WDR62_HUMAN.GQSS[Pho]PPPAPPIC[CAM]LR.+3b8.light	27
**519.581214**	704.336215	35.54	sp|O43379|WDR62_HUMAN.GQSS[Pho]PPPAPPIC[CAM]LR.+3b8 -98.light	27
**522.917304**	765.431525	35.54	sp|O43379|WDR62_HUMAN.GQSS[Pho]PPPAPPIC[CAM]LR.+3y6.heavy	27
**522.917304**	668.378761	35.54	sp|O43379|WDR62_HUMAN.GQSS[Pho]PPPAPPIC[CAM]LR.+3y5.heavy	27
**522.917304**	383.219401	35.54	sp|O43379|WDR62_HUMAN.GQSS[Pho]PPPAPPIC[CAM]LR.+3y6+2.heavy	27
**522.917304**	802.313111	35.54	sp|O43379|WDR62_HUMAN.GQSS[Pho]PPPAPPIC[CAM]LR.+3b8.heavy	27
**522.917304**	704.336215	35.54	sp|O43379|WDR62_HUMAN.GQSS[Pho]PPPAPPIC[CAM]LR.+3b8 -98.heavy	27
**685.80102**	1042.420095	12.79	sp|O95251|KAT7_HUMAN.LSQSSQDSS[Pho]PVR.+2y9.light	35.7
**685.80102**	740.297461	12.79	sp|O95251|KAT7_HUMAN.LSQSSQDSS[Pho]PVR.+2y6.light	35.7
**685.80102**	625.270517	12.79	sp|O95251|KAT7_HUMAN.LSQSSQDSS[Pho]PVR.+2y5.light	35.7
**685.80102**	371.24013	12.79	sp|O95251|KAT7_HUMAN.LSQSSQDSS[Pho]PVR.+2y3.light	35.7
**685.80102**	629.258988	12.79	sp|O95251|KAT7_HUMAN.LSQSSQDSS[Pho]PVR.+2y11+2.light	35.7
**690.805155**	1052.428364	12.79	sp|O95251|KAT7_HUMAN.LSQSSQDSS[Pho]PVR.+2y9.heavy	35.7
**690.805155**	750.30573	12.79	sp|O95251|KAT7_HUMAN.LSQSSQDSS[Pho]PVR.+2y6.heavy	35.7
**690.805155**	635.278786	12.79	sp|O95251|KAT7_HUMAN.LSQSSQDSS[Pho]PVR.+2y5.heavy	35.7
**690.805155**	381.248399	12.79	sp|O95251|KAT7_HUMAN.LSQSSQDSS[Pho]PVR.+2y3.heavy	35.7
**690.805155**	634.263123	12.79	sp|O95251|KAT7_HUMAN.LSQSSQDSS[Pho]PVR.+2y11+2.heavy	35.7
**988.437878**	929.505071	44.99	sp|P29353|SHC1_HUMAN.ELFDDPSY[Pho]VNVQNLDK.+2y8.light	49
**988.437878**	830.436657	44.99	sp|P29353|SHC1_HUMAN.ELFDDPSY[Pho]VNVQNLDK.+2y7.light	49
**988.437878**	617.325316	44.99	sp|P29353|SHC1_HUMAN.ELFDDPSY[Pho]VNVQNLDK.+2y5.light	49
**988.437878**	489.266738	44.99	sp|P29353|SHC1_HUMAN.ELFDDPSY[Pho]VNVQNLDK.+2y4.light	49
**988.437878**	678.8134	44.99	sp|P29353|SHC1_HUMAN.ELFDDPSY[Pho]VNVQNLDK.+2y11+2.light	49
**992.444978**	937.51927	44.99	sp|P29353|SHC1_HUMAN.ELFDDPSY[Pho]VNVQNLDK.+2y8.heavy	49
**992.444978**	838.450856	44.99	sp|P29353|SHC1_HUMAN.ELFDDPSY[Pho]VNVQNLDK.+2y7.heavy	49
**992.444978**	625.339515	44.99	sp|P29353|SHC1_HUMAN.ELFDDPSY[Pho]VNVQNLDK.+2y5.heavy	49
**992.444978**	497.280937	44.99	sp|P29353|SHC1_HUMAN.ELFDDPSY[Pho]VNVQNLDK.+2y4.heavy	49
**992.444978**	682.820499	44.99	sp|P29353|SHC1_HUMAN.ELFDDPSY[Pho]VNVQNLDK.+2y11+2.heavy	49
**676.354703**	800.395384	42.52	sp|P06400|RB_HUMAN.IPGGNIYIS[Pho]PLK.+2y6.light	35.3
**676.354703**	357.249632	42.52	sp|P06400|RB_HUMAN.IPGGNIYIS[Pho]PLK.+2y3.light	35.3
**676.354703**	619.812671	42.52	sp|P06400|RB_HUMAN.IPGGNIYIS[Pho]PLK.+2y11+2.light	35.3
**676.354703**	571.286289	42.52	sp|P06400|RB_HUMAN.IPGGNIYIS[Pho]PLK.+2y10+2.light	35.3
**676.354703**	439.229959	42.52	sp|P06400|RB_HUMAN.IPGGNIYIS[Pho]PLK.+2b5.light	35.3

**680.361803**	808.409583	42.52	sp|P06400|RB_HUMAN.IPGGNIYIS[Pho]PLK.+2y6.heavy	35.3
**680.361803**	365.263831	42.52	sp|P06400|RB_HUMAN.IPGGNIYIS[Pho]PLK.+2y3.heavy	35.3
**680.361803**	623.819771	42.52	sp|P06400|RB_HUMAN.IPGGNIYIS[Pho]PLK.+2y11+2.heavy	35.3
**680.361803**	575.293389	42.52	sp|P06400|RB_HUMAN.IPGGNIYIS[Pho]PLK.+2y10+2.heavy	35.3
**680.361803**	439.229959	42.52	sp|P06400|RB_HUMAN.IPGGNIYIS[Pho]PLK.+2b5.heavy	35.3
**758.037217**	799.492381	54.32	sp|P17612|KAPCA_HUMAN.TWT[Pho]LC[CAM]GTPEYLAPEIILSK.+3y7.light	39.2
**758.037217**	460.31296	54.32	sp|P17612|KAPCA_HUMAN.TWT[Pho]LC[CAM]GTPEYLAPEIILSK.+3y4.light	39.2
**758.037217**	347.228896	54.32	sp|P17612|KAPCA_HUMAN.TWT[Pho]LC[CAM]GTPEYLAPEIILSK.+3y3.light	39.2
**758.037217**	400.249829	54.32	sp|P17612|KAPCA_HUMAN.TWT[Pho]LC[CAM]GTPEYLAPEIILSK.+3y7+2.light	39.2
**758.037217**	469.148277	54.32	sp|P17612|KAPCA_HUMAN.TWT[Pho]LC[CAM]GTPEYLAPEIILSK.+3b3.light	39.2
**760.708617**	807.50658	54.32	sp|P17612|KAPCA_HUMAN.TWT[Pho]LC[CAM]GTPEYLAPEIILSK.+3y7.heavy	39.2
**760.708617**	468.327159	54.32	sp|P17612|KAPCA_HUMAN.TWT[Pho]LC[CAM]GTPEYLAPEIILSK.+3y4.heavy	39.2
**760.708617**	355.243095	54.32	sp|P17612|KAPCA_HUMAN.TWT[Pho]LC[CAM]GTPEYLAPEIILSK.+3y3.heavy	39.2
**760.708617**	404.256928	54.32	sp|P17612|KAPCA_HUMAN.TWT[Pho]LC[CAM]GTPEYLAPEIILSK.+3y7+2.heavy	39.2
**760.708617**	469.148277	54.32	sp|P17612|KAPCA_HUMAN.TWT[Pho]LC[CAM]GTPEYLAPEIILSK.+3b3.heavy	39.2
**598.276984**	755.30836	16.3	sp|P38398|BRCA1_HUMAN.VNNIPS[Pho]QSTR.+2y6.light	31.8
**598.276984**	658.255596	16.3	sp|P38398|BRCA1_HUMAN.VNNIPS[Pho]QSTR.+2y5.light	31.8
**598.276984**	378.157818	16.3	sp|P38398|BRCA1_HUMAN.VNNIPS[Pho]QSTR.+2y6+2.light	31.8
**598.276984**	328.161545	16.3	sp|P38398|BRCA1_HUMAN.VNNIPS[Pho]QSTR.+2b3.light	31.8
**598.276984**	441.245609	16.3	sp|P38398|BRCA1_HUMAN.VNNIPS[Pho]QSTR.+2b4.light	31.8
**603.281119**	765.316629	16.3	sp|P38398|BRCA1_HUMAN.VNNIPS[Pho]QSTR.+2y6.heavy	31.8
**603.281119**	668.263865	16.3	sp|P38398|BRCA1_HUMAN.VNNIPS[Pho]QSTR.+2y5.heavy	31.8
**603.281119**	383.161952	16.3	sp|P38398|BRCA1_HUMAN.VNNIPS[Pho]QSTR.+2y6+2.heavy	31.8
**603.281119**	328.161545	16.3	sp|P38398|BRCA1_HUMAN.VNNIPS[Pho]QSTR.+2b3.heavy	31.8
**603.281119**	441.245609	16.3	sp|P38398|BRCA1_HUMAN.VNNIPS[Pho]QSTR.+2b4.heavy	31.8
**532.232045**	738.389313	16.2	sp|P10398|ARAF_HUMAN.SAS[Pho]EPSLHR.+2y6.light	28.9
**532.232045**	609.34672	16.2	sp|P10398|ARAF_HUMAN.SAS[Pho]EPSLHR.+2y5.light	28.9
**532.232045**	512.293956	16.2	sp|P10398|ARAF_HUMAN.SAS[Pho]EPSLHR.+2y4.light	28.9
**532.232045**	305.176998	16.2	sp|P10398|ARAF_HUMAN.SAS[Pho]EPSLHR.+2y5+2.light	28.9
**532.232045**	326.074778	16.2	sp|P10398|ARAF_HUMAN.SAS[Pho]EPSLHR.+2b3.light	28.9
**537.23618**	748.397582	16.2	sp|P10398|ARAF_HUMAN.SAS[Pho]EPSLHR.+2y6.heavy	28.9
**537.23618**	619.354989	16.2	sp|P10398|ARAF_HUMAN.SAS[Pho]EPSLHR.+2y5.heavy	28.9
**537.23618**	522.302225	16.2	sp|P10398|ARAF_HUMAN.SAS[Pho]EPSLHR.+2y4.heavy	28.9
**537.23618**	310.181132	16.2	sp|P10398|ARAF_HUMAN.SAS[Pho]EPSLHR.+2y5+2.heavy	28.9
**537.23618**	326.074778	16.2	sp|P10398|ARAF_HUMAN.SAS[Pho]EPSLHR.+2b3.heavy	28.9
**642.294456**	985.439039	28.72	sp|P07355|ANXA2_HUMAN.LSLEGDHSTPPSAY[Pho]GSVK.+3y9.light	33.3
**642.294456**	888.386276	28.72	sp|P07355|ANXA2_HUMAN.LSLEGDHSTPPSAY[Pho]GSVK.+3y8.light	33.3
**642.294456**	906.396013	28.72	sp|P07355|ANXA2_HUMAN.LSLEGDHSTPPSAY[Pho]GSVK.+3y17+2.light	33.3
**642.294456**	862.879999	28.72	sp|P07355|ANXA2_HUMAN.LSLEGDHSTPPSAY[Pho]GSVK.+3y16+2.light	33.3
**642.294456**	493.223158	28.72	sp|P07355|ANXA2_HUMAN.LSLEGDHSTPPSAY[Pho]GSVK.+3y9+2.light	33.3
**644.965855**	993.453238	28.72	sp|P07355|ANXA2_HUMAN.LSLEGDHSTPPSAY[Pho]GSVK.+3y9.heavy	33.3
**644.965855**	896.400475	28.72	sp|P07355|ANXA2_HUMAN.LSLEGDHSTPPSAY[Pho]GSVK.+3y8.heavy	33.3
**644.965855**	910.403113	28.72	sp|P07355|ANXA2_HUMAN.LSLEGDHSTPPSAY[Pho]GSVK.+3y17+2.heavy	33.3
**644.965855**	866.887099	28.72	sp|P07355|ANXA2_HUMAN.LSLEGDHSTPPSAY[Pho]GSVK.+3y16+2.heavy	33.3
**644.965855**	497.230257	28.72	sp|P07355|ANXA2_HUMAN.LSLEGDHSTPPSAY[Pho]GSVK.+3y9+2.heavy	33.3
**715.654712**	830.436657	40.27	sp|P07355|ANXA2_HUMAN.RAEDGSVIDY[Pho]ELIDQDAR.+3y7.light	37
**715.654712**	604.268529	40.27	sp|P07355|ANXA2_HUMAN.RAEDGSVIDY[Pho]ELIDQDAR.+3y5.light	37
**715.654712**	489.241586	40.27	sp|P07355|ANXA2_HUMAN.RAEDGSVIDY[Pho]ELIDQDAR.+3y4.light	37
**715.654712**	361.183009	40.27	sp|P07355|ANXA2_HUMAN.RAEDGSVIDY[Pho]ELIDQDAR.+3y3.light	37
**715.654712**	828.421007	40.27	sp|P07355|ANXA2_HUMAN.RAEDGSVIDY[Pho]ELIDQDAR.+3b8.light	37
**718.990802**	840.444926	40.27	sp|P07355|ANXA2_HUMAN.RAEDGSVIDY[Pho]ELIDQDAR.+3y7.heavy	37
**718.990802**	614.276798	40.27	sp|P07355|ANXA2_HUMAN.RAEDGSVIDY[Pho]ELIDQDAR.+3y5.heavy	37
**718.990802**	499.249855	40.27	sp|P07355|ANXA2_HUMAN.RAEDGSVIDY[Pho]ELIDQDAR.+3y4.heavy	37
**718.990802**	371.191278	40.27	sp|P07355|ANXA2_HUMAN.RAEDGSVIDY[Pho]ELIDQDAR.+3y3.heavy	37
**718.990802**	828.421007	40.27	sp|P07355|ANXA2_HUMAN.RAEDGSVIDY[Pho]ELIDQDAR.+3b8.heavy	37
**603.283747**	778.40938	36.11	sp|P55039|DRG2_HUMAN.Y[Pho]ALVWGTSTK.+2y7.light	32
**603.283747**	679.340966	36.11	sp|P55039|DRG2_HUMAN.Y[Pho]ALVWGTSTK.+2y6.light	32

**603.283747**	493.261653	36.11	sp|P55039|DRG2_HUMAN.Y[Pho]ALVWGTSTK.+2y5.light	32
**603.283747**	315.074049	36.11	sp|P55039|DRG2_HUMAN.Y[Pho]ALVWGTSTK.+2b2.light	32
**603.283747**	428.158113	36.11	sp|P55039|DRG2_HUMAN.Y[Pho]ALVWGTSTK.+2b3.light	32
**607.290846**	786.423579	36.11	sp|P55039|DRG2_HUMAN.Y[Pho]ALVWGTSTK.+2y7.heavy	32
**607.290846**	687.355165	36.11	sp|P55039|DRG2_HUMAN.Y[Pho]ALVWGTSTK.+2y6.heavy	32
**607.290846**	501.275852	36.11	sp|P55039|DRG2_HUMAN.Y[Pho]ALVWGTSTK.+2y5.heavy	32
**607.290846**	315.074049	36.11	sp|P55039|DRG2_HUMAN.Y[Pho]ALVWGTSTK.+2b2.heavy	32
**607.290846**	428.158113	36.11	sp|P55039|DRG2_HUMAN.Y[Pho]ALVWGTSTK.+2b3.heavy	32
**800.694513**	456.292893	35.18	sp|Q15262|PTPRK_HUMAN.YLC[CAM]EGTES[Pho]PYQTGQLHPAIR.+3y4.light	41.3
**800.694513**	1013.475988	35.18	sp|Q15262|PTPRK_HUMAN.YLC[CAM]EGTES[Pho]PYQTGQLHPAIR.+3y18 -98+2.lig	41.3
**800.694513**	917.927815	35.18	sp|Q15262|PTPRK_HUMAN.YLC[CAM]EGTES[Pho]PYQTGQLHPAIR.+3y16+2.light	41.3
**800.694513**	690.872768	35.18	sp|Q15262|PTPRK_HUMAN.YLC[CAM]EGTES[Pho]PYQTGQLHPAIR.+3y12+2.light	41.3
**800.694513**	708.645383	35.18	sp|Q15262|PTPRK_HUMAN.YLC[CAM]EGTES[Pho]PYQTGQLHPAIR.+3y18+3.light	41.3
**804.030603**	466.301162	35.18	sp|Q15262|PTPRK_HUMAN.YLC[CAM]EGTES[Pho]PYQTGQLHPAIR.+3y4.heavy	41.3
**804.030603**	1018.480122	35.18	sp|Q15262|PTPRK_HUMAN.YLC[CAM]EGTES[Pho]PYQTGQLHPAIR.+3y18 -98+2.he	41.3
**804.030603**	922.931949	35.18	sp|Q15262|PTPRK_HUMAN.YLC[CAM]EGTES[Pho]PYQTGQLHPAIR.+3y16+2.heavy	41.3
**804.030603**	695.876902	35.18	sp|Q15262|PTPRK_HUMAN.YLC[CAM]EGTES[Pho]PYQTGQLHPAIR.+3y12+2.heavy	41.3
**804.030603**	711.981472	35.18	sp|Q15262|PTPRK_HUMAN.YLC[CAM]EGTES[Pho]PYQTGQLHPAIR.+3y18+3.heavy	41.3
**1032.099655**	830.463147	30.3	sp|Q08J23|NSUN2_HUMAN.AGEPNS[Pho]PDAEEANSPDVTAGC[CAM]DPAGVHPPR.	53.1
**1032.099655**	823.391196	30.3	sp|Q08J23|NSUN2_HUMAN.AGEPNS[Pho]PDAEEANSPDVTAGC[CAM]DPAGVHPPR.	53.1
**1032.099655**	415.735211	30.3	sp|Q08J23|NSUN2_HUMAN.AGEPNS[Pho]PDAEEANSPDVTAGC[CAM]DPAGVHPPR.	53.1
**1032.099655**	946.399265	30.3	sp|Q08J23|NSUN2_HUMAN.AGEPNS[Pho]PDAEEANSPDVTAGC[CAM]DPAGVHPPR.	53.1
**1032.099655**	914.048344	30.3	sp|Q08J23|NSUN2_HUMAN.AGEPNS[Pho]PDAEEANSPDVTAGC[CAM]DPAGVHPPR.	53.1
**1035.435745**	840.471416	30.3	sp|Q08J23|NSUN2_HUMAN.AGEPNS[Pho]PDAEEANSPDVTAGC[CAM]DPAGVHPPR.	53.1
**1035.435745**	828.39533	30.3	sp|Q08J23|NSUN2_HUMAN.AGEPNS[Pho]PDAEEANSPDVTAGC[CAM]DPAGVHPPR.	53.1
**1035.435745**	420.739346	30.3	sp|Q08J23|NSUN2_HUMAN.AGEPNS[Pho]PDAEEANSPDVTAGC[CAM]DPAGVHPPR.	53.1
**1035.435745**	949.735355	30.3	sp|Q08J23|NSUN2_HUMAN.AGEPNS[Pho]PDAEEANSPDVTAGC[CAM]DPAGVHPPR.	53.1
**1035.435745**	917.384433	30.3	sp|Q08J23|NSUN2_HUMAN.AGEPNS[Pho]PDAEEANSPDVTAGC[CAM]DPAGVHPPR.	53.1
**584.735149**	786.374057	9.7	sp|P51858|HDGF_HUMAN.NST[Pho]PSEPGSGR.+2y8.light	31.2
**584.735149**	473.246672	9.7	sp|P51858|HDGF_HUMAN.NST[Pho]PSEPGSGR.+2y5.light	31.2
**584.735149**	435.209223	9.7	sp|P51858|HDGF_HUMAN.NST[Pho]PSEPGSGR.+2y9 -98+2.light	31.2
**584.735149**	393.690666	9.7	sp|P51858|HDGF_HUMAN.NST[Pho]PSEPGSGR.+2y8+2.light	31.2
**584.735149**	383.096241	9.7	sp|P51858|HDGF_HUMAN.NST[Pho]PSEPGSGR.+2b3.light	31.2
**589.739284**	796.382326	9.7	sp|P51858|HDGF_HUMAN.NST[Pho]PSEPGSGR.+2y8.heavy	31.2
**589.739284**	483.254941	9.7	sp|P51858|HDGF_HUMAN.NST[Pho]PSEPGSGR.+2y5.heavy	31.2
**589.739284**	440.213358	9.7	sp|P51858|HDGF_HUMAN.NST[Pho]PSEPGSGR.+2y9 -98+2.heavy	31.2
**589.739284**	398.694801	9.7	sp|P51858|HDGF_HUMAN.NST[Pho]PSEPGSGR.+2y8+2.heavy	31.2
**589.739284**	383.096241	9.7	sp|P51858|HDGF_HUMAN.NST[Pho]PSEPGSGR.+2b3.heavy	31.2
**533.234254**	966.392818	7.82	sp|O60271|JIP4_HUMAN.VSNS[Pho]PEPQK.+2y8.light	29
**533.234254**	879.360789	7.82	sp|O60271|JIP4_HUMAN.VSNS[Pho]PEPQK.+2y7.light	29
**533.234254**	598.319502	7.82	sp|O60271|JIP4_HUMAN.VSNS[Pho]PEPQK.+2y5.light	29
**533.234254**	372.224145	7.82	sp|O60271|JIP4_HUMAN.VSNS[Pho]PEPQK.+2y3.light	29
**533.234254**	694.244362	7.82	sp|O60271|JIP4_HUMAN.VSNS[Pho]PEPQK.+2b6.light	29
**537.241353**	974.407017	7.82	sp|O60271|JIP4_HUMAN.VSNS[Pho]PEPQK.+2y8.heavy	29
**537.241353**	887.374988	7.82	sp|O60271|JIP4_HUMAN.VSNS[Pho]PEPQK.+2y7.heavy	29
**537.241353**	606.333701	7.82	sp|O60271|JIP4_HUMAN.VSNS[Pho]PEPQK.+2y5.heavy	29
**537.241353**	380.238344	7.82	sp|O60271|JIP4_HUMAN.VSNS[Pho]PEPQK.+2y3.heavy	29
**537.241353**	694.244362	7.82	sp|O60271|JIP4_HUMAN.VSNS[Pho]PEPQK.+2b6.heavy	29
**824.869966**	1107.48303	24.72	sp|Q96C19|EFHD2_HUMAN.ADLNQGIGEPQS[Pho]PSR.+2y10.light	41.8
**824.869966**	937.377502	24.72	sp|Q96C19|EFHD2_HUMAN.ADLNQGIGEPQS[Pho]PSR.+2y8.light	41.8
**824.869966**	751.313445	24.72	sp|Q96C19|EFHD2_HUMAN.ADLNQGIGEPQS[Pho]PSR.+2y6.light	41.8
**824.869966**	653.336549	24.72	sp|Q96C19|EFHD2_HUMAN.ADLNQGIGEPQS[Pho]PSR.+2y6 -98.light	41.8
**824.869966**	359.203744	24.72	sp|Q96C19|EFHD2_HUMAN.ADLNQGIGEPQS[Pho]PSR.+2y3.light	41.8
**829.8741**	1117.491299	24.72	sp|Q96C19|EFHD2_HUMAN.ADLNQGIGEPQS[Pho]PSR.+2y10.heavy	41.8
**829.8741**	947.385771	24.72	sp|Q96C19|EFHD2_HUMAN.ADLNQGIGEPQS[Pho]PSR.+2y8.heavy	41.8
**829.8741**	761.321714	24.72	sp|Q96C19|EFHD2_HUMAN.ADLNQGIGEPQS[Pho]PSR.+2y6.heavy	41.8
**829.8741**	663.344818	24.72	sp|Q96C19|EFHD2_HUMAN.ADLNQGIGEPQS[Pho]PSR.+2y6 -98.heavy	41.8

**829.8741**	369.212013	24.72	sp|Q96C19|EFHD2_HUMAN.ADLNQGIGEPQS[Pho]PSR.+2y3.heavy	41.8
**747.344827**	619.298055	48.6	sp|O43504|LTOR5_HUMAN.NPSIVGVLC[CAM]TDS[Pho]QGLNLGC[CAM]R.+3y5.light	38.6
**747.344827**	392.171064	48.6	sp|O43504|LTOR5_HUMAN.NPSIVGVLC[CAM]TDS[Pho]QGLNLGC[CAM]R.+3y3.light	38.6
**747.344827**	730.786533	48.6	sp|O43504|LTOR5_HUMAN.NPSIVGVLC[CAM]TDS[Pho]QGLNLGC[CAM]R.+3y12+2.	38.6
**747.344827**	709.330518	48.6	sp|O43504|LTOR5_HUMAN.NPSIVGVLC[CAM]TDS[Pho]QGLNLGC[CAM]R.+3y19+3.	38.6
**747.344827**	667.377351	48.6	sp|O43504|LTOR5_HUMAN.NPSIVGVLC[CAM]TDS[Pho]QGLNLGC[CAM]R.+3b7.ligh	38.6
**750.680917**	629.306324	48.6	sp|O43504|LTOR5_HUMAN.NPSIVGVLC[CAM]TDS[Pho]QGLNLGC[CAM]R.+3y5.hea	38.6
**750.680917**	402.179333	48.6	sp|O43504|LTOR5_HUMAN.NPSIVGVLC[CAM]TDS[Pho]QGLNLGC[CAM]R.+3y3.hea	38.6
**750.680917**	735.790668	48.6	sp|O43504|LTOR5_HUMAN.NPSIVGVLC[CAM]TDS[Pho]QGLNLGC[CAM]R.+3y12+2.	38.6
**750.680917**	712.666608	48.6	sp|O43504|LTOR5_HUMAN.NPSIVGVLC[CAM]TDS[Pho]QGLNLGC[CAM]R.+3y19+3.	38.6
**750.680917**	667.377351	48.6	sp|O43504|LTOR5_HUMAN.NPSIVGVLC[CAM]TDS[Pho]QGLNLGC[CAM]R.+3b7.hea	38.6
**673.766646**	827.329489	23.22	sp|Q96MG7|MAGG1_HUMAN.DGFAEEAPSTS[Pho]R.+2y7.light	35.1
**673.766646**	698.286896	23.22	sp|Q96MG7|MAGG1_HUMAN.DGFAEEAPSTS[Pho]R.+2y6.light	35.1
**673.766646**	627.249782	23.22	sp|Q96MG7|MAGG1_HUMAN.DGFAEEAPSTS[Pho]R.+2y5.light	35.1
**673.766646**	529.272886	23.22	sp|Q96MG7|MAGG1_HUMAN.DGFAEEAPSTS[Pho]R.+2y5 -98.light	35.1
**673.766646**	720.283511	23.22	sp|Q96MG7|MAGG1_HUMAN.DGFAEEAPSTS[Pho]R.+2b7.light	35.1
**678.770781**	837.337758	23.22	sp|Q96MG7|MAGG1_HUMAN.DGFAEEAPSTS[Pho]R.+2y7.heavy	35.1
**678.770781**	708.295165	23.22	sp|Q96MG7|MAGG1_HUMAN.DGFAEEAPSTS[Pho]R.+2y6.heavy	35.1
**678.770781**	637.258051	23.22	sp|Q96MG7|MAGG1_HUMAN.DGFAEEAPSTS[Pho]R.+2y5.heavy	35.1
**678.770781**	539.281155	23.22	sp|Q96MG7|MAGG1_HUMAN.DGFAEEAPSTS[Pho]R.+2y5 -98.heavy	35.1
**678.770781**	720.283511	23.22	sp|Q96MG7|MAGG1_HUMAN.DGFAEEAPSTS[Pho]R.+2b7.heavy	35.1
**498.538731**	633.283845	12.5	sp|Q96RL1|UIMC1_HUMAN.GSHIS[Pho]QGNEAEER.+3y5.light	25.9
**498.538731**	504.241252	12.5	sp|Q96RL1|UIMC1_HUMAN.GSHIS[Pho]QGNEAEER.+3y4.light	25.9
**498.538731**	433.204138	12.5	sp|Q96RL1|UIMC1_HUMAN.GSHIS[Pho]QGNEAEER.+3y3.light	25.9
**498.538731**	304.161545	12.5	sp|Q96RL1|UIMC1_HUMAN.GSHIS[Pho]QGNEAEER.+3y2.light	25.9
**498.538731**	382.177726	12.5	sp|Q96RL1|UIMC1_HUMAN.GSHIS[Pho]QGNEAEER.+3b8 -98+2.light	25.9
**501.874821**	643.292114	12.5	sp|Q96RL1|UIMC1_HUMAN.GSHIS[Pho]QGNEAEER.+3y5.heavy	25.9
**501.874821**	514.249521	12.5	sp|Q96RL1|UIMC1_HUMAN.GSHIS[Pho]QGNEAEER.+3y4.heavy	25.9
**501.874821**	443.212407	12.5	sp|Q96RL1|UIMC1_HUMAN.GSHIS[Pho]QGNEAEER.+3y3.heavy	25.9
**501.874821**	314.169814	12.5	sp|Q96RL1|UIMC1_HUMAN.GSHIS[Pho]QGNEAEER.+3y2.heavy	25.9
**501.874821**	382.177726	12.5	sp|Q96RL1|UIMC1_HUMAN.GSHIS[Pho]QGNEAEER.+3b8 -98+2.heavy	25.9
**702.324997**	401.214309	18.2	sp|Q92993|KAT5_HUMAN.NGLPGSRPGS[Pho]PER.+2y3.light	36.4
**702.324997**	645.303533	18.2	sp|Q92993|KAT5_HUMAN.NGLPGSRPGS[Pho]PER.+2y12+2.light	36.4
**702.324997**	560.250769	18.2	sp|Q92993|KAT5_HUMAN.NGLPGSRPGS[Pho]PER.+2y10+2.light	36.4
**702.324997**	682.363098	18.2	sp|Q92993|KAT5_HUMAN.NGLPGSRPGS[Pho]PER.+2b7.light	36.4
**702.324997**	905.458789	18.2	sp|Q92993|KAT5_HUMAN.NGLPGSRPGS[Pho]PER.+2b10 -98.light	36.4
**707.329132**	411.222578	18.2	sp|Q92993|KAT5_HUMAN.NGLPGSRPGS[Pho]PER.+2y3.heavy	36.4
**707.329132**	650.307668	18.2	sp|Q92993|KAT5_HUMAN.NGLPGSRPGS[Pho]PER.+2y12+2.heavy	36.4
**707.329132**	565.254904	18.2	sp|Q92993|KAT5_HUMAN.NGLPGSRPGS[Pho]PER.+2y10+2.heavy	36.4
**707.329132**	682.363098	18.2	sp|Q92993|KAT5_HUMAN.NGLPGSRPGS[Pho]PER.+2b7.heavy	36.4
**707.329132**	905.458789	18.2	sp|Q92993|KAT5_HUMAN.NGLPGSRPGS[Pho]PER.+2b10 -98.heavy	36.4
**997.441149**	1213.569522	40.73	sp|P04626|ERBB2_HUMAN.S[Pho]GGGDLTLGLEPSEEEAPR.+2y11.light	49.4
**997.441149**	1156.548058	40.73	sp|P04626|ERBB2_HUMAN.S[Pho]GGGDLTLGLEPSEEEAPR.+2y10.light	49.4
**997.441149**	1043.463994	40.73	sp|P04626|ERBB2_HUMAN.S[Pho]GGGDLTLGLEPSEEEAPR.+2y9.light	49.4
**997.441149**	914.421401	40.73	sp|P04626|ERBB2_HUMAN.S[Pho]GGGDLTLGLEPSEEEAPR.+2y8.light	49.4
**997.441149**	1080.460897	40.73	sp|P04626|ERBB2_HUMAN.S[Pho]GGGDLTLGLEPSEEEAPR.+2b11.light	49.4
**1002.445284**	1223.577791	40.73	sp|P04626|ERBB2_HUMAN.S[Pho]GGGDLTLGLEPSEEEAPR.+2y11.heavy	49.4
**1002.445284**	1166.556327	40.73	sp|P04626|ERBB2_HUMAN.S[Pho]GGGDLTLGLEPSEEEAPR.+2y10.heavy	49.4
**1002.445284**	1053.472263	40.73	sp|P04626|ERBB2_HUMAN.S[Pho]GGGDLTLGLEPSEEEAPR.+2y9.heavy	49.4
**1002.445284**	924.42967	40.73	sp|P04626|ERBB2_HUMAN.S[Pho]GGGDLTLGLEPSEEEAPR.+2y8.heavy	49.4
**1002.445284**	1080.460897	40.73	sp|P04626|ERBB2_HUMAN.S[Pho]GGGDLTLGLEPSEEEAPR.+2b11.heavy	49.4
**502.749874**	692.312717	17.8	sp|Q9NQS7|INCE_HUMAN.IAQVS[Pho]PGPR.+2y6.light	27.6
**502.749874**	594.335821	17.8	sp|Q9NQS7|INCE_HUMAN.IAQVS[Pho]PGPR.+2y6 -98.light	27.6
**502.749874**	593.244303	17.8	sp|Q9NQS7|INCE_HUMAN.IAQVS[Pho]PGPR.+2y5.light	27.6
**502.749874**	426.245943	17.8	sp|Q9NQS7|INCE_HUMAN.IAQVS[Pho]PGPR.+2y4.light	27.6
**502.749874**	313.187031	17.8	sp|Q9NQS7|INCE_HUMAN.IAQVS[Pho]PGPR.+2b3.light	27.6
**507.754008**	702.320986	17.8	sp|Q9NQS7|INCE_HUMAN.IAQVS[Pho]PGPR.+2y6.heavy	27.6

**507.754008**	604.34409	17.8	sp|Q9NQS7|INCE_HUMAN.IAQVS[Pho]PGPR.+2y6 -98.heavy	27.6
**507.754008**	603.252572	17.8	sp|Q9NQS7|INCE_HUMAN.IAQVS[Pho]PGPR.+2y5.heavy	27.6
**507.754008**	436.254212	17.8	sp|Q9NQS7|INCE_HUMAN.IAQVS[Pho]PGPR.+2y4.heavy	27.6
**507.754008**	313.187031	17.8	sp|Q9NQS7|INCE_HUMAN.IAQVS[Pho]PGPR.+2b3.heavy	27.6
**688.31069**	872.427746	37.37	sp|Q9H0E9|BRD8_HUMAN.EESGTIFGS[Pho]QIK.+2y7.light	35.8
**688.31069**	759.343682	37.37	sp|Q9H0E9|BRD8_HUMAN.EESGTIFGS[Pho]QIK.+2y6.light	35.8
**688.31069**	661.366786	37.37	sp|Q9H0E9|BRD8_HUMAN.EESGTIFGS[Pho]QIK.+2y6 -98.light	35.8
**688.31069**	612.275268	37.37	sp|Q9H0E9|BRD8_HUMAN.EESGTIFGS[Pho]QIK.+2y5.light	35.8
**688.31069**	514.298372	37.37	sp|Q9H0E9|BRD8_HUMAN.EESGTIFGS[Pho]QIK.+2y5 -98.light	35.8
**692.317789**	880.441945	37.37	sp|Q9H0E9|BRD8_HUMAN.EESGTIFGS[Pho]QIK.+2y7.heavy	35.8
**692.317789**	767.357881	37.37	sp|Q9H0E9|BRD8_HUMAN.EESGTIFGS[Pho]QIK.+2y6.heavy	35.8
**692.317789**	669.380985	37.37	sp|Q9H0E9|BRD8_HUMAN.EESGTIFGS[Pho]QIK.+2y6 -98.heavy	35.8
**692.317789**	620.289467	37.37	sp|Q9H0E9|BRD8_HUMAN.EESGTIFGS[Pho]QIK.+2y5.heavy	35.8
**692.317789**	522.312571	37.37	sp|Q9H0E9|BRD8_HUMAN.EESGTIFGS[Pho]QIK.+2y5 -98.heavy	35.8
**787.348335**	1112.46196	33.56	sp|Q9H910|HN1L_HUMAN.GSGIFDEST[Pho]PVQTR.+2y9.light	40.1
**787.348335**	868.392424	33.56	sp|Q9H910|HN1L_HUMAN.GSGIFDEST[Pho]PVQTR.+2y7.light	40.1
**787.348335**	683.383499	33.56	sp|Q9H910|HN1L_HUMAN.GSGIFDEST[Pho]PVQTR.+2y6 -98.light	40.1
**787.348335**	600.346386	33.56	sp|Q9H910|HN1L_HUMAN.GSGIFDEST[Pho]PVQTR.+2y5.light	40.1
**787.348335**	404.225208	33.56	sp|Q9H910|HN1L_HUMAN.GSGIFDEST[Pho]PVQTR.+2y3.light	40.1
**792.352469**	1122.470229	33.56	sp|Q9H910|HN1L_HUMAN.GSGIFDEST[Pho]PVQTR.+2y9.heavy	40.1
**792.352469**	878.400693	33.56	sp|Q9H910|HN1L_HUMAN.GSGIFDEST[Pho]PVQTR.+2y7.heavy	40.1
**792.352469**	693.391768	33.56	sp|Q9H910|HN1L_HUMAN.GSGIFDEST[Pho]PVQTR.+2y6 -98.heavy	40.1
**792.352469**	610.354655	33.56	sp|Q9H910|HN1L_HUMAN.GSGIFDEST[Pho]PVQTR.+2y5.heavy	40.1
**792.352469**	414.233477	33.56	sp|Q9H910|HN1L_HUMAN.GSGIFDEST[Pho]PVQTR.+2y3.heavy	40.1
**991.439961**	1076.431314	50.8	sp|P51587|BRCA2_HUMAN.LQLFIT[Pho]PEADSLSC[CAM]LQEGQC[CAM]ENDPK.+3	51.1
**991.439961**	947.388721	50.8	sp|P51587|BRCA2_HUMAN.LQLFIT[Pho]PEADSLSC[CAM]LQEGQC[CAM]ENDPK.+3	51.1
**991.439961**	762.30868	50.8	sp|P51587|BRCA2_HUMAN.LQLFIT[Pho]PEADSLSC[CAM]LQEGQC[CAM]ENDPK.+3	51.1
**991.439961**	953.745273	50.8	sp|P51587|BRCA2_HUMAN.LQLFIT[Pho]PEADSLSC[CAM]LQEGQC[CAM]ENDPK.+3	51.1
**991.439961**	615.38646	50.8	sp|P51587|BRCA2_HUMAN.LQLFIT[Pho]PEADSLSC[CAM]LQEGQC[CAM]ENDPK.+3	51.1
**994.111361**	1084.445513	50.8	sp|P51587|BRCA2_HUMAN.LQLFIT[Pho]PEADSLSC[CAM]LQEGQC[CAM]ENDPK.+3	51.1
**994.111361**	955.40292	50.8	sp|P51587|BRCA2_HUMAN.LQLFIT[Pho]PEADSLSC[CAM]LQEGQC[CAM]ENDPK.+3	51.1
**994.111361**	770.322879	50.8	sp|P51587|BRCA2_HUMAN.LQLFIT[Pho]PEADSLSC[CAM]LQEGQC[CAM]ENDPK.+3	51.1
**994.111361**	956.416673	50.8	sp|P51587|BRCA2_HUMAN.LQLFIT[Pho]PEADSLSC[CAM]LQEGQC[CAM]ENDPK.+3	51.1
**994.111361**	615.38646	50.8	sp|P51587|BRCA2_HUMAN.LQLFIT[Pho]PEADSLSC[CAM]LQEGQC[CAM]ENDPK.+3	51.1
**801.418563**	994.593158	41.81	sp|P0C1Z6|TFPT_HUMAN.AGNALT[Pho]PELAPVQIK.+2y9.light	40.8
**801.418563**	655.413737	41.81	sp|P0C1Z6|TFPT_HUMAN.AGNALT[Pho]PELAPVQIK.+2y6.light	40.8
**801.418563**	584.376623	41.81	sp|P0C1Z6|TFPT_HUMAN.AGNALT[Pho]PELAPVQIK.+2y5.light	40.8
**801.418563**	510.267072	41.81	sp|P0C1Z6|TFPT_HUMAN.AGNALT[Pho]PELAPVQIK.+2b6 -98.light	40.8
**801.418563**	920.483607	41.81	sp|P0C1Z6|TFPT_HUMAN.AGNALT[Pho]PELAPVQIK.+2b10 -98.light	40.8
**805.425663**	1002.607357	41.81	sp|P0C1Z6|TFPT_HUMAN.AGNALT[Pho]PELAPVQIK.+2y9.heavy	40.8
**805.425663**	663.427936	41.81	sp|P0C1Z6|TFPT_HUMAN.AGNALT[Pho]PELAPVQIK.+2y6.heavy	40.8
**805.425663**	592.390822	41.81	sp|P0C1Z6|TFPT_HUMAN.AGNALT[Pho]PELAPVQIK.+2y5.heavy	40.8
**805.425663**	510.267072	41.81	sp|P0C1Z6|TFPT_HUMAN.AGNALT[Pho]PELAPVQIK.+2b6 -98.heavy	40.8
**805.425663**	920.483607	41.81	sp|P0C1Z6|TFPT_HUMAN.AGNALT[Pho]PELAPVQIK.+2b10 -98.heavy	40.8
**707.949242**	1186.430677	34.45	sp|P08581|MET_HUMAN.ATFPEDQFPNSS[Pho]QNGSC[CAM]R.+3y10.light	36.6
**707.949242**	593.24602	34.45	sp|P08581|MET_HUMAN.ATFPEDQFPNSS[Pho]QNGSC[CAM]R.+3y5.light	36.6
**707.949242**	479.203092	34.45	sp|P08581|MET_HUMAN.ATFPEDQFPNSS[Pho]QNGSC[CAM]R.+3y4.light	36.6
**707.949242**	667.253183	34.45	sp|P08581|MET_HUMAN.ATFPEDQFPNSS[Pho]QNGSC[CAM]R.+3y11+2.light	36.6
**707.949242**	593.718976	34.45	sp|P08581|MET_HUMAN.ATFPEDQFPNSS[Pho]QNGSC[CAM]R.+3y10+2.light	36.6
**711.285332**	1196.438946	34.45	sp|P08581|MET_HUMAN.ATFPEDQFPNSS[Pho]QNGSC[CAM]R.+3y10.heavy	36.6
**711.285332**	603.254289	34.45	sp|P08581|MET_HUMAN.ATFPEDQFPNSS[Pho]QNGSC[CAM]R.+3y5.heavy	36.6
**711.285332**	489.211361	34.45	sp|P08581|MET_HUMAN.ATFPEDQFPNSS[Pho]QNGSC[CAM]R.+3y4.heavy	36.6
**711.285332**	672.257318	34.45	sp|P08581|MET_HUMAN.ATFPEDQFPNSS[Pho]QNGSC[CAM]R.+3y11+2.heavy	36.6
**711.285332**	598.723111	34.45	sp|P08581|MET_HUMAN.ATFPEDQFPNSS[Pho]QNGSC[CAM]R.+3y10+2.heavy	36.6
**591.239294**	827.344745	23.9	sp|P08581|MET_HUMAN.DMYDKEYY[Pho]SVHNK.+3y6.light	30.7
**591.239294**	398.214643	23.9	sp|P08581|MET_HUMAN.DMYDKEYY[Pho]SVHNK.+3y3.light	30.7
**591.239294**	763.321589	23.9	sp|P08581|MET_HUMAN.DMYDKEYY[Pho]SVHNK.+3y11+2.light	30.7

**591.239294**	681.789924	23.9	sp|P08581|MET_HUMAN.DMYDKEYY[Pho]SVHNK.+3y10+2.light	30.7
**591.239294**	624.276453	23.9	sp|P08581|MET_HUMAN.DMYDKEYY[Pho]SVHNK.+3y9+2.light	30.7
**593.910693**	835.358944	23.9	sp|P08581|MET_HUMAN.DMYDKEYY[Pho]SVHNK.+3y6.heavy	30.7
**593.910693**	406.228842	23.9	sp|P08581|MET_HUMAN.DMYDKEYY[Pho]SVHNK.+3y3.heavy	30.7
**593.910693**	767.328688	23.9	sp|P08581|MET_HUMAN.DMYDKEYY[Pho]SVHNK.+3y11+2.heavy	30.7
**593.910693**	685.797024	23.9	sp|P08581|MET_HUMAN.DMYDKEYY[Pho]SVHNK.+3y10+2.heavy	30.7
**593.910693**	628.283552	23.9	sp|P08581|MET_HUMAN.DMYDKEYY[Pho]SVHNK.+3y9+2.heavy	30.7
**617.894737**	827.344745	27.53	sp|P08581|MET_HUMAN.DMYDKEY[Pho]Y[Pho]SVHNK.+3y6.light	32
**617.894737**	584.315085	27.53	sp|P08581|MET_HUMAN.DMYDKEY[Pho]Y[Pho]SVHNK.+3y5.light	32
**617.894737**	398.214643	27.53	sp|P08581|MET_HUMAN.DMYDKEY[Pho]Y[Pho]SVHNK.+3y3.light	32
**617.894737**	721.77309	27.53	sp|P08581|MET_HUMAN.DMYDKEY[Pho]Y[Pho]SVHNK.+3y10+2.light	32
**617.894737**	664.259618	27.53	sp|P08581|MET_HUMAN.DMYDKEY[Pho]Y[Pho]SVHNK.+3y9+2.light	32
**620.566137**	835.358944	27.53	sp|P08581|MET_HUMAN.DMYDKEY[Pho]Y[Pho]SVHNK.+3y6.heavy	32
**620.566137**	592.329284	27.53	sp|P08581|MET_HUMAN.DMYDKEY[Pho]Y[Pho]SVHNK.+3y5.heavy	32
**620.566137**	406.228842	27.53	sp|P08581|MET_HUMAN.DMYDKEY[Pho]Y[Pho]SVHNK.+3y3.heavy	32
**620.566137**	725.780189	27.53	sp|P08581|MET_HUMAN.DMYDKEY[Pho]Y[Pho]SVHNK.+3y10+2.heavy	32
**620.566137**	668.266718	27.53	sp|P08581|MET_HUMAN.DMYDKEY[Pho]Y[Pho]SVHNK.+3y9+2.heavy	32
**752.788641**	1000.336759	14.3	sp|Q14839|CHD4_HUMAN.QVNYNDGS[Pho]QEDR.+2y8.light	38.6
**752.788641**	886.293832	14.3	sp|Q14839|CHD4_HUMAN.QVNYNDGS[Pho]QEDR.+2y7.light	38.6
**752.788641**	771.266889	14.3	sp|Q14839|CHD4_HUMAN.QVNYNDGS[Pho]QEDR.+2y6.light	38.6
**752.788641**	673.289993	14.3	sp|Q14839|CHD4_HUMAN.QVNYNDGS[Pho]QEDR.+2y6 -98.light	38.6
**752.788641**	639.225146	14.3	sp|Q14839|CHD4_HUMAN.QVNYNDGS[Pho]QEDR.+2y10+2.light	38.6
**757.792776**	1010.345028	14.3	sp|Q14839|CHD4_HUMAN.QVNYNDGS[Pho]QEDR.+2y8.heavy	38.6
**757.792776**	896.302101	14.3	sp|Q14839|CHD4_HUMAN.QVNYNDGS[Pho]QEDR.+2y7.heavy	38.6
**757.792776**	781.275158	14.3	sp|Q14839|CHD4_HUMAN.QVNYNDGS[Pho]QEDR.+2y6.heavy	38.6
**757.792776**	683.298262	14.3	sp|Q14839|CHD4_HUMAN.QVNYNDGS[Pho]QEDR.+2y6 -98.heavy	38.6
**757.792776**	644.22928	14.3	sp|Q14839|CHD4_HUMAN.QVNYNDGS[Pho]QEDR.+2y10+2.heavy	38.6
**543.250299**	743.393395	24.5	sp|Q9UHB6|LIMA1_HUMAN.ETPHS[Pho]PGVEDAPIAK.+3y7.light	28.2
**543.250299**	614.350802	24.5	sp|Q9UHB6|LIMA1_HUMAN.ETPHS[Pho]PGVEDAPIAK.+3y6.light	28.2
**543.250299**	499.323859	24.5	sp|Q9UHB6|LIMA1_HUMAN.ETPHS[Pho]PGVEDAPIAK.+3y5.light	28.2
**543.250299**	428.286745	24.5	sp|Q9UHB6|LIMA1_HUMAN.ETPHS[Pho]PGVEDAPIAK.+3y4.light	28.2
**543.250299**	699.326674	24.5	sp|Q9UHB6|LIMA1_HUMAN.ETPHS[Pho]PGVEDAPIAK.+3y13+2.light	28.2
**545.921698**	751.407594	24.5	sp|Q9UHB6|LIMA1_HUMAN.ETPHS[Pho]PGVEDAPIAK.+3y7.heavy	28.2
**545.921698**	622.365001	24.5	sp|Q9UHB6|LIMA1_HUMAN.ETPHS[Pho]PGVEDAPIAK.+3y6.heavy	28.2
**545.921698**	507.338058	24.5	sp|Q9UHB6|LIMA1_HUMAN.ETPHS[Pho]PGVEDAPIAK.+3y5.heavy	28.2
**545.921698**	436.300944	24.5	sp|Q9UHB6|LIMA1_HUMAN.ETPHS[Pho]PGVEDAPIAK.+3y4.heavy	28.2
**545.921698**	703.333774	24.5	sp|Q9UHB6|LIMA1_HUMAN.ETPHS[Pho]PGVEDAPIAK.+3y13+2.heavy	28.2
**527.731878**	724.302546	4.82	sp|P06748|NPM_HUMAN.DSKPSST[Pho]PR.+2y6.light	28.7
**527.731878**	627.249782	4.82	sp|P06748|NPM_HUMAN.DSKPSST[Pho]PR.+2y5.light	28.7
**527.731878**	540.217754	4.82	sp|P06748|NPM_HUMAN.DSKPSST[Pho]PR.+2y4.light	28.7
**527.731878**	362.654911	4.82	sp|P06748|NPM_HUMAN.DSKPSST[Pho]PR.+2y6+2.light	28.7
**527.731878**	331.161211	4.82	sp|P06748|NPM_HUMAN.DSKPSST[Pho]PR.+2b3.light	28.7
**532.736013**	734.310815	4.82	sp|P06748|NPM_HUMAN.DSKPSST[Pho]PR.+2y6.heavy	28.7
**532.736013**	637.258051	4.82	sp|P06748|NPM_HUMAN.DSKPSST[Pho]PR.+2y5.heavy	28.7
**532.736013**	550.226023	4.82	sp|P06748|NPM_HUMAN.DSKPSST[Pho]PR.+2y4.heavy	28.7
**532.736013**	367.659045	4.82	sp|P06748|NPM_HUMAN.DSKPSST[Pho]PR.+2y6+2.heavy	28.7
**532.736013**	331.161211	4.82	sp|P06748|NPM_HUMAN.DSKPSST[Pho]PR.+2b3.heavy	28.7
**601.315047**	817.421933	36.12	sp|Q04637|IF4G1_HUMAN.EAALPPVS[Pho]PLK.+2y7.light	32
**601.315047**	720.369169	36.12	sp|Q04637|IF4G1_HUMAN.EAALPPVS[Pho]PLK.+2y6.light	32
**601.315047**	409.214604	36.12	sp|Q04637|IF4G1_HUMAN.EAALPPVS[Pho]PLK.+2y7+2.light	32
**601.315047**	360.688222	36.12	sp|Q04637|IF4G1_HUMAN.EAALPPVS[Pho]PLK.+2y6+2.light	32
**601.315047**	385.208161	36.12	sp|Q04637|IF4G1_HUMAN.EAALPPVS[Pho]PLK.+2b4.light	32
**605.322146**	825.436132	36.12	sp|Q04637|IF4G1_HUMAN.EAALPPVS[Pho]PLK.+2y7.heavy	32
**605.322146**	728.383368	36.12	sp|Q04637|IF4G1_HUMAN.EAALPPVS[Pho]PLK.+2y6.heavy	32
**605.322146**	413.221704	36.12	sp|Q04637|IF4G1_HUMAN.EAALPPVS[Pho]PLK.+2y7+2.heavy	32
**605.322146**	364.695322	36.12	sp|Q04637|IF4G1_HUMAN.EAALPPVS[Pho]PLK.+2y6+2.heavy	32
**605.322146**	385.208161	36.12	sp|Q04637|IF4G1_HUMAN.EAALPPVS[Pho]PLK.+2b4.heavy	32

**531.218604**	752.357344	6.97	sp|Q92796|DLG3_HUMAN.QT[Pho]YEQANK.+2y6.light	28.9
**531.218604**	589.294016	6.97	sp|Q92796|DLG3_HUMAN.QT[Pho]YEQANK.+2y5.light	28.9
**531.218604**	460.251423	6.97	sp|Q92796|DLG3_HUMAN.QT[Pho]YEQANK.+2y4.light	28.9
**531.218604**	332.192845	6.97	sp|Q92796|DLG3_HUMAN.QT[Pho]YEQANK.+2y3.light	28.9
**531.218604**	310.079863	6.97	sp|Q92796|DLG3_HUMAN.QT[Pho]YEQANK.+2b2.light	28.9
**535.225703**	760.371543	6.97	sp|Q92796|DLG3_HUMAN.QT[Pho]YEQANK.+2y6.heavy	28.9
**535.225703**	597.308215	6.97	sp|Q92796|DLG3_HUMAN.QT[Pho]YEQANK.+2y5.heavy	28.9
**535.225703**	468.265622	6.97	sp|Q92796|DLG3_HUMAN.QT[Pho]YEQANK.+2y4.heavy	28.9
**535.225703**	340.207044	6.97	sp|Q92796|DLG3_HUMAN.QT[Pho]YEQANK.+2y3.heavy	28.9
**535.225703**	310.079863	6.97	sp|Q92796|DLG3_HUMAN.QT[Pho]YEQANK.+2b2.heavy	28.9
**452.694233**	661.351531	7.73	sp|O14737|PDCD5_HUMAN.Y[Pho]GQLSEK.+2y6.light	25.4
**452.694233**	476.271489	7.73	sp|O14737|PDCD5_HUMAN.Y[Pho]GQLSEK.+2y4.light	25.4
**452.694233**	363.187425	7.73	sp|O14737|PDCD5_HUMAN.Y[Pho]GQLSEK.+2y3.light	25.4
**452.694233**	301.058399	7.73	sp|O14737|PDCD5_HUMAN.Y[Pho]GQLSEK.+2b2.light	25.4
**452.694233**	429.116977	7.73	sp|O14737|PDCD5_HUMAN.Y[Pho]GQLSEK.+2b3.light	25.4
**456.701333**	669.36573	7.73	sp|O14737|PDCD5_HUMAN.Y[Pho]GQLSEK.+2y6.heavy	25.4
**456.701333**	484.285688	7.73	sp|O14737|PDCD5_HUMAN.Y[Pho]GQLSEK.+2y4.heavy	25.4
**456.701333**	371.201624	7.73	sp|O14737|PDCD5_HUMAN.Y[Pho]GQLSEK.+2y3.heavy	25.4
**456.701333**	301.058399	7.73	sp|O14737|PDCD5_HUMAN.Y[Pho]GQLSEK.+2b2.heavy	25.4
**456.701333**	429.116977	7.73	sp|O14737|PDCD5_HUMAN.Y[Pho]GQLSEK.+2b3.heavy	25.4
**613.308482**	806.885222	30.39	sp|Q6PL18|ATAD2_HUMAN.KPNIFYSGPAS[Pho]PARPR.+3y14+2.light	31.8
**613.308482**	757.896774	30.39	sp|Q6PL18|ATAD2_HUMAN.KPNIFYSGPAS[Pho]PARPR.+3y14 -98+2.light	31.8
**613.308482**	644.333278	30.39	sp|Q6PL18|ATAD2_HUMAN.KPNIFYSGPAS[Pho]PARPR.+3y12 -98+2.light	31.8
**613.308482**	538.25924	30.39	sp|Q6PL18|ATAD2_HUMAN.KPNIFYSGPAS[Pho]PARPR.+3y14+3.light	31.8
**613.308482**	783.876865	30.39	sp|Q6PL18|ATAD2_HUMAN.KPNIFYSGPAS[Pho]PARPR.+3b14+2.light	31.8
**616.644572**	811.889356	30.39	sp|Q6PL18|ATAD2_HUMAN.KPNIFYSGPAS[Pho]PARPR.+3y14+2.heavy	31.8
**616.644572**	762.900908	30.39	sp|Q6PL18|ATAD2_HUMAN.KPNIFYSGPAS[Pho]PARPR.+3y14 -98+2.heavy	31.8
**616.644572**	649.337413	30.39	sp|Q6PL18|ATAD2_HUMAN.KPNIFYSGPAS[Pho]PARPR.+3y12 -98+2.heavy	31.8
**616.644572**	541.59533	30.39	sp|Q6PL18|ATAD2_HUMAN.KPNIFYSGPAS[Pho]PARPR.+3y14+3.heavy	31.8
**616.644572**	783.876865	30.39	sp|Q6PL18|ATAD2_HUMAN.KPNIFYSGPAS[Pho]PARPR.+3b14+2.heavy	31.8
**746.329414**	1179.471796	34.24	sp|Q96RT1|LAP2_HUMAN.AQIPEGDY[Pho]LSYR.+2y9.light	38.3
**746.329414**	953.376439	34.24	sp|Q96RT1|LAP2_HUMAN.AQIPEGDY[Pho]LSYR.+2y7.light	38.3
**746.329414**	646.781568	34.24	sp|Q96RT1|LAP2_HUMAN.AQIPEGDY[Pho]LSYR.+2y10+2.light	38.3
**746.329414**	590.239536	34.24	sp|Q96RT1|LAP2_HUMAN.AQIPEGDY[Pho]LSYR.+2y9+2.light	38.3
**746.329414**	313.187031	34.24	sp|Q96RT1|LAP2_HUMAN.AQIPEGDY[Pho]LSYR.+2b3.light	38.3
**751.333548**	1189.480065	34.24	sp|Q96RT1|LAP2_HUMAN.AQIPEGDY[Pho]LSYR.+2y9.heavy	38.3
**751.333548**	963.384708	34.24	sp|Q96RT1|LAP2_HUMAN.AQIPEGDY[Pho]LSYR.+2y7.heavy	38.3
**751.333548**	651.785703	34.24	sp|Q96RT1|LAP2_HUMAN.AQIPEGDY[Pho]LSYR.+2y10+2.heavy	38.3
**751.333548**	595.243671	34.24	sp|Q96RT1|LAP2_HUMAN.AQIPEGDY[Pho]LSYR.+2y9+2.heavy	38.3
**751.333548**	313.187031	34.24	sp|Q96RT1|LAP2_HUMAN.AQIPEGDY[Pho]LSYR.+2b3.heavy	38.3
**803.039003**	781.49305	39.92	sp|Q12888|TP53B_HUMAN.SGTAETEPVEQDSS[Pho]QPSLPLVR.+3y7.light	41.5
**803.039003**	684.440286	39.92	sp|Q12888|TP53B_HUMAN.SGTAETEPVEQDSS[Pho]QPSLPLVR.+3y6.light	41.5
**803.039003**	597.408258	39.92	sp|Q12888|TP53B_HUMAN.SGTAETEPVEQDSS[Pho]QPSLPLVR.+3y5.light	41.5
**803.039003**	484.324194	39.92	sp|Q12888|TP53B_HUMAN.SGTAETEPVEQDSS[Pho]QPSLPLVR.+3y4.light	41.5
**803.039003**	391.250163	39.92	sp|Q12888|TP53B_HUMAN.SGTAETEPVEQDSS[Pho]QPSLPLVR.+3y7+2.light	41.5
**806.375092**	791.501319	39.92	sp|Q12888|TP53B_HUMAN.SGTAETEPVEQDSS[Pho]QPSLPLVR.+3y7.heavy	41.5
**806.375092**	694.448555	39.92	sp|Q12888|TP53B_HUMAN.SGTAETEPVEQDSS[Pho]QPSLPLVR.+3y6.heavy	41.5
**806.375092**	607.416527	39.92	sp|Q12888|TP53B_HUMAN.SGTAETEPVEQDSS[Pho]QPSLPLVR.+3y5.heavy	41.5
**806.375092**	494.332463	39.92	sp|Q12888|TP53B_HUMAN.SGTAETEPVEQDSS[Pho]QPSLPLVR.+3y4.heavy	41.5
**806.375092**	396.254297	39.92	sp|Q12888|TP53B_HUMAN.SGTAETEPVEQDSS[Pho]QPSLPLVR.+3y7+2.heavy	41.5
**832.879999**	810.339325	13.6	sp|Q12888|TP53B_HUMAN.LDQELQQPQT[Pho]QEK.+2y6.light	42.1
**832.879999**	357.176861	13.6	sp|Q12888|TP53B_HUMAN.LDQELQQPQT[Pho]QEK.+2b3.light	42.1
**832.879999**	486.219454	13.6	sp|Q12888|TP53B_HUMAN.LDQELQQPQT[Pho]QEK.+2b4.light	42.1
**832.879999**	599.303518	13.6	sp|Q12888|TP53B_HUMAN.LDQELQQPQT[Pho]QEK.+2b5.light	42.1
**832.879999**	855.420673	13.6	sp|Q12888|TP53B_HUMAN.LDQELQQPQT[Pho]QEK.+2b7.light	42.1
**836.887099**	818.353524	13.6	sp|Q12888|TP53B_HUMAN.LDQELQQPQT[Pho]QEK.+2y6.heavy	42.1
**836.887099**	357.176861	13.6	sp|Q12888|TP53B_HUMAN.LDQELQQPQT[Pho]QEK.+2b3.heavy	42.1

**836.887099**	486.219454	13.6	sp|Q12888|TP53B_HUMAN.LDQELQQPQT[Pho]QEK.+2b4.heavy	42.1
**836.887099**	599.303518	13.6	sp|Q12888|TP53B_HUMAN.LDQELQQPQT[Pho]QEK.+2b5.heavy	42.1
**836.887099**	855.420673	13.6	sp|Q12888|TP53B_HUMAN.LDQELQQPQT[Pho]QEK.+2b7.heavy	42.1
**583.576746**	731.833179	15.48	sp|Q12888|TP53B_HUMAN.TSS[Pho]GTSLSAMHSSGSSGK.+3y16 -98+2.light	30.3
**583.576746**	697.322447	15.48	sp|Q12888|TP53B_HUMAN.TSS[Pho]GTSLSAMHSSGSSGK.+3y15+2.light	30.3
**583.576746**	618.287876	15.48	sp|Q12888|TP53B_HUMAN.TSS[Pho]GTSLSAMHSSGSSGK.+3y13+2.light	30.3
**583.576746**	518.22983	15.48	sp|Q12888|TP53B_HUMAN.TSS[Pho]GTSLSAMHSSGSSGK.+3y11+2.light	30.3
**583.576746**	488.224545	15.48	sp|Q12888|TP53B_HUMAN.TSS[Pho]GTSLSAMHSSGSSGK.+3y16 -98+3.light	30.3
**586.248145**	735.840278	15.48	sp|Q12888|TP53B_HUMAN.TSS[Pho]GTSLSAMHSSGSSGK.+3y16 -98+2.heavy	30.3
**586.248145**	701.329547	15.48	sp|Q12888|TP53B_HUMAN.TSS[Pho]GTSLSAMHSSGSSGK.+3y15+2.heavy	30.3
**586.248145**	622.294976	15.48	sp|Q12888|TP53B_HUMAN.TSS[Pho]GTSLSAMHSSGSSGK.+3y13+2.heavy	30.3
**586.248145**	522.236929	15.48	sp|Q12888|TP53B_HUMAN.TSS[Pho]GTSLSAMHSSGSSGK.+3y11+2.heavy	30.3
**586.248145**	490.895944	15.48	sp|Q12888|TP53B_HUMAN.TSS[Pho]GTSLSAMHSSGSSGK.+3y16 -98+3.heavy	30.3
**731.850513**	963.465923	26.7	sp|Q96T58|MINT_HUMAN.ELQEAAAVPTT[Pho]PR.+2y9.light	37.7
**731.850513**	892.428809	26.7	sp|Q96T58|MINT_HUMAN.ELQEAAAVPTT[Pho]PR.+2y8.light	37.7
**731.850513**	821.391695	26.7	sp|Q96T58|MINT_HUMAN.ELQEAAAVPTT[Pho]PR.+2y7.light	37.7
**731.850513**	750.354581	26.7	sp|Q96T58|MINT_HUMAN.ELQEAAAVPTT[Pho]PR.+2y6.light	37.7
**731.850513**	651.286168	26.7	sp|Q96T58|MINT_HUMAN.ELQEAAAVPTT[Pho]PR.+2y5.light	37.7
**736.854648**	973.474192	26.7	sp|Q96T58|MINT_HUMAN.ELQEAAAVPTT[Pho]PR.+2y9.heavy	37.7
**736.854648**	902.437078	26.7	sp|Q96T58|MINT_HUMAN.ELQEAAAVPTT[Pho]PR.+2y8.heavy	37.7
**736.854648**	831.399964	26.7	sp|Q96T58|MINT_HUMAN.ELQEAAAVPTT[Pho]PR.+2y7.heavy	37.7
**736.854648**	760.36285	26.7	sp|Q96T58|MINT_HUMAN.ELQEAAAVPTT[Pho]PR.+2y6.heavy	37.7
**736.854648**	661.294437	26.7	sp|Q96T58|MINT_HUMAN.ELQEAAAVPTT[Pho]PR.+2y5.heavy	37.7
**900.335818**	1193.435805	25.07	sp|Q9Y6X4|F169A_HUMAN.FQDSEFSSS[Pho]QGEDEK.+2y10.light	45.1
**900.335818**	1046.367391	25.07	sp|Q9Y6X4|F169A_HUMAN.FQDSEFSSS[Pho]QGEDEK.+2y9.light	45.1
**900.335818**	959.335362	25.07	sp|Q9Y6X4|F169A_HUMAN.FQDSEFSSS[Pho]QGEDEK.+2y8.light	45.1
**900.335818**	928.368303	25.07	sp|Q9Y6X4|F169A_HUMAN.FQDSEFSSS[Pho]QGEDEK.+2b8.light	45.1
**900.335818**	1223.42524	25.07	sp|Q9Y6X4|F169A_HUMAN.FQDSEFSSS[Pho]QGEDEK.+2b10.light	45.1
**904.342918**	1201.450004	25.07	sp|Q9Y6X4|F169A_HUMAN.FQDSEFSSS[Pho]QGEDEK.+2y10.heavy	45.1
**904.342918**	1054.38159	25.07	sp|Q9Y6X4|F169A_HUMAN.FQDSEFSSS[Pho]QGEDEK.+2y9.heavy	45.1
**904.342918**	967.349561	25.07	sp|Q9Y6X4|F169A_HUMAN.FQDSEFSSS[Pho]QGEDEK.+2y8.heavy	45.1
**904.342918**	928.368303	25.07	sp|Q9Y6X4|F169A_HUMAN.FQDSEFSSS[Pho]QGEDEK.+2b8.heavy	45.1
**904.342918**	1223.42524	25.07	sp|Q9Y6X4|F169A_HUMAN.FQDSEFSSS[Pho]QGEDEK.+2b10.heavy	45.1
**857.462971**	876.459047	47.92	sp|Q15554|TERF2_HUMAN.DLVLPTQALPAS[Pho]PALK.+2y8.light	43.2
**857.462971**	763.374983	47.92	sp|Q15554|TERF2_HUMAN.DLVLPTQALPAS[Pho]PALK.+2y7.light	43.2
**857.462971**	428.286745	47.92	sp|Q15554|TERF2_HUMAN.DLVLPTQALPAS[Pho]PALK.+2y4.light	43.2
**857.462971**	637.331228	47.92	sp|Q15554|TERF2_HUMAN.DLVLPTQALPAS[Pho]PALK.+2y12+2.light	43.2
**857.462971**	441.270761	47.92	sp|Q15554|TERF2_HUMAN.DLVLPTQALPAS[Pho]PALK.+2b4.light	43.2
**861.47007**	884.473246	47.92	sp|Q15554|TERF2_HUMAN.DLVLPTQALPAS[Pho]PALK.+2y8.heavy	43.2
**861.47007**	771.389182	47.92	sp|Q15554|TERF2_HUMAN.DLVLPTQALPAS[Pho]PALK.+2y7.heavy	43.2
**861.47007**	436.300944	47.92	sp|Q15554|TERF2_HUMAN.DLVLPTQALPAS[Pho]PALK.+2y4.heavy	43.2
**861.47007**	641.338328	47.92	sp|Q15554|TERF2_HUMAN.DLVLPTQALPAS[Pho]PALK.+2y12+2.heavy	43.2
**861.47007**	441.270761	47.92	sp|Q15554|TERF2_HUMAN.DLVLPTQALPAS[Pho]PALK.+2b4.heavy	43.2
**712.008315**	916.498589	48.27	sp|O15530|PDPK1_HUMAN.ANS[Pho]FVGTAQYVSPELLTEK.+3y8.light	36.8
**712.008315**	829.46656	48.27	sp|O15530|PDPK1_HUMAN.ANS[Pho]FVGTAQYVSPELLTEK.+3y7.light	36.8
**712.008315**	603.371203	48.27	sp|O15530|PDPK1_HUMAN.ANS[Pho]FVGTAQYVSPELLTEK.+3y5.light	36.8
**712.008315**	377.203075	48.27	sp|O15530|PDPK1_HUMAN.ANS[Pho]FVGTAQYVSPELLTEK.+3y3.light	36.8
**712.008315**	599.222505	48.27	sp|O15530|PDPK1_HUMAN.ANS[Pho]FVGTAQYVSPELLTEK.+3b5.light	36.8
**714.679715**	924.512788	48.27	sp|O15530|PDPK1_HUMAN.ANS[Pho]FVGTAQYVSPELLTEK.+3y8.heavy	36.8
**714.679715**	837.480759	48.27	sp|O15530|PDPK1_HUMAN.ANS[Pho]FVGTAQYVSPELLTEK.+3y7.heavy	36.8
**714.679715**	611.385402	48.27	sp|O15530|PDPK1_HUMAN.ANS[Pho]FVGTAQYVSPELLTEK.+3y5.heavy	36.8
**714.679715**	385.217274	48.27	sp|O15530|PDPK1_HUMAN.ANS[Pho]FVGTAQYVSPELLTEK.+3y3.heavy	36.8
**714.679715**	599.222505	48.27	sp|O15530|PDPK1_HUMAN.ANS[Pho]FVGTAQYVSPELLTEK.+3b5.heavy	36.8
**680.269219**	1102.429991	13.94	sp|Q14790|CASP8_HUMAN.EQDSES[Pho]QTLDK.+2y9.light	35.4
**680.269219**	987.403048	13.94	sp|Q14790|CASP8_HUMAN.EQDSES[Pho]QTLDK.+2y8.light	35.4
**680.269219**	771.328426	13.94	sp|Q14790|CASP8_HUMAN.EQDSES[Pho]QTLDK.+2y6.light	35.4
**680.269219**	604.330067	13.94	sp|Q14790|CASP8_HUMAN.EQDSES[Pho]QTLDK.+2y5.light	35.4

**680.269219**	476.271489	13.94	sp|Q14790|CASP8_HUMAN.EQDSES[Pho]QTLDK.+2y4.light	35.4
**684.276318**	1110.44419	13.94	sp|Q14790|CASP8_HUMAN.EQDSES[Pho]QTLDK.+2y9.heavy	35.4
**684.276318**	995.417247	13.94	sp|Q14790|CASP8_HUMAN.EQDSES[Pho]QTLDK.+2y8.heavy	35.4
**684.276318**	779.342625	13.94	sp|Q14790|CASP8_HUMAN.EQDSES[Pho]QTLDK.+2y6.heavy	35.4
**684.276318**	612.344266	13.94	sp|Q14790|CASP8_HUMAN.EQDSES[Pho]QTLDK.+2y5.heavy	35.4
**684.276318**	484.285688	13.94	sp|Q14790|CASP8_HUMAN.EQDSES[Pho]QTLDK.+2y4.heavy	35.4
**775.871111**	1080.497283	35.2	sp|P23396|RS3_HUMAN.DEILPTT[Pho]PISEQK.+2y9.light	39.6
**775.871111**	701.382831	35.2	sp|P23396|RS3_HUMAN.DEILPTT[Pho]PISEQK.+2y6.light	39.6
**775.871111**	597.294311	35.2	sp|P23396|RS3_HUMAN.DEILPTT[Pho]PISEQK.+2y10+2.light	39.6
**775.871111**	540.752279	35.2	sp|P23396|RS3_HUMAN.DEILPTT[Pho]PISEQK.+2y9+2.light	39.6
**775.871111**	358.160876	35.2	sp|P23396|RS3_HUMAN.DEILPTT[Pho]PISEQK.+2b3.light	39.6
**779.878211**	1088.511482	35.2	sp|P23396|RS3_HUMAN.DEILPTT[Pho]PISEQK.+2y9.heavy	39.6
**779.878211**	709.39703	35.2	sp|P23396|RS3_HUMAN.DEILPTT[Pho]PISEQK.+2y6.heavy	39.6
**779.878211**	601.301411	35.2	sp|P23396|RS3_HUMAN.DEILPTT[Pho]PISEQK.+2y10+2.heavy	39.6
**779.878211**	544.759379	35.2	sp|P23396|RS3_HUMAN.DEILPTT[Pho]PISEQK.+2y9+2.heavy	39.6
**779.878211**	358.160876	35.2	sp|P23396|RS3_HUMAN.DEILPTT[Pho]PISEQK.+2b3.heavy	39.6
**482.692222**	792.292375	15	sp|P53396|ACLY_HUMAN.TAS[Pho]FSESR.+2y6.light	26.7
**482.692222**	625.294016	15	sp|P53396|ACLY_HUMAN.TAS[Pho]FSESR.+2y5.light	26.7
**482.692222**	391.193573	15	sp|P53396|ACLY_HUMAN.TAS[Pho]FSESR.+2y3.light	26.7
**482.692222**	340.090428	15	sp|P53396|ACLY_HUMAN.TAS[Pho]FSESR.+2b3.light	26.7
**482.692222**	352.12037	15	sp|P53396|ACLY_HUMAN.TAS[Pho]FSESR.+2b6+2.light	26.7
**487.696356**	802.300644	15	sp|P53396|ACLY_HUMAN.TAS[Pho]FSESR.+2y6.heavy	26.7
**487.696356**	635.302285	15	sp|P53396|ACLY_HUMAN.TAS[Pho]FSESR.+2y5.heavy	26.7
**487.696356**	401.201842	15	sp|P53396|ACLY_HUMAN.TAS[Pho]FSESR.+2y3.heavy	26.7
**487.696356**	340.090428	15	sp|P53396|ACLY_HUMAN.TAS[Pho]FSESR.+2b3.heavy	26.7
**487.696356**	352.12037	15	sp|P53396|ACLY_HUMAN.TAS[Pho]FSESR.+2b6+2.heavy	26.7
**887.88664**	651.286168	28.71	sp|P17544|ATF7_HUMAN.TDSVIIADQT[Pho]PT[Pho]PTR.+2y5.light	44.6
**887.88664**	373.219394	28.71	sp|P17544|ATF7_HUMAN.TDSVIIADQT[Pho]PT[Pho]PTR.+2y3.light	44.6
**887.88664**	304.113926	28.71	sp|P17544|ATF7_HUMAN.TDSVIIADQT[Pho]PT[Pho]PTR.+2b3.light	44.6
**887.88664**	403.18234	28.71	sp|P17544|ATF7_HUMAN.TDSVIIADQT[Pho]PT[Pho]PTR.+2b4.light	44.6
**887.88664**	516.266404	28.71	sp|P17544|ATF7_HUMAN.TDSVIIADQT[Pho]PT[Pho]PTR.+2b5.light	44.6
**892.890774**	661.294437	28.71	sp|P17544|ATF7_HUMAN.TDSVIIADQT[Pho]PT[Pho]PTR.+2y5.heavy	44.6
**892.890774**	383.227663	28.71	sp|P17544|ATF7_HUMAN.TDSVIIADQT[Pho]PT[Pho]PTR.+2y3.heavy	44.6
**892.890774**	304.113926	28.71	sp|P17544|ATF7_HUMAN.TDSVIIADQT[Pho]PT[Pho]PTR.+2b3.heavy	44.6
**892.890774**	403.18234	28.71	sp|P17544|ATF7_HUMAN.TDSVIIADQT[Pho]PT[Pho]PTR.+2b4.heavy	44.6
**892.890774**	516.266404	28.71	sp|P17544|ATF7_HUMAN.TDSVIIADQT[Pho]PT[Pho]PTR.+2b5.heavy	44.6
**887.376439**	651.286168	25.28	sp|P15336|ATF2_HUMAN.NDSVIVADQT[Pho]PT[Pho]PTR.+2y5.light	44.5
**887.376439**	373.219394	25.28	sp|P15336|ATF2_HUMAN.NDSVIVADQT[Pho]PT[Pho]PTR.+2y3.light	44.5
**887.376439**	317.109175	25.28	sp|P15336|ATF2_HUMAN.NDSVIVADQT[Pho]PT[Pho]PTR.+2b3.light	44.5
**887.376439**	416.177589	25.28	sp|P15336|ATF2_HUMAN.NDSVIVADQT[Pho]PT[Pho]PTR.+2b4.light	44.5
**887.376439**	529.261653	25.28	sp|P15336|ATF2_HUMAN.NDSVIVADQT[Pho]PT[Pho]PTR.+2b5.light	44.5
**892.380574**	661.294437	25.28	sp|P15336|ATF2_HUMAN.NDSVIVADQT[Pho]PT[Pho]PTR.+2y5.heavy	44.5
**892.380574**	383.227663	25.28	sp|P15336|ATF2_HUMAN.NDSVIVADQT[Pho]PT[Pho]PTR.+2y3.heavy	44.5
**892.380574**	317.109175	25.28	sp|P15336|ATF2_HUMAN.NDSVIVADQT[Pho]PT[Pho]PTR.+2b3.heavy	44.5
**892.380574**	416.177589	25.28	sp|P15336|ATF2_HUMAN.NDSVIVADQT[Pho]PT[Pho]PTR.+2b4.heavy	44.5
**892.380574**	529.261653	25.28	sp|P15336|ATF2_HUMAN.NDSVIVADQT[Pho]PT[Pho]PTR.+2b5.heavy	44.5
**763.332409**	504.241252	26.82	sp|P46013|KI67_HUMAN.SGGSGHAVAEPAS[Pho]PEQELDQNK.+3y4.light	39.4
**763.332409**	718.308678	26.82	sp|P46013|KI67_HUMAN.SGGSGHAVAEPAS[Pho]PEQELDQNK.+3y12+2.light	39.4
**763.332409**	550.76456	26.82	sp|P46013|KI67_HUMAN.SGGSGHAVAEPAS[Pho]PEQELDQNK.+3y9+2.light	39.4
**763.332409**	653.300164	26.82	sp|P46013|KI67_HUMAN.SGGSGHAVAEPAS[Pho]PEQELDQNK.+3b8.light	39.4
**763.332409**	853.379871	26.82	sp|P46013|KI67_HUMAN.SGGSGHAVAEPAS[Pho]PEQELDQNK.+3b10.light	39.4
**766.003809**	512.255451	26.82	sp|P46013|KI67_HUMAN.SGGSGHAVAEPAS[Pho]PEQELDQNK.+3y4.heavy	39.4
**766.003809**	722.315778	26.82	sp|P46013|KI67_HUMAN.SGGSGHAVAEPAS[Pho]PEQELDQNK.+3y12+2.heavy	39.4
**766.003809**	554.771659	26.82	sp|P46013|KI67_HUMAN.SGGSGHAVAEPAS[Pho]PEQELDQNK.+3y9+2.heavy	39.4
**766.003809**	653.300164	26.82	sp|P46013|KI67_HUMAN.SGGSGHAVAEPAS[Pho]PEQELDQNK.+3b8.heavy	39.4
**766.003809**	853.379871	26.82	sp|P46013|KI67_HUMAN.SGGSGHAVAEPAS[Pho]PEQELDQNK.+3b10.heavy	39.4
**723.983841**	749.386202	39.89	sp|Q09161|NCBP1_HUMAN.KTS[Pho]DANETEDHLESLIC[CAM]K.+3y6.light	37.4

**723.983841**	307.143452	39.89	sp|Q09161|NCBP1_HUMAN.KTS[Pho]DANETEDHLESLIC[CAM]K.+3y2.light	37.4
**723.983841**	672.826834	39.89	sp|Q09161|NCBP1_HUMAN.KTS[Pho]DANETEDHLESLIC[CAM]K.+3y11+2.light	37.4
**723.983841**	310.675442	39.89	sp|Q09161|NCBP1_HUMAN.KTS[Pho]DANETEDHLESLIC[CAM]K.+3y5+2.light	37.4
**723.983841**	654.740629	39.89	sp|Q09161|NCBP1_HUMAN.KTS[Pho]DANETEDHLESLIC[CAM]K.+3b11+2.light	37.4
**726.655241**	757.400401	39.89	sp|Q09161|NCBP1_HUMAN.KTS[Pho]DANETEDHLESLIC[CAM]K.+3y6.heavy	37.4
**726.655241**	315.157651	39.89	sp|Q09161|NCBP1_HUMAN.KTS[Pho]DANETEDHLESLIC[CAM]K.+3y2.heavy	37.4
**726.655241**	676.833934	39.89	sp|Q09161|NCBP1_HUMAN.KTS[Pho]DANETEDHLESLIC[CAM]K.+3y11+2.heavy	37.4
**726.655241**	314.682542	39.89	sp|Q09161|NCBP1_HUMAN.KTS[Pho]DANETEDHLESLIC[CAM]K.+3y5+2.heavy	37.4
**726.655241**	654.740629	39.89	sp|Q09161|NCBP1_HUMAN.KTS[Pho]DANETEDHLESLIC[CAM]K.+3b11+2.heavy	37.4
**502.908207**	690.297067	29.15	sp|P29590|PML_HUMAN.AVSPPHLDGPPS[Pho]PR.+3y6.light	26.1
**502.908207**	668.805909	29.15	sp|P29590|PML_HUMAN.AVSPPHLDGPPS[Pho]PR.+3y12+2.light	26.1
**502.908207**	625.289895	29.15	sp|P29590|PML_HUMAN.AVSPPHLDGPPS[Pho]PR.+3y11+2.light	26.1
**502.908207**	576.763513	29.15	sp|P29590|PML_HUMAN.AVSPPHLDGPPS[Pho]PR.+3y10+2.light	26.1
**502.908207**	528.237131	29.15	sp|P29590|PML_HUMAN.AVSPPHLDGPPS[Pho]PR.+3y9+2.light	26.1
**506.244297**	700.305336	29.15	sp|P29590|PML_HUMAN.AVSPPHLDGPPS[Pho]PR.+3y6.heavy	26.1
**506.244297**	673.810043	29.15	sp|P29590|PML_HUMAN.AVSPPHLDGPPS[Pho]PR.+3y12+2.heavy	26.1
**506.244297**	630.294029	29.15	sp|P29590|PML_HUMAN.AVSPPHLDGPPS[Pho]PR.+3y11+2.heavy	26.1
**506.244297**	581.767647	29.15	sp|P29590|PML_HUMAN.AVSPPHLDGPPS[Pho]PR.+3y10+2.heavy	26.1
**506.244297**	533.241265	29.15	sp|P29590|PML_HUMAN.AVSPPHLDGPPS[Pho]PR.+3y9+2.heavy	26.1
**539.680997**	800.264446	19.58	sp|P61978|HNRPK_HUMAN.DYDDMS[Pho]PR.+2y6.light	29.2
**539.680997**	685.237503	19.58	sp|P61978|HNRPK_HUMAN.DYDDMS[Pho]PR.+2y5.light	29.2
**539.680997**	570.21056	19.58	sp|P61978|HNRPK_HUMAN.DYDDMS[Pho]PR.+2y4.light	29.2
**539.680997**	439.170075	19.58	sp|P61978|HNRPK_HUMAN.DYDDMS[Pho]PR.+2y3.light	29.2
**539.680997**	509.151434	19.58	sp|P61978|HNRPK_HUMAN.DYDDMS[Pho]PR.+2b4.light	29.2
**544.685131**	810.272715	19.58	sp|P61978|HNRPK_HUMAN.DYDDMS[Pho]PR.+2y6.heavy	29.2
**544.685131**	695.245772	19.58	sp|P61978|HNRPK_HUMAN.DYDDMS[Pho]PR.+2y5.heavy	29.2
**544.685131**	580.218829	19.58	sp|P61978|HNRPK_HUMAN.DYDDMS[Pho]PR.+2y4.heavy	29.2
**544.685131**	449.178344	19.58	sp|P61978|HNRPK_HUMAN.DYDDMS[Pho]PR.+2y3.heavy	29.2
**544.685131**	509.151434	19.58	sp|P61978|HNRPK_HUMAN.DYDDMS[Pho]PR.+2b4.heavy	29.2
**734.863424**	907.46486	48.64	sp|P16949|STMN1_HUMAN.ASGQAFELILS[Pho]PR.+2y7.light	37.8
**734.863424**	665.338203	48.64	sp|P16949|STMN1_HUMAN.ASGQAFELILS[Pho]PR.+2y5.light	37.8
**734.863424**	552.254139	48.64	sp|P16949|STMN1_HUMAN.ASGQAFELILS[Pho]PR.+2y4.light	37.8
**734.863424**	439.170075	48.64	sp|P16949|STMN1_HUMAN.ASGQAFELILS[Pho]PR.+2y3.light	37.8
**734.863424**	562.261987	48.64	sp|P16949|STMN1_HUMAN.ASGQAFELILS[Pho]PR.+2b6.light	37.8
**739.867558**	917.473129	48.64	sp|P16949|STMN1_HUMAN.ASGQAFELILS[Pho]PR.+2y7.heavy	37.8
**739.867558**	675.346472	48.64	sp|P16949|STMN1_HUMAN.ASGQAFELILS[Pho]PR.+2y5.heavy	37.8
**739.867558**	562.262408	48.64	sp|P16949|STMN1_HUMAN.ASGQAFELILS[Pho]PR.+2y4.heavy	37.8
**739.867558**	449.178344	48.64	sp|P16949|STMN1_HUMAN.ASGQAFELILS[Pho]PR.+2y3.heavy	37.8
**739.867558**	562.261987	48.64	sp|P16949|STMN1_HUMAN.ASGQAFELILS[Pho]PR.+2b6.heavy	37.8
**703.833801**	865.421933	44.19	sp|P16949|STMN1_HUMAN.ESVPEFPLS[Pho]PPK.+2y7.light	36.5
**703.833801**	718.353519	44.19	sp|P16949|STMN1_HUMAN.ESVPEFPLS[Pho]PPK.+2y6.light	36.5
**703.833801**	341.218332	44.19	sp|P16949|STMN1_HUMAN.ESVPEFPLS[Pho]PPK.+2y3.light	36.5
**703.833801**	546.262283	44.19	sp|P16949|STMN1_HUMAN.ESVPEFPLS[Pho]PPK.+2y9+2.light	36.5
**703.833801**	497.735901	44.19	sp|P16949|STMN1_HUMAN.ESVPEFPLS[Pho]PPK.+2y8+2.light	36.5
**707.8409**	873.436132	44.19	sp|P16949|STMN1_HUMAN.ESVPEFPLS[Pho]PPK.+2y7.heavy	36.5
**707.8409**	726.367718	44.19	sp|P16949|STMN1_HUMAN.ESVPEFPLS[Pho]PPK.+2y6.heavy	36.5
**707.8409**	349.23253	44.19	sp|P16949|STMN1_HUMAN.ESVPEFPLS[Pho]PPK.+2y3.heavy	36.5
**707.8409**	550.269382	44.19	sp|P16949|STMN1_HUMAN.ESVPEFPLS[Pho]PPK.+2y9+2.heavy	36.5
**707.8409**	501.743	44.19	sp|P16949|STMN1_HUMAN.ESVPEFPLS[Pho]PPK.+2y8+2.heavy	36.5
**767.389274**	1031.492138	50	sp|P04198|MYCN_HUMAN.FELLPTPPLS[Pho]PSR.+2y9.light	39.3
**767.389274**	833.391695	50	sp|P04198|MYCN_HUMAN.FELLPTPPLS[Pho]PSR.+2y7.light	39.3
**767.389274**	359.203744	50	sp|P04198|MYCN_HUMAN.FELLPTPPLS[Pho]PSR.+2y3.light	39.3
**767.389274**	417.199486	50	sp|P04198|MYCN_HUMAN.FELLPTPPLS[Pho]PSR.+2y7+2.light	39.3
**767.389274**	390.202347	50	sp|P04198|MYCN_HUMAN.FELLPTPPLS[Pho]PSR.+2b3.light	39.3
**772.393409**	1041.500407	50	sp|P04198|MYCN_HUMAN.FELLPTPPLS[Pho]PSR.+2y9.heavy	39.3
**772.393409**	843.399964	50	sp|P04198|MYCN_HUMAN.FELLPTPPLS[Pho]PSR.+2y7.heavy	39.3
**772.393409**	369.212013	50	sp|P04198|MYCN_HUMAN.FELLPTPPLS[Pho]PSR.+2y3.heavy	39.3

**772.393409**	422.20362	50	sp|P04198|MYCN_HUMAN.FELLPTPPLS[Pho]PSR.+2y7+2.heavy	39.3
**772.393409**	390.202347	50	sp|P04198|MYCN_HUMAN.FELLPTPPLS[Pho]PSR.+2b3.heavy	39.3
**797.357856**	895.374331	47.41	sp|P40763|STAT3_HUMAN.FIC[CAM]VTPTTC[CAM]SNTIDLPMS[Pho]PR.+3y7.light	41.2
**797.357856**	667.263324	47.41	sp|P40763|STAT3_HUMAN.FIC[CAM]VTPTTC[CAM]SNTIDLPMS[Pho]PR.+3y5.light	41.2
**797.357856**	439.170075	47.41	sp|P40763|STAT3_HUMAN.FIC[CAM]VTPTTC[CAM]SNTIDLPMS[Pho]PR.+3y3.light	41.2
**797.357856**	334.1353	47.41	sp|P40763|STAT3_HUMAN.FIC[CAM]VTPTTC[CAM]SNTIDLPMS[Pho]PR.+3y5+2.ligh	41.2
**797.357856**	421.190402	47.41	sp|P40763|STAT3_HUMAN.FIC[CAM]VTPTTC[CAM]SNTIDLPMS[Pho]PR.+3b3.light	41.2
**800.693946**	905.3826	47.41	sp|P40763|STAT3_HUMAN.FIC[CAM]VTPTTC[CAM]SNTIDLPMS[Pho]PR.+3y7.heavy	41.2
**800.693946**	677.271593	47.41	sp|P40763|STAT3_HUMAN.FIC[CAM]VTPTTC[CAM]SNTIDLPMS[Pho]PR.+3y5.heavy	41.2
**800.693946**	449.178344	47.41	sp|P40763|STAT3_HUMAN.FIC[CAM]VTPTTC[CAM]SNTIDLPMS[Pho]PR.+3y3.heavy	41.2
**800.693946**	339.139434	47.41	sp|P40763|STAT3_HUMAN.FIC[CAM]VTPTTC[CAM]SNTIDLPMS[Pho]PR.+3y5+2.hea	41.2
**800.693946**	421.190402	47.41	sp|P40763|STAT3_HUMAN.FIC[CAM]VTPTTC[CAM]SNTIDLPMS[Pho]PR.+3b3.heavy	41.2
**502.197671**	757.251239	15.51	sp|Q53EL6|PDCD4_HUMAN.FVS[Pho]EGDGGR.+2y7.light	27.6
**502.197671**	590.252879	15.51	sp|Q53EL6|PDCD4_HUMAN.FVS[Pho]EGDGGR.+2y6.light	27.6
**502.197671**	461.210286	15.51	sp|Q53EL6|PDCD4_HUMAN.FVS[Pho]EGDGGR.+2y5.light	27.6
**502.197671**	404.188822	15.51	sp|Q53EL6|PDCD4_HUMAN.FVS[Pho]EGDGGR.+2y4.light	27.6
**502.197671**	600.20652	15.51	sp|Q53EL6|PDCD4_HUMAN.FVS[Pho]EGDGGR.+2b5.light	27.6
**507.201806**	767.259508	15.51	sp|Q53EL6|PDCD4_HUMAN.FVS[Pho]EGDGGR.+2y7.heavy	27.6
**507.201806**	600.261148	15.51	sp|Q53EL6|PDCD4_HUMAN.FVS[Pho]EGDGGR.+2y6.heavy	27.6
**507.201806**	471.218555	15.51	sp|Q53EL6|PDCD4_HUMAN.FVS[Pho]EGDGGR.+2y5.heavy	27.6
**507.201806**	414.197091	15.51	sp|Q53EL6|PDCD4_HUMAN.FVS[Pho]EGDGGR.+2y4.heavy	27.6
**507.201806**	600.20652	15.51	sp|Q53EL6|PDCD4_HUMAN.FVS[Pho]EGDGGR.+2b5.heavy	27.6
**681.281409**	964.395795	27.6	sp|P49840|GSK3A_HUMAN.GEPNVSY[Pho]IC[CAM]SR.+2y7.light	35.5
**681.281409**	865.327381	27.6	sp|P49840|GSK3A_HUMAN.GEPNVSY[Pho]IC[CAM]SR.+2y6.light	35.5
**681.281409**	778.295352	27.6	sp|P49840|GSK3A_HUMAN.GEPNVSY[Pho]IC[CAM]SR.+2y5.light	35.5
**681.281409**	535.265693	27.6	sp|P49840|GSK3A_HUMAN.GEPNVSY[Pho]IC[CAM]SR.+2y4.light	35.5
**681.281409**	588.249381	27.6	sp|P49840|GSK3A_HUMAN.GEPNVSY[Pho]IC[CAM]SR.+2y9+2.light	35.5
**686.285544**	974.404064	27.6	sp|P49840|GSK3A_HUMAN.GEPNVSY[Pho]IC[CAM]SR.+2y7.heavy	35.5
**686.285544**	875.33565	27.6	sp|P49840|GSK3A_HUMAN.GEPNVSY[Pho]IC[CAM]SR.+2y6.heavy	35.5
**686.285544**	788.303621	27.6	sp|P49840|GSK3A_HUMAN.GEPNVSY[Pho]IC[CAM]SR.+2y5.heavy	35.5
**686.285544**	545.273962	27.6	sp|P49840|GSK3A_HUMAN.GEPNVSY[Pho]IC[CAM]SR.+2y4.heavy	35.5
**686.285544**	593.253516	27.6	sp|P49840|GSK3A_HUMAN.GEPNVSY[Pho]IC[CAM]SR.+2y9+2.heavy	35.5
**682.316031**	749.43045	44.15	sp|Q9UQE7|SMC3_HUMAN.GSGS[Pho]QSSVPSVDQFTGVGIR.+3y7.light	35.3
**682.316031**	602.362036	44.15	sp|Q9UQE7|SMC3_HUMAN.GSGS[Pho]QSSVPSVDQFTGVGIR.+3y6.light	35.3
**682.316031**	501.314357	44.15	sp|Q9UQE7|SMC3_HUMAN.GSGS[Pho]QSSVPSVDQFTGVGIR.+3y5.light	35.3
**682.316031**	345.22448	44.15	sp|Q9UQE7|SMC3_HUMAN.GSGS[Pho]QSSVPSVDQFTGVGIR.+3y3.light	35.3
**682.316031**	638.338226	44.15	sp|Q9UQE7|SMC3_HUMAN.GSGS[Pho]QSSVPSVDQFTGVGIR.+3y12+2.light	35.3
**685.65212**	759.438719	44.15	sp|Q9UQE7|SMC3_HUMAN.GSGS[Pho]QSSVPSVDQFTGVGIR.+3y7.heavy	35.3
**685.65212**	612.370305	44.15	sp|Q9UQE7|SMC3_HUMAN.GSGS[Pho]QSSVPSVDQFTGVGIR.+3y6.heavy	35.3
**685.65212**	511.322626	44.15	sp|Q9UQE7|SMC3_HUMAN.GSGS[Pho]QSSVPSVDQFTGVGIR.+3y5.heavy	35.3
**685.65212**	355.232748	44.15	sp|Q9UQE7|SMC3_HUMAN.GSGS[Pho]QSSVPSVDQFTGVGIR.+3y3.heavy	35.3
**685.65212**	643.342361	44.15	sp|Q9UQE7|SMC3_HUMAN.GSGS[Pho]QSSVPSVDQFTGVGIR.+3y12+2.heavy	35.3
**705.355826**	882.515576	44.3	sp|P00533|EGFR_HUMAN.ELVEPLT[Pho]PSGEAPNQALLR.+3y8.light	36.5
**705.355826**	811.478462	44.3	sp|P00533|EGFR_HUMAN.ELVEPLT[Pho]PSGEAPNQALLR.+3y7.light	36.5
**705.355826**	714.425699	44.3	sp|P00533|EGFR_HUMAN.ELVEPLT[Pho]PSGEAPNQALLR.+3y6.light	36.5
**705.355826**	401.28708	44.3	sp|P00533|EGFR_HUMAN.ELVEPLT[Pho]PSGEAPNQALLR.+3y3.light	36.5
**705.355826**	406.242869	44.3	sp|P00533|EGFR_HUMAN.ELVEPLT[Pho]PSGEAPNQALLR.+3y7+2.light	36.5
**708.691916**	892.523845	44.3	sp|P00533|EGFR_HUMAN.ELVEPLT[Pho]PSGEAPNQALLR.+3y8.heavy	36.5
**708.691916**	821.486731	44.3	sp|P00533|EGFR_HUMAN.ELVEPLT[Pho]PSGEAPNQALLR.+3y7.heavy	36.5
**708.691916**	724.433968	44.3	sp|P00533|EGFR_HUMAN.ELVEPLT[Pho]PSGEAPNQALLR.+3y6.heavy	36.5
**708.691916**	411.295349	44.3	sp|P00533|EGFR_HUMAN.ELVEPLT[Pho]PSGEAPNQALLR.+3y3.heavy	36.5
**708.691916**	411.247004	44.3	sp|P00533|EGFR_HUMAN.ELVEPLT[Pho]PSGEAPNQALLR.+3y7+2.heavy	36.5
**645.771732**	974.397903	20.37	sp|P00533|EGFR_HUMAN.GSTAENAEY[Pho]LR.+2y7.light	33.9
**645.771732**	845.35531	20.37	sp|P00533|EGFR_HUMAN.GSTAENAEY[Pho]LR.+2y6.light	33.9
**645.771732**	731.312382	20.37	sp|P00533|EGFR_HUMAN.GSTAENAEY[Pho]LR.+2y5.light	33.9
**645.771732**	660.275268	20.37	sp|P00533|EGFR_HUMAN.GSTAENAEY[Pho]LR.+2y4.light	33.9
**645.771732**	531.232675	20.37	sp|P00533|EGFR_HUMAN.GSTAENAEY[Pho]LR.+2y3.light	33.9

**650.775866**	984.406172	20.37	sp|P00533|EGFR_HUMAN.GSTAENAEY[Pho]LR.+2y7.heavy	33.9
**650.775866**	855.363579	20.37	sp|P00533|EGFR_HUMAN.GSTAENAEY[Pho]LR.+2y6.heavy	33.9
**650.775866**	741.320651	20.37	sp|P00533|EGFR_HUMAN.GSTAENAEY[Pho]LR.+2y5.heavy	33.9
**650.775866**	670.283537	20.37	sp|P00533|EGFR_HUMAN.GSTAENAEY[Pho]LR.+2y4.heavy	33.9
**650.775866**	541.240944	20.37	sp|P00533|EGFR_HUMAN.GSTAENAEY[Pho]LR.+2y3.heavy	33.9
**941.900074**	1177.477275	40.37	sp|Q13535|ATR_HUMAN.GVELC[CAM]FPENET[Pho]PPEGK.+2y10.light	46.9
**941.900074**	527.282388	40.37	sp|Q13535|ATR_HUMAN.GVELC[CAM]FPENET[Pho]PPEGK.+2y5.light	46.9
**941.900074**	589.242276	40.37	sp|Q13535|ATR_HUMAN.GVELC[CAM]FPENET[Pho]PPEGK.+2y10+2.light	46.9
**941.900074**	399.223811	40.37	sp|Q13535|ATR_HUMAN.GVELC[CAM]FPENET[Pho]PPEGK.+2b4.light	46.9
**941.900074**	706.322873	40.37	sp|Q13535|ATR_HUMAN.GVELC[CAM]FPENET[Pho]PPEGK.+2b6.light	46.9
**945.907174**	1185.491474	40.37	sp|Q13535|ATR_HUMAN.GVELC[CAM]FPENET[Pho]PPEGK.+2y10.heavy	46.9
**945.907174**	535.296587	40.37	sp|Q13535|ATR_HUMAN.GVELC[CAM]FPENET[Pho]PPEGK.+2y5.heavy	46.9
**945.907174**	593.249375	40.37	sp|Q13535|ATR_HUMAN.GVELC[CAM]FPENET[Pho]PPEGK.+2y10+2.heavy	46.9
**945.907174**	399.223811	40.37	sp|Q13535|ATR_HUMAN.GVELC[CAM]FPENET[Pho]PPEGK.+2b4.heavy	46.9
**945.907174**	706.322873	40.37	sp|Q13535|ATR_HUMAN.GVELC[CAM]FPENET[Pho]PPEGK.+2b6.heavy	46.9
**464.730619**	628.402838	22.89	sp|P42226|STAT6_HUMAN.GY[Pho]VPATIK.+2y6.light	25.9
**464.730619**	529.334424	22.89	sp|P42226|STAT6_HUMAN.GY[Pho]VPATIK.+2y5.light	25.9
**464.730619**	361.244546	22.89	sp|P42226|STAT6_HUMAN.GY[Pho]VPATIK.+2y3.light	25.9
**464.730619**	301.058399	22.89	sp|P42226|STAT6_HUMAN.GY[Pho]VPATIK.+2b2.light	25.9
**464.730619**	400.126813	22.89	sp|P42226|STAT6_HUMAN.GY[Pho]VPATIK.+2b3.light	25.9
**468.737718**	636.417037	22.89	sp|P42226|STAT6_HUMAN.GY[Pho]VPATIK.+2y6.heavy	25.9
**468.737718**	537.348623	22.89	sp|P42226|STAT6_HUMAN.GY[Pho]VPATIK.+2y5.heavy	25.9
**468.737718**	369.258745	22.89	sp|P42226|STAT6_HUMAN.GY[Pho]VPATIK.+2y3.heavy	25.9
**468.737718**	301.058399	22.89	sp|P42226|STAT6_HUMAN.GY[Pho]VPATIK.+2b2.heavy	25.9
**468.737718**	400.126813	22.89	sp|P42226|STAT6_HUMAN.GY[Pho]VPATIK.+2b3.heavy	25.9
**575.768264**	1037.445187	22.68	sp|Q13627|DYR1A_HUMAN.IYQY[Pho]IQSR.+2y7.light	30.8
**575.768264**	874.381859	22.68	sp|Q13627|DYR1A_HUMAN.IYQY[Pho]IQSR.+2y6.light	30.8
**575.768264**	746.323281	22.68	sp|Q13627|DYR1A_HUMAN.IYQY[Pho]IQSR.+2y5.light	30.8
**575.768264**	519.226232	22.68	sp|Q13627|DYR1A_HUMAN.IYQY[Pho]IQSR.+2y7+2.light	30.8
**575.768264**	437.694567	22.68	sp|Q13627|DYR1A_HUMAN.IYQY[Pho]IQSR.+2y6+2.light	30.8
**580.772398**	1047.453456	22.68	sp|Q13627|DYR1A_HUMAN.IYQY[Pho]IQSR.+2y7.heavy	30.8
**580.772398**	884.390128	22.68	sp|Q13627|DYR1A_HUMAN.IYQY[Pho]IQSR.+2y6.heavy	30.8
**580.772398**	756.33155	22.68	sp|Q13627|DYR1A_HUMAN.IYQY[Pho]IQSR.+2y5.heavy	30.8
**580.772398**	524.230366	22.68	sp|Q13627|DYR1A_HUMAN.IYQY[Pho]IQSR.+2y7+2.heavy	30.8
**580.772398**	442.698702	22.68	sp|Q13627|DYR1A_HUMAN.IYQY[Pho]IQSR.+2y6+2.heavy	30.8
**553.231711**	311.123762	12.12	sp|P16104|H2AX_HUMAN.KATQAS[Pho]QEY.+2y2.light	29.8
**553.231711**	500.282723	12.12	sp|P16104|H2AX_HUMAN.KATQAS[Pho]QEY.+2b5.light	29.8
**553.231711**	667.281082	12.12	sp|P16104|H2AX_HUMAN.KATQAS[Pho]QEY.+2b6.light	29.8
**553.231711**	795.33966	12.12	sp|P16104|H2AX_HUMAN.KATQAS[Pho]QEY.+2b7.light	29.8
**553.231711**	924.382253	12.12	sp|P16104|H2AX_HUMAN.KATQAS[Pho]QEY.+2b8.light	29.8
**557.238811**	311.123762	12.12	sp|P16104|H2AX_HUMAN.KATQAS[Pho]QEY.+2y2.heavy	29.8
**557.238811**	508.296922	12.12	sp|P16104|H2AX_HUMAN.KATQAS[Pho]QEY.+2b5.heavy	29.8
**557.238811**	675.295281	12.12	sp|P16104|H2AX_HUMAN.KATQAS[Pho]QEY.+2b6.heavy	29.8
**557.238811**	803.353859	12.12	sp|P16104|H2AX_HUMAN.KATQAS[Pho]QEY.+2b7.heavy	29.8
**557.238811**	932.396452	12.12	sp|P16104|H2AX_HUMAN.KATQAS[Pho]QEY.+2b8.heavy	29.8
**593.214877**	509.21194	11.77	sp|P16104|H2AX_HUMAN.KAT[Pho]QAS[Pho]QEY.+2b4.light	31.6
**593.214877**	580.249054	11.77	sp|P16104|H2AX_HUMAN.KAT[Pho]QAS[Pho]QEY.+2b5.light	31.6
**593.214877**	747.247413	11.77	sp|P16104|H2AX_HUMAN.KAT[Pho]QAS[Pho]QEY.+2b6.light	31.6
**593.214877**	777.329095	11.77	sp|P16104|H2AX_HUMAN.KAT[Pho]QAS[Pho]QEY.+2b7 -98.light	31.6
**593.214877**	1004.348584	11.77	sp|P16104|H2AX_HUMAN.KAT[Pho]QAS[Pho]QEY.+2b8.light	31.6
**593.214877**	906.371688	11.77	sp|P16104|H2AX_HUMAN.KAT[Pho]QAS[Pho]QEY.+2b8 -98.light	31.6
**597.221976**	517.226139	11.77	sp|P16104|H2AX_HUMAN.KAT[Pho]QAS[Pho]QEY.+2b4.heavy	31.6
**597.221976**	588.263253	11.77	sp|P16104|H2AX_HUMAN.KAT[Pho]QAS[Pho]QEY.+2b5.heavy	31.6
**597.221976**	755.261612	11.77	sp|P16104|H2AX_HUMAN.KAT[Pho]QAS[Pho]QEY.+2b6.heavy	31.6
**597.221976**	785.343294	11.77	sp|P16104|H2AX_HUMAN.KAT[Pho]QAS[Pho]QEY.+2b7 -98.heavy	31.6
**597.221976**	1012.362783	11.77	sp|P16104|H2AX_HUMAN.KAT[Pho]QAS[Pho]QEY.+2b8.heavy	31.6
**597.221976**	914.385887	11.77	sp|P16104|H2AX_HUMAN.KAT[Pho]QAS[Pho]QEY.+2b8 -98.heavy	31.6

**790.37181**	1026.557835	37.56	sp|Q8IVT2|MISP_HUMAN.ALS[Pho]SDSILSPAPDAR.+2y10.light	40.3
**790.37181**	826.441743	37.56	sp|Q8IVT2|MISP_HUMAN.ALS[Pho]SDSILSPAPDAR.+2y8.light	40.3
**790.37181**	713.357679	37.56	sp|Q8IVT2|MISP_HUMAN.ALS[Pho]SDSILSPAPDAR.+2y7.light	40.3
**790.37181**	626.32565	37.56	sp|Q8IVT2|MISP_HUMAN.ALS[Pho]SDSILSPAPDAR.+2y6.light	40.3
**790.37181**	458.235772	37.56	sp|Q8IVT2|MISP_HUMAN.ALS[Pho]SDSILSPAPDAR.+2y4.light	40.3
**795.375944**	1036.566104	37.56	sp|Q8IVT2|MISP_HUMAN.ALS[Pho]SDSILSPAPDAR.+2y10.heavy	40.3
**795.375944**	836.450012	37.56	sp|Q8IVT2|MISP_HUMAN.ALS[Pho]SDSILSPAPDAR.+2y8.heavy	40.3
**795.375944**	723.365948	37.56	sp|Q8IVT2|MISP_HUMAN.ALS[Pho]SDSILSPAPDAR.+2y7.heavy	40.3
**795.375944**	636.333919	37.56	sp|Q8IVT2|MISP_HUMAN.ALS[Pho]SDSILSPAPDAR.+2y6.heavy	40.3
**795.375944**	468.244041	37.56	sp|Q8IVT2|MISP_HUMAN.ALS[Pho]SDSILSPAPDAR.+2y4.heavy	40.3
**553.933192**	764.389707	32.99	sp|O60825|F262_HUMAN.RNS[Pho]FTPLSSSNTIR.+3y7.light	28.8
**553.933192**	677.357679	32.99	sp|O60825|F262_HUMAN.RNS[Pho]FTPLSSSNTIR.+3y6.light	28.8
**553.933192**	590.32565	32.99	sp|O60825|F262_HUMAN.RNS[Pho]FTPLSSSNTIR.+3y5.light	28.8
**553.933192**	487.766905	32.99	sp|O60825|F262_HUMAN.RNS[Pho]FTPLSSSNTIR.+3y9+2.light	28.8
**553.933192**	686.265766	32.99	sp|O60825|F262_HUMAN.RNS[Pho]FTPLSSSNTIR.+3b5.light	28.8
**557.269282**	774.397976	32.99	sp|O60825|F262_HUMAN.RNS[Pho]FTPLSSSNTIR.+3y7.heavy	28.8
**557.269282**	687.365948	32.99	sp|O60825|F262_HUMAN.RNS[Pho]FTPLSSSNTIR.+3y6.heavy	28.8
**557.269282**	600.333919	32.99	sp|O60825|F262_HUMAN.RNS[Pho]FTPLSSSNTIR.+3y5.heavy	28.8
**557.269282**	492.77104	32.99	sp|O60825|F262_HUMAN.RNS[Pho]FTPLSSSNTIR.+3y9+2.heavy	28.8
**557.269282**	686.265766	32.99	sp|O60825|F262_HUMAN.RNS[Pho]FTPLSSSNTIR.+3b5.heavy	28.8
**566.273326**	670.340632	22.46	sp|P27708|PYR1_HUMAN.IHRAS[Pho]DPGLPAEEPK.+3y6.light	29.4
**566.273326**	335.673954	22.46	sp|P27708|PYR1_HUMAN.IHRAS[Pho]DPGLPAEEPK.+3y6+2.light	29.4
**566.273326**	380.660528	22.46	sp|P27708|PYR1_HUMAN.IHRAS[Pho]DPGLPAEEPK.+3b6+2.light	29.4
**566.273326**	457.697641	22.46	sp|P27708|PYR1_HUMAN.IHRAS[Pho]DPGLPAEEPK.+3b8+2.light	29.4
**566.273326**	514.239673	22.46	sp|P27708|PYR1_HUMAN.IHRAS[Pho]DPGLPAEEPK.+3b9+2.light	29.4
**568.944726**	678.354831	22.46	sp|P27708|PYR1_HUMAN.IHRAS[Pho]DPGLPAEEPK.+3y6.heavy	29.4
**568.944726**	339.681053	22.46	sp|P27708|PYR1_HUMAN.IHRAS[Pho]DPGLPAEEPK.+3y6+2.heavy	29.4
**568.944726**	380.660528	22.46	sp|P27708|PYR1_HUMAN.IHRAS[Pho]DPGLPAEEPK.+3b6+2.heavy	29.4
**568.944726**	457.697641	22.46	sp|P27708|PYR1_HUMAN.IHRAS[Pho]DPGLPAEEPK.+3b8+2.heavy	29.4
**568.944726**	514.239673	22.46	sp|P27708|PYR1_HUMAN.IHRAS[Pho]DPGLPAEEPK.+3b9+2.heavy	29.4
**724.975852**	784.431178	49.5	sp|Q15418|KS6A1_HUMAN.AYS[Pho]FC[CAM]GTVEYMAPEVVNR.+3y7.light	37.5
**724.975852**	713.394064	49.5	sp|Q15418|KS6A1_HUMAN.AYS[Pho]FC[CAM]GTVEYMAPEVVNR.+3y6.light	37.5
**724.975852**	616.3413	49.5	sp|Q15418|KS6A1_HUMAN.AYS[Pho]FC[CAM]GTVEYMAPEVVNR.+3y5.light	37.5
**724.975852**	487.298707	49.5	sp|Q15418|KS6A1_HUMAN.AYS[Pho]FC[CAM]GTVEYMAPEVVNR.+3y4.light	37.5
**724.975852**	388.230293	49.5	sp|Q15418|KS6A1_HUMAN.AYS[Pho]FC[CAM]GTVEYMAPEVVNR.+3y3.light	37.5
**728.311942**	794.439447	49.5	sp|Q15418|KS6A1_HUMAN.AYS[Pho]FC[CAM]GTVEYMAPEVVNR.+3y7.heavy	37.5
**728.311942**	723.402333	49.5	sp|Q15418|KS6A1_HUMAN.AYS[Pho]FC[CAM]GTVEYMAPEVVNR.+3y6.heavy	37.5
**728.311942**	626.349569	49.5	sp|Q15418|KS6A1_HUMAN.AYS[Pho]FC[CAM]GTVEYMAPEVVNR.+3y5.heavy	37.5
**728.311942**	497.306976	49.5	sp|Q15418|KS6A1_HUMAN.AYS[Pho]FC[CAM]GTVEYMAPEVVNR.+3y4.heavy	37.5
**728.311942**	398.238562	49.5	sp|Q15418|KS6A1_HUMAN.AYS[Pho]FC[CAM]GTVEYMAPEVVNR.+3y3.heavy	37.5
**700.308678**	845.472708	37.72	sp|Q9BVJ6|UT14A_HUMAN.DSGS[Pho]QEVLSELR.+2y7.light	36.3
**700.308678**	716.430115	37.72	sp|Q9BVJ6|UT14A_HUMAN.DSGS[Pho]QEVLSELR.+2y6.light	36.3
**700.308678**	617.361701	37.72	sp|Q9BVJ6|UT14A_HUMAN.DSGS[Pho]QEVLSELR.+2y5.light	36.3
**700.308678**	504.277637	37.72	sp|Q9BVJ6|UT14A_HUMAN.DSGS[Pho]QEVLSELR.+2y4.light	36.3
**700.308678**	642.795207	37.72	sp|Q9BVJ6|UT14A_HUMAN.DSGS[Pho]QEVLSELR.+2y11+2.light	36.3
**705.312813**	855.480977	37.72	sp|Q9BVJ6|UT14A_HUMAN.DSGS[Pho]QEVLSELR.+2y7.heavy	36.3
**705.312813**	726.438384	37.72	sp|Q9BVJ6|UT14A_HUMAN.DSGS[Pho]QEVLSELR.+2y6.heavy	36.3
**705.312813**	627.36997	37.72	sp|Q9BVJ6|UT14A_HUMAN.DSGS[Pho]QEVLSELR.+2y5.heavy	36.3
**705.312813**	514.285906	37.72	sp|Q9BVJ6|UT14A_HUMAN.DSGS[Pho]QEVLSELR.+2y4.heavy	36.3
**705.312813**	647.799341	37.72	sp|Q9BVJ6|UT14A_HUMAN.DSGS[Pho]QEVLSELR.+2y11+2.heavy	36.3
**564.225571**	846.40258	21.9	sp|Q13144|EI2BE_HUMAN.GGS[Pho]PQMDDIK.+2y7.light	30.3
**564.225571**	749.349816	21.9	sp|Q13144|EI2BE_HUMAN.GGS[Pho]PQMDDIK.+2y6.light	30.3
**564.225571**	621.291239	21.9	sp|Q13144|EI2BE_HUMAN.GGS[Pho]PQMDDIK.+2y5.light	30.3
**564.225571**	490.250754	21.9	sp|Q13144|EI2BE_HUMAN.GGS[Pho]PQMDDIK.+2y4.light	30.3
**564.225571**	423.704928	21.9	sp|Q13144|EI2BE_HUMAN.GGS[Pho]PQMDDIK.+2y7+2.light	30.3
**568.232671**	854.416779	21.9	sp|Q13144|EI2BE_HUMAN.GGS[Pho]PQMDDIK.+2y7.heavy	30.3
**568.232671**	757.364015	21.9	sp|Q13144|EI2BE_HUMAN.GGS[Pho]PQMDDIK.+2y6.heavy	30.3

**568.232671**	629.305438	21.9	sp|Q13144|EI2BE_HUMAN.GGS[Pho]PQMDDIK.+2y5.heavy	30.3
**568.232671**	498.264953	21.9	sp|Q13144|EI2BE_HUMAN.GGS[Pho]PQMDDIK.+2y4.heavy	30.3
**568.232671**	427.712027	21.9	sp|Q13144|EI2BE_HUMAN.GGS[Pho]PQMDDIK.+2y7+2.heavy	30.3
**434.218493**	594.28207	25.53	sp|Q8WXE1|ATRIP_HUMAN.LAAPSVSHVS[Pho]PR.+3y11+2.light	22.6
**434.218493**	558.763513	25.53	sp|Q8WXE1|ATRIP_HUMAN.LAAPSVSHVS[Pho]PR.+3y10+2.light	22.6
**434.218493**	523.244956	25.53	sp|Q8WXE1|ATRIP_HUMAN.LAAPSVSHVS[Pho]PR.+3y9+2.light	22.6
**434.218493**	474.718574	25.53	sp|Q8WXE1|ATRIP_HUMAN.LAAPSVSHVS[Pho]PR.+3y8+2.light	22.6
**434.218493**	563.768264	25.53	sp|Q8WXE1|ATRIP_HUMAN.LAAPSVSHVS[Pho]PR.+3b11+2.light	22.6
**437.554583**	599.286204	25.53	sp|Q8WXE1|ATRIP_HUMAN.LAAPSVSHVS[Pho]PR.+3y11+2.heavy	22.6
**437.554583**	563.767647	25.53	sp|Q8WXE1|ATRIP_HUMAN.LAAPSVSHVS[Pho]PR.+3y10+2.heavy	22.6
**437.554583**	528.24909	25.53	sp|Q8WXE1|ATRIP_HUMAN.LAAPSVSHVS[Pho]PR.+3y9+2.heavy	22.6
**437.554583**	479.722708	25.53	sp|Q8WXE1|ATRIP_HUMAN.LAAPSVSHVS[Pho]PR.+3y8+2.heavy	22.6
**437.554583**	563.768264	25.53	sp|Q8WXE1|ATRIP_HUMAN.LAAPSVSHVS[Pho]PR.+3b11+2.heavy	22.6
**661.599439**	881.399937	47.74	sp|Q92878|RAD50_HUMAN.LFDVC[CAM]GS[Pho]QDFESDLDR.+3y7.light	34.2
**661.599439**	734.331523	47.74	sp|Q92878|RAD50_HUMAN.LFDVC[CAM]GS[Pho]QDFESDLDR.+3y6.light	34.2
**661.599439**	605.28893	47.74	sp|Q92878|RAD50_HUMAN.LFDVC[CAM]GS[Pho]QDFESDLDR.+3y5.light	34.2
**661.599439**	518.256902	47.74	sp|Q92878|RAD50_HUMAN.LFDVC[CAM]GS[Pho]QDFESDLDR.+3y4.light	34.2
**661.599439**	403.229959	47.74	sp|Q92878|RAD50_HUMAN.LFDVC[CAM]GS[Pho]QDFESDLDR.+3y3.light	34.2
**664.935529**	891.408206	47.74	sp|Q92878|RAD50_HUMAN.LFDVC[CAM]GS[Pho]QDFESDLDR.+3y7.heavy	34.2
**664.935529**	744.339792	47.74	sp|Q92878|RAD50_HUMAN.LFDVC[CAM]GS[Pho]QDFESDLDR.+3y6.heavy	34.2
**664.935529**	615.297199	47.74	sp|Q92878|RAD50_HUMAN.LFDVC[CAM]GS[Pho]QDFESDLDR.+3y5.heavy	34.2
**664.935529**	528.265171	47.74	sp|Q92878|RAD50_HUMAN.LFDVC[CAM]GS[Pho]QDFESDLDR.+3y4.heavy	34.2
**664.935529**	413.238228	47.74	sp|Q92878|RAD50_HUMAN.LFDVC[CAM]GS[Pho]QDFESDLDR.+3y3.heavy	34.2
**516.723522**	919.355704	19.6	sp|Q96B36|AKTS1_HUMAN.LNTS[Pho]DFQK.+2y7.light	28.2
**516.723522**	704.265098	19.6	sp|Q96B36|AKTS1_HUMAN.LNTS[Pho]DFQK.+2y5.light	28.2
**516.723522**	537.266738	19.6	sp|Q96B36|AKTS1_HUMAN.LNTS[Pho]DFQK.+2y4.light	28.2
**516.723522**	422.239795	19.6	sp|Q96B36|AKTS1_HUMAN.LNTS[Pho]DFQK.+2y3.light	28.2
**516.723522**	329.181946	19.6	sp|Q96B36|AKTS1_HUMAN.LNTS[Pho]DFQK.+2b3.light	28.2
**520.730621**	927.369903	19.6	sp|Q96B36|AKTS1_HUMAN.LNTS[Pho]DFQK.+2y7.heavy	28.2
**520.730621**	712.279297	19.6	sp|Q96B36|AKTS1_HUMAN.LNTS[Pho]DFQK.+2y5.heavy	28.2
**520.730621**	545.280937	19.6	sp|Q96B36|AKTS1_HUMAN.LNTS[Pho]DFQK.+2y4.heavy	28.2
**520.730621**	430.253994	19.6	sp|Q96B36|AKTS1_HUMAN.LNTS[Pho]DFQK.+2y3.heavy	28.2
**520.730621**	329.181946	19.6	sp|Q96B36|AKTS1_HUMAN.LNTS[Pho]DFQK.+2b3.heavy	28.2
**733.297142**	832.379536	21.43	sp|Q9UKV3|ACINU_HUMAN.LSEGS[Pho]QPAEEEDQETPSR.+3y7.light	37.9
**733.297142**	589.294016	21.43	sp|Q9UKV3|ACINU_HUMAN.LSEGS[Pho]QPAEEEDQETPSR.+3y5.light	37.9
**733.297142**	460.251423	21.43	sp|Q9UKV3|ACINU_HUMAN.LSEGS[Pho]QPAEEEDQETPSR.+3y4.light	37.9
**733.297142**	359.203744	21.43	sp|Q9UKV3|ACINU_HUMAN.LSEGS[Pho]QPAEEEDQETPSR.+3y3.light	37.9
**733.297142**	359.179935	21.43	sp|Q9UKV3|ACINU_HUMAN.LSEGS[Pho]QPAEEEDQETPSR.+3y6+2.light	37.9
**736.633231**	842.387805	21.43	sp|Q9UKV3|ACINU_HUMAN.LSEGS[Pho]QPAEEEDQETPSR.+3y7.heavy	37.9
**736.633231**	599.302285	21.43	sp|Q9UKV3|ACINU_HUMAN.LSEGS[Pho]QPAEEEDQETPSR.+3y5.heavy	37.9
**736.633231**	470.259692	21.43	sp|Q9UKV3|ACINU_HUMAN.LSEGS[Pho]QPAEEEDQETPSR.+3y4.heavy	37.9
**736.633231**	369.212013	21.43	sp|Q9UKV3|ACINU_HUMAN.LSEGS[Pho]QPAEEEDQETPSR.+3y3.heavy	37.9
**736.633231**	364.184069	21.43	sp|Q9UKV3|ACINU_HUMAN.LSEGS[Pho]QPAEEEDQETPSR.+3y6+2.heavy	37.9
**771.6968**	955.44795	36.57	sp|Q14980|NUMA1_HUMAN.LSQLEEHLS[Pho]QLQDNPPQEK.+3y8.light	39.9
**771.6968**	827.389373	36.57	sp|Q14980|NUMA1_HUMAN.LSQLEEHLS[Pho]QLQDNPPQEK.+3y7.light	39.9
**771.6968**	712.36243	36.57	sp|Q14980|NUMA1_HUMAN.LSQLEEHLS[Pho]QLQDNPPQEK.+3y6.light	39.9
**771.6968**	598.319502	36.57	sp|Q14980|NUMA1_HUMAN.LSQLEEHLS[Pho]QLQDNPPQEK.+3y5.light	39.9
**771.6968**	501.266738	36.57	sp|Q14980|NUMA1_HUMAN.LSQLEEHLS[Pho]QLQDNPPQEK.+3y4.light	39.9
**774.368199**	963.462149	36.57	sp|Q14980|NUMA1_HUMAN.LSQLEEHLS[Pho]QLQDNPPQEK.+3y8.heavy	39.9
**774.368199**	835.403572	36.57	sp|Q14980|NUMA1_HUMAN.LSQLEEHLS[Pho]QLQDNPPQEK.+3y7.heavy	39.9
**774.368199**	720.376629	36.57	sp|Q14980|NUMA1_HUMAN.LSQLEEHLS[Pho]QLQDNPPQEK.+3y6.heavy	39.9
**774.368199**	606.333701	36.57	sp|Q14980|NUMA1_HUMAN.LSQLEEHLS[Pho]QLQDNPPQEK.+3y5.heavy	39.9
**774.368199**	509.280937	36.57	sp|Q14980|NUMA1_HUMAN.LSQLEEHLS[Pho]QLQDNPPQEK.+3y4.heavy	39.9
**601.249578**	848.429463	31.32	sp|Q13263|TIF1B_HUMAN.S[Pho]GEGEVSGLMR.+2y8.light	32
**601.249578**	791.408	31.32	sp|Q13263|TIF1B_HUMAN.S[Pho]GEGEVSGLMR.+2y7.light	32
**601.249578**	662.365407	31.32	sp|Q13263|TIF1B_HUMAN.S[Pho]GEGEVSGLMR.+2y6.light	32
**601.249578**	563.296993	31.32	sp|Q13263|TIF1B_HUMAN.S[Pho]GEGEVSGLMR.+2y5.light	32

**601.249578**	354.069692	31.32	sp|Q13263|TIF1B_HUMAN.S[Pho]GEGEVSGLMR.+2b3.light	32
**606.253712**	858.437732	31.32	sp|Q13263|TIF1B_HUMAN.S[Pho]GEGEVSGLMR.+2y8.heavy	32
**606.253712**	801.416269	31.32	sp|Q13263|TIF1B_HUMAN.S[Pho]GEGEVSGLMR.+2y7.heavy	32
**606.253712**	672.373676	31.32	sp|Q13263|TIF1B_HUMAN.S[Pho]GEGEVSGLMR.+2y6.heavy	32
**606.253712**	573.305262	31.32	sp|Q13263|TIF1B_HUMAN.S[Pho]GEGEVSGLMR.+2y5.heavy	32
**606.253712**	354.069692	31.32	sp|Q13263|TIF1B_HUMAN.S[Pho]GEGEVSGLMR.+2b3.heavy	32
**1010.810383**	643.268195	52.7	sp|Q13263|TIF1B_HUMAN.FSAVLVEPPPMSLPGAGLSS[Pho]QELSGGPGDGP.+3y8.li	52.1
**1010.810383**	499.214703	52.7	sp|Q13263|TIF1B_HUMAN.FSAVLVEPPPMSLPGAGLSS[Pho]QELSGGPGDGP.+3y6.li	52.1
**1010.810383**	442.193239	52.7	sp|Q13263|TIF1B_HUMAN.FSAVLVEPPPMSLPGAGLSS[Pho]QELSGGPGDGP.+3y5.li	52.1
**1010.810383**	518.29731	52.7	sp|Q13263|TIF1B_HUMAN.FSAVLVEPPPMSLPGAGLSS[Pho]QELSGGPGDGP.+3b5.l	52.1
**1010.810383**	746.408317	52.7	sp|Q13263|TIF1B_HUMAN.FSAVLVEPPPMSLPGAGLSS[Pho]QELSGGPGDGP.+3b7.l	52.1
**1014.152792**	643.268195	52.7	sp|Q13263|TIF1B_HUMAN.FSAVLVEPPPMSLPGAGLSS[Pho]QELSGGPGDGP.+3y8.h	52.1
**1014.152792**	499.214703	52.7	sp|Q13263|TIF1B_HUMAN.FSAVLVEPPPMSLPGAGLSS[Pho]QELSGGPGDGP.+3y6.h	52.1
**1014.152792**	442.193239	52.7	sp|Q13263|TIF1B_HUMAN.FSAVLVEPPPMSLPGAGLSS[Pho]QELSGGPGDGP.+3y5.h	52.1
**1014.152792**	528.324538	52.7	sp|Q13263|TIF1B_HUMAN.FSAVLVEPPPMSLPGAGLSS[Pho]QELSGGPGDGP.+3b5.h	52.1
**1014.152792**	756.435545	52.7	sp|Q13263|TIF1B_HUMAN.FSAVLVEPPPMSLPGAGLSS[Pho]QELSGGPGDGP.+3b7.h	52.1
**856.000948**	835.390435	25.8	e.GTMDDISQEEGSS[Pho]QGEDSVSGSQR.+3y8.light	44.2
**856.000948**	720.363492	25.8	e.GTMDDISQEEGSS[Pho]QGEDSVSGSQR.+3y7.light	44.2
**856.000948**	534.26305	25.8	e.GTMDDISQEEGSS[Pho]QGEDSVSGSQR.+3y5.light	44.2
**856.000948**	447.231021	25.8	e.GTMDDISQEEGSS[Pho]QGEDSVSGSQR.+3y4.light	44.2
**856.000948**	720.286881	25.8	e.GTMDDISQEEGSS[Pho]QGEDSVSGSQR.+3b7.light	44.2
**859.337037**	845.398704	25.8	e.GTMDDISQEEGSS[Pho]QGEDSVSGSQR.+3y8.heavy	44.2
**859.337037**	730.371761	25.8	e.GTMDDISQEEGSS[Pho]QGEDSVSGSQR.+3y7.heavy	44.2
**859.337037**	544.271319	25.8	e.GTMDDISQEEGSS[Pho]QGEDSVSGSQR.+3y5.heavy	44.2
**859.337037**	457.23929	25.8	e.GTMDDISQEEGSS[Pho]QGEDSVSGSQR.+3y4.heavy	44.2
**859.337037**	720.286881	25.8	e.GTMDDISQEEGSS[Pho]QGEDSVSGSQR.+3b7.heavy	44.2
**651.789924**	1074.486718	24.91	sp|O95453|PARN_HUMAN.NNS[Pho]FTAPSTVGK.+2y10.light	34.2
**651.789924**	907.488358	24.91	sp|O95453|PARN_HUMAN.NNS[Pho]FTAPSTVGK.+2y9.light	34.2
**651.789924**	760.419945	24.91	sp|O95453|PARN_HUMAN.NNS[Pho]FTAPSTVGK.+2y8.light	34.2
**651.789924**	659.372266	24.91	sp|O95453|PARN_HUMAN.NNS[Pho]FTAPSTVGK.+2y7.light	34.2
**651.789924**	588.335152	24.91	sp|O95453|PARN_HUMAN.NNS[Pho]FTAPSTVGK.+2y6.light	34.2
**655.797024**	1082.500917	24.91	sp|O95453|PARN_HUMAN.NNS[Pho]FTAPSTVGK.+2y10.heavy	34.2
**655.797024**	915.502558	24.91	sp|O95453|PARN_HUMAN.NNS[Pho]FTAPSTVGK.+2y9.heavy	34.2
**655.797024**	768.434144	24.91	sp|O95453|PARN_HUMAN.NNS[Pho]FTAPSTVGK.+2y8.heavy	34.2
**655.797024**	667.386465	24.91	sp|O95453|PARN_HUMAN.NNS[Pho]FTAPSTVGK.+2y7.heavy	34.2
**655.797024**	596.349351	24.91	sp|O95453|PARN_HUMAN.NNS[Pho]FTAPSTVGK.+2y6.heavy	34.2
**497.73389**	643.340966	23.71	sp|P17535|JUND_HUMAN.LAS[Pho]PELER.+2y5.light	27.4
**497.73389**	417.245609	23.71	sp|P17535|JUND_HUMAN.LAS[Pho]PELER.+2y3.light	27.4
**497.73389**	322.174121	23.71	sp|P17535|JUND_HUMAN.LAS[Pho]PELER.+2y5+2.light	27.4
**497.73389**	352.126813	23.71	sp|P17535|JUND_HUMAN.LAS[Pho]PELER.+2b3.light	27.4
**502.738024**	653.349235	23.71	sp|P17535|JUND_HUMAN.LAS[Pho]PELER.+2y5.heavy	27.4
**502.738024**	427.253878	23.71	sp|P17535|JUND_HUMAN.LAS[Pho]PELER.+2y3.heavy	27.4
**502.738024**	327.178255	23.71	sp|P17535|JUND_HUMAN.LAS[Pho]PELER.+2y5+2.heavy	27.4
**502.738024**	352.126813	23.71	sp|P17535|JUND_HUMAN.LAS[Pho]PELER.+2b3.heavy	27.4
**602.753342**	883.426821	13.61	sp|Q96EB6|SIR1_HUMAN.S[Pho]PGEPGGAAPER.+2y9.light	32
**602.753342**	754.384228	13.61	sp|Q96EB6|SIR1_HUMAN.S[Pho]PGEPGGAAPER.+2y8.light	32
**602.753342**	377.695752	13.61	sp|Q96EB6|SIR1_HUMAN.S[Pho]PGEPGGAAPER.+2y8+2.light	32
**602.753342**	451.122456	13.61	sp|Q96EB6|SIR1_HUMAN.S[Pho]PGEPGGAAPER.+2b4.light	32
**602.753342**	451.176208	13.61	sp|Q96EB6|SIR1_HUMAN.S[Pho]PGEPGGAAPER.+2b10+2.light	32
**607.757476**	893.43509	13.61	sp|Q96EB6|SIR1_HUMAN.S[Pho]PGEPGGAAPER.+2y9.heavy	32
**607.757476**	764.392497	13.61	sp|Q96EB6|SIR1_HUMAN.S[Pho]PGEPGGAAPER.+2y8.heavy	32
**607.757476**	382.699886	13.61	sp|Q96EB6|SIR1_HUMAN.S[Pho]PGEPGGAAPER.+2y8+2.heavy	32
**607.757476**	451.122456	13.61	sp|Q96EB6|SIR1_HUMAN.S[Pho]PGEPGGAAPER.+2b4.heavy	32
**607.757476**	451.176208	13.61	sp|Q96EB6|SIR1_HUMAN.S[Pho]PGEPGGAAPER.+2b10+2.heavy	32
**809.329504**	832.379536	23.17	sp|O15355|PPM1G_HUMAN.SGGGTGEEPGS[Pho]QGLNGEAGPEDSTR.+3y8.light	41.8
**809.329504**	761.342422	23.17	sp|O15355|PPM1G_HUMAN.SGGGTGEEPGS[Pho]QGLNGEAGPEDSTR.+3y7.light	41.8
**809.329504**	704.320959	23.17	sp|O15355|PPM1G_HUMAN.SGGGTGEEPGS[Pho]QGLNGEAGPEDSTR.+3y6.light	41.8

**809.329504**	876.365244	23.17	sp|O15355|PPM1G_HUMAN.SGGGTGEEPGS[Pho]QGLNGEAGPEDSTR.+3y17+2.ligl	41.8
**809.329504**	352.664117	23.17	sp|O15355|PPM1G_HUMAN.SGGGTGEEPGS[Pho]QGLNGEAGPEDSTR.+3y6+2.ligh	41.8
**812.665594**	842.387805	23.17	sp|O15355|PPM1G_HUMAN.SGGGTGEEPGS[Pho]QGLNGEAGPEDSTR.+3y8.heavy	41.8
**812.665594**	771.350692	23.17	sp|O15355|PPM1G_HUMAN.SGGGTGEEPGS[Pho]QGLNGEAGPEDSTR.+3y7.heavy	41.8
**812.665594**	714.329228	23.17	sp|O15355|PPM1G_HUMAN.SGGGTGEEPGS[Pho]QGLNGEAGPEDSTR.+3y6.heavy	41.8
**812.665594**	881.369379	23.17	sp|O15355|PPM1G_HUMAN.SGGGTGEEPGS[Pho]QGLNGEAGPEDSTR.+3y17+2.he	41.8
**812.665594**	357.668252	23.17	sp|O15355|PPM1G_HUMAN.SGGGTGEEPGS[Pho]QGLNGEAGPEDSTR.+3y6+2.hea	41.8
**694.315585**	864.467289	41.23	sp|Q13315|ATM_HUMAN.SLAFEEGS[Pho]QSTTISSLSEK.+3y8.light	35.9
**694.315585**	763.41961	41.23	sp|Q13315|ATM_HUMAN.SLAFEEGS[Pho]QSTTISSLSEK.+3y7.light	35.9
**694.315585**	650.335546	41.23	sp|Q13315|ATM_HUMAN.SLAFEEGS[Pho]QSTTISSLSEK.+3y6.light	35.9
**694.315585**	563.303518	41.23	sp|Q13315|ATM_HUMAN.SLAFEEGS[Pho]QSTTISSLSEK.+3y5.light	35.9
**694.315585**	363.187425	41.23	sp|Q13315|ATM_HUMAN.SLAFEEGS[Pho]QSTTISSLSEK.+3y3.light	35.9
**696.986985**	973.529166	41.23	sp|Q13315|ATM_HUMAN.SLAFEEGS[Pho]QSTTISSLSEK.+3y9.heavy	35.9
**696.986985**	771.433809	41.23	sp|Q13315|ATM_HUMAN.SLAFEEGS[Pho]QSTTISSLSEK.+3y7.heavy	35.9
**696.986985**	658.349745	41.23	sp|Q13315|ATM_HUMAN.SLAFEEGS[Pho]QSTTISSLSEK.+3y6.heavy	35.9
**696.986985**	571.317717	41.23	sp|Q13315|ATM_HUMAN.SLAFEEGS[Pho]QSTTISSLSEK.+3y5.heavy	35.9
**696.986985**	371.201624	41.23	sp|Q13315|ATM_HUMAN.SLAFEEGS[Pho]QSTTISSLSEK.+3y3.heavy	35.9
**680.792664**	851.425758	37.3	sp|Q13315|ATM_HUMAN.NLS[Pho]DIDQSFNK.+2y7.light	35.5
**680.792664**	738.341694	37.3	sp|Q13315|ATM_HUMAN.NLS[Pho]DIDQSFNK.+2y6.light	35.5
**680.792664**	623.314751	37.3	sp|Q13315|ATM_HUMAN.NLS[Pho]DIDQSFNK.+2y5.light	35.5
**680.792664**	495.256174	37.3	sp|Q13315|ATM_HUMAN.NLS[Pho]DIDQSFNK.+2y4.light	35.5
**680.792664**	408.224145	37.3	sp|Q13315|ATM_HUMAN.NLS[Pho]DIDQSFNK.+2y3.light	35.5
**684.799764**	859.439957	37.3	sp|Q13315|ATM_HUMAN.NLS[Pho]DIDQSFNK.+2y7.heavy	35.5
**684.799764**	746.355893	37.3	sp|Q13315|ATM_HUMAN.NLS[Pho]DIDQSFNK.+2y6.heavy	35.5
**684.799764**	631.32895	37.3	sp|Q13315|ATM_HUMAN.NLS[Pho]DIDQSFNK.+2y5.heavy	35.5
**684.799764**	503.270373	37.3	sp|Q13315|ATM_HUMAN.NLS[Pho]DIDQSFNK.+2y4.heavy	35.5
**684.799764**	416.238344	37.3	sp|Q13315|ATM_HUMAN.NLS[Pho]DIDQSFNK.+2y3.heavy	35.5
**770.855796**	942.371688	31.1	sp|Q6PKG0|LARP1_HUMAN.SLPTTVPES[Pho]PNYR.+2y7.light	39.4
**770.855796**	549.277972	31.1	sp|Q6PKG0|LARP1_HUMAN.SLPTTVPES[Pho]PNYR.+2y4.light	39.4
**770.855796**	670.797749	31.1	sp|Q6PKG0|LARP1_HUMAN.SLPTTVPES[Pho]PNYR.+2y11+2.light	39.4
**770.855796**	399.223811	31.1	sp|Q6PKG0|LARP1_HUMAN.SLPTTVPES[Pho]PNYR.+2b4.light	39.4
**770.855796**	599.339903	31.1	sp|Q6PKG0|LARP1_HUMAN.SLPTTVPES[Pho]PNYR.+2b6.light	39.4
**775.85993**	952.379957	31.1	sp|Q6PKG0|LARP1_HUMAN.SLPTTVPES[Pho]PNYR.+2y7.heavy	39.4
**775.85993**	559.286241	31.1	sp|Q6PKG0|LARP1_HUMAN.SLPTTVPES[Pho]PNYR.+2y4.heavy	39.4
**775.85993**	675.801884	31.1	sp|Q6PKG0|LARP1_HUMAN.SLPTTVPES[Pho]PNYR.+2y11+2.heavy	39.4
**775.85993**	399.223811	31.1	sp|Q6PKG0|LARP1_HUMAN.SLPTTVPES[Pho]PNYR.+2b4.heavy	39.4
**775.85993**	300.17359	31.1	sp|Q6PKG0|LARP1_HUMAN.SLPTTVPES[Pho]PNYR.+2b6+2.heavy	39.4
**828.742521**	1054.589135	51.52	sp|P15056|BRAF_HUMAN.SSS[Pho]APNVHINTIEPVNIDDLIR.+3y9.light	42.8
**828.742521**	858.467957	51.52	sp|P15056|BRAF_HUMAN.SSS[Pho]APNVHINTIEPVNIDDLIR.+3y7.light	42.8
**828.742521**	631.340966	51.52	sp|P15056|BRAF_HUMAN.SSS[Pho]APNVHINTIEPVNIDDLIR.+3y5.light	42.8
**828.742521**	516.314023	51.52	sp|P15056|BRAF_HUMAN.SSS[Pho]APNVHINTIEPVNIDDLIR.+3y4.light	42.8
**828.742521**	527.798206	51.52	sp|P15056|BRAF_HUMAN.SSS[Pho]APNVHINTIEPVNIDDLIR.+3y9+2.light	42.8
**832.07861**	1064.597404	51.52	sp|P15056|BRAF_HUMAN.SSS[Pho]APNVHINTIEPVNIDDLIR.+3y9.heavy	42.8
**832.07861**	868.476226	51.52	sp|P15056|BRAF_HUMAN.SSS[Pho]APNVHINTIEPVNIDDLIR.+3y7.heavy	42.8
**832.07861**	641.349235	51.52	sp|P15056|BRAF_HUMAN.SSS[Pho]APNVHINTIEPVNIDDLIR.+3y5.heavy	42.8
**832.07861**	526.322292	51.52	sp|P15056|BRAF_HUMAN.SSS[Pho]APNVHINTIEPVNIDDLIR.+3y4.heavy	42.8
**832.07861**	532.80234	51.52	sp|P15056|BRAF_HUMAN.SSS[Pho]APNVHINTIEPVNIDDLIR.+3y9+2.heavy	42.8
**556.260803**	923.434623	13.13	sp|P49815|TSC2_HUMAN.STS[Pho]LNERPK.+2y7.light	30
**556.260803**	756.436263	13.13	sp|P49815|TSC2_HUMAN.STS[Pho]LNERPK.+2y6.light	30
**556.260803**	643.352199	13.13	sp|P49815|TSC2_HUMAN.STS[Pho]LNERPK.+2y5.light	30
**556.260803**	529.309272	13.13	sp|P49815|TSC2_HUMAN.STS[Pho]LNERPK.+2y4.light	30
**556.260803**	400.266679	13.13	sp|P49815|TSC2_HUMAN.STS[Pho]LNERPK.+2y3.light	30
**560.267902**	931.448822	13.13	sp|P49815|TSC2_HUMAN.STS[Pho]LNERPK.+2y7.heavy	30
**560.267902**	764.450462	13.13	sp|P49815|TSC2_HUMAN.STS[Pho]LNERPK.+2y6.heavy	30
**560.267902**	651.366398	13.13	sp|P49815|TSC2_HUMAN.STS[Pho]LNERPK.+2y5.heavy	30
**560.267902**	537.323471	13.13	sp|P49815|TSC2_HUMAN.STS[Pho]LNERPK.+2y4.heavy	30
**560.267902**	408.280878	13.13	sp|P49815|TSC2_HUMAN.STS[Pho]LNERPK.+2y3.heavy	30

**798.996698**	1004.391558	34.31	sp|P78527|PRKDC_HUMAN.LTPLPEDNS[Pho]MNVDQDGDPSDR.+3y9.light	41.2
**798.996698**	761.306037	34.31	sp|P78527|PRKDC_HUMAN.LTPLPEDNS[Pho]MNVDQDGDPSDR.+3y7.light	41.2
**798.996698**	646.279094	34.31	sp|P78527|PRKDC_HUMAN.LTPLPEDNS[Pho]MNVDQDGDPSDR.+3y6.light	41.2
**798.996698**	474.230687	34.31	sp|P78527|PRKDC_HUMAN.LTPLPEDNS[Pho]MNVDQDGDPSDR.+3y4.light	41.2
**798.996698**	761.30201	34.31	sp|P78527|PRKDC_HUMAN.LTPLPEDNS[Pho]MNVDQDGDPSDR.+3y20+3.light	41.2
**802.332787**	1014.399827	34.31	sp|P78527|PRKDC_HUMAN.LTPLPEDNS[Pho]MNVDQDGDPSDR.+3y9.heavy	41.2
**802.332787**	771.314306	34.31	sp|P78527|PRKDC_HUMAN.LTPLPEDNS[Pho]MNVDQDGDPSDR.+3y7.heavy	41.2
**802.332787**	656.287363	34.31	sp|P78527|PRKDC_HUMAN.LTPLPEDNS[Pho]MNVDQDGDPSDR.+3y6.heavy	41.2
**802.332787**	484.238956	34.31	sp|P78527|PRKDC_HUMAN.LTPLPEDNS[Pho]MNVDQDGDPSDR.+3y4.heavy	41.2
**802.332787**	764.638099	34.31	sp|P78527|PRKDC_HUMAN.LTPLPEDNS[Pho]MNVDQDGDPSDR.+3y20+3.heavy	41.2
**751.338835**	446.272158	41.65	sp|P27361|MK03_HUMAN.IADPEHDHTGFLTEY[Pho]VATR.+3y4.light	38.8
**751.338835**	976.930555	41.65	sp|P27361|MK03_HUMAN.IADPEHDHTGFLTEY[Pho]VATR.+3y16+2.light	38.8
**751.338835**	300.155397	41.65	sp|P27361|MK03_HUMAN.IADPEHDHTGFLTEY[Pho]VATR.+3b3.light	38.8
**751.338835**	537.235969	41.65	sp|P27361|MK03_HUMAN.IADPEHDHTGFLTEY[Pho]VATR.+3b10+2.light	38.8
**751.338835**	610.770176	41.65	sp|P27361|MK03_HUMAN.IADPEHDHTGFLTEY[Pho]VATR.+3b11+2.light	38.8
**754.674925**	456.280427	41.65	sp|P27361|MK03_HUMAN.IADPEHDHTGFLTEY[Pho]VATR.+3y4.heavy	38.8
**754.674925**	981.934689	41.65	sp|P27361|MK03_HUMAN.IADPEHDHTGFLTEY[Pho]VATR.+3y16+2.heavy	38.8
**754.674925**	300.155397	41.65	sp|P27361|MK03_HUMAN.IADPEHDHTGFLTEY[Pho]VATR.+3b3.heavy	38.8
**754.674925**	537.235969	41.65	sp|P27361|MK03_HUMAN.IADPEHDHTGFLTEY[Pho]VATR.+3b10+2.heavy	38.8
**754.674925**	610.770176	41.65	sp|P27361|MK03_HUMAN.IADPEHDHTGFLTEY[Pho]VATR.+3b11+2.heavy	38.8
**563.577252**	863.408	26.42	sp|P52564|MP2K6_HUMAN.T[Pho]IDAGC[CAM]KPYMAPER.+3y7.light	29.2
**563.577252**	754.355236	26.42	sp|P52564|MP2K6_HUMAN.T[Pho]IDAGC[CAM]KPYMAPER.+3y13+2.light	29.2
**563.577252**	697.813204	26.42	sp|P52564|MP2K6_HUMAN.T[Pho]IDAGC[CAM]KPYMAPER.+3y12+2.light	29.2
**563.577252**	640.299732	26.42	sp|P52564|MP2K6_HUMAN.T[Pho]IDAGC[CAM]KPYMAPER.+3y11+2.light	29.2
**563.577252**	604.781175	26.42	sp|P52564|MP2K6_HUMAN.T[Pho]IDAGC[CAM]KPYMAPER.+3y10+2.light	29.2
**566.913342**	873.416269	26.42	sp|P52564|MP2K6_HUMAN.T[Pho]IDAGC[CAM]KPYMAPER.+3y7.heavy	29.2
**566.913342**	759.35937	26.42	sp|P52564|MP2K6_HUMAN.T[Pho]IDAGC[CAM]KPYMAPER.+3y13+2.heavy	29.2
**566.913342**	702.817338	26.42	sp|P52564|MP2K6_HUMAN.T[Pho]IDAGC[CAM]KPYMAPER.+3y12+2.heavy	29.2
**566.913342**	645.303867	26.42	sp|P52564|MP2K6_HUMAN.T[Pho]IDAGC[CAM]KPYMAPER.+3y11+2.heavy	29.2
**566.913342**	609.78531	26.42	sp|P52564|MP2K6_HUMAN.T[Pho]IDAGC[CAM]KPYMAPER.+3y10+2.heavy	29.2
**569.562726**	863.408	25.29	sp|P46734|MP2K3_HUMAN.T[Pho]MDAGC[CAM]KPYMAPER.+3y7.light	29.5
**569.562726**	763.333446	25.29	sp|P46734|MP2K3_HUMAN.T[Pho]MDAGC[CAM]KPYMAPER.+3y13+2.light	29.5
**569.562726**	697.813204	25.29	sp|P46734|MP2K3_HUMAN.T[Pho]MDAGC[CAM]KPYMAPER.+3y12+2.light	29.5
**569.562726**	640.299732	25.29	sp|P46734|MP2K3_HUMAN.T[Pho]MDAGC[CAM]KPYMAPER.+3y11+2.light	29.5
**569.562726**	432.207638	25.29	sp|P46734|MP2K3_HUMAN.T[Pho]MDAGC[CAM]KPYMAPER.+3y7+2.light	29.5
**572.898816**	873.416269	25.29	sp|P46734|MP2K3_HUMAN.T[Pho]MDAGC[CAM]KPYMAPER.+3y7.heavy	29.5
**572.898816**	768.337581	25.29	sp|P46734|MP2K3_HUMAN.T[Pho]MDAGC[CAM]KPYMAPER.+3y13+2.heavy	29.5
**572.898816**	702.817338	25.29	sp|P46734|MP2K3_HUMAN.T[Pho]MDAGC[CAM]KPYMAPER.+3y12+2.heavy	29.5
**572.898816**	645.303867	25.29	sp|P46734|MP2K3_HUMAN.T[Pho]MDAGC[CAM]KPYMAPER.+3y11+2.heavy	29.5
**572.898816**	437.211772	25.29	sp|P46734|MP2K3_HUMAN.T[Pho]MDAGC[CAM]KPYMAPER.+3y7+2.heavy	29.5
**525.55427**	719.368243	19.37	sp|P35568|IRS1_HUMAN.HSS[Pho]ETFSSTPSATR.+3y7.light	27.3
**525.55427**	531.288536	19.37	sp|P35568|IRS1_HUMAN.HSS[Pho]ETFSSTPSATR.+3y5.light	27.3
**525.55427**	434.235772	19.37	sp|P35568|IRS1_HUMAN.HSS[Pho]ETFSSTPSATR.+3y4.light	27.3
**525.55427**	622.186847	19.37	sp|P35568|IRS1_HUMAN.HSS[Pho]ETFSSTPSATR.+3b5.light	27.3
**525.55427**	769.255261	19.37	sp|P35568|IRS1_HUMAN.HSS[Pho]ETFSSTPSATR.+3b6.light	27.3
**528.890359**	729.376512	19.37	sp|P35568|IRS1_HUMAN.HSS[Pho]ETFSSTPSATR.+3y7.heavy	27.3
**528.890359**	541.296805	19.37	sp|P35568|IRS1_HUMAN.HSS[Pho]ETFSSTPSATR.+3y5.heavy	27.3
**528.890359**	444.244041	19.37	sp|P35568|IRS1_HUMAN.HSS[Pho]ETFSSTPSATR.+3y4.heavy	27.3
**528.890359**	622.186847	19.37	sp|P35568|IRS1_HUMAN.HSS[Pho]ETFSSTPSATR.+3b5.heavy	27.3
**528.890359**	769.255261	19.37	sp|P35568|IRS1_HUMAN.HSS[Pho]ETFSSTPSATR.+3b6.heavy	27.3
**827.33361**	1199.549849	8.67	sp|P23588|IF4B_HUMAN.TGS[Pho]ESSQTGTSTTSSR.+2y12.light	41.9
**827.33361**	897.427215	8.67	sp|P23588|IF4B_HUMAN.TGS[Pho]ESSQTGTSTTSSR.+2y9.light	41.9
**827.33361**	796.379536	8.67	sp|P23588|IF4B_HUMAN.TGS[Pho]ESSQTGTSTTSSR.+2y8.light	41.9
**827.33361**	638.310394	8.67	sp|P23588|IF4B_HUMAN.TGS[Pho]ESSQTGTSTTSSR.+2y6.light	41.9
**827.33361**	551.278366	8.67	sp|P23588|IF4B_HUMAN.TGS[Pho]ESSQTGTSTTSSR.+2y5.light	41.9
**832.337745**	1209.558118	8.67	sp|P23588|IF4B_HUMAN.TGS[Pho]ESSQTGTSTTSSR.+2y12.heavy	41.9
**832.337745**	907.435484	8.67	sp|P23588|IF4B_HUMAN.TGS[Pho]ESSQTGTSTTSSR.+2y9.heavy	41.9

**832.337745**	806.387805	8.67	sp|P23588|IF4B_HUMAN.TGS[Pho]ESSQTGTSTTSSR.+2y8.heavy	41.9
**832.337745**	648.318663	8.67	sp|P23588|IF4B_HUMAN.TGS[Pho]ESSQTGTSTTSSR.+2y6.heavy	41.9
**832.337745**	561.286635	8.67	sp|P23588|IF4B_HUMAN.TGS[Pho]ESSQTGTSTTSSR.+2y5.heavy	41.9
**550.2645**	986.437659	37	sp|O96017|CHK2_HUMAN.ILGETS[Pho]LMR.+2y8.light	29.7
**550.2645**	873.353595	37	sp|O96017|CHK2_HUMAN.ILGETS[Pho]LMR.+2y7.light	29.7
**550.2645**	687.289538	37	sp|O96017|CHK2_HUMAN.ILGETS[Pho]LMR.+2y5.light	29.7
**550.2645**	586.24186	37	sp|O96017|CHK2_HUMAN.ILGETS[Pho]LMR.+2y4.light	29.7
**550.2645**	493.722468	37	sp|O96017|CHK2_HUMAN.ILGETS[Pho]LMR.+2y8+2.light	29.7
**555.268634**	996.445928	37	sp|O96017|CHK2_HUMAN.ILGETS[Pho]LMR.+2y8.heavy	29.7
**555.268634**	883.361864	37	sp|O96017|CHK2_HUMAN.ILGETS[Pho]LMR.+2y7.heavy	29.7
**555.268634**	697.297807	37	sp|O96017|CHK2_HUMAN.ILGETS[Pho]LMR.+2y5.heavy	29.7
**555.268634**	596.250129	37	sp|O96017|CHK2_HUMAN.ILGETS[Pho]LMR.+2y4.heavy	29.7
**555.268634**	498.726602	37	sp|O96017|CHK2_HUMAN.ILGETS[Pho]LMR.+2y8+2.heavy	29.7
**568.918555**	834.446828	33.48	sp|P35222|CTNB1_HUMAN.TS[Pho]MGGTQQQFVEGVR.+3y7.light	29.5
**568.918555**	706.38825	33.48	sp|P35222|CTNB1_HUMAN.TS[Pho]MGGTQQQFVEGVR.+3y6.light	29.5
**568.918555**	559.319837	33.48	sp|P35222|CTNB1_HUMAN.TS[Pho]MGGTQQQFVEGVR.+3y5.light	29.5
**568.918555**	460.251423	33.48	sp|P35222|CTNB1_HUMAN.TS[Pho]MGGTQQQFVEGVR.+3y4.light	29.5
**568.918555**	331.208829	33.48	sp|P35222|CTNB1_HUMAN.TS[Pho]MGGTQQQFVEGVR.+3y3.light	29.5
**572.254644**	844.455097	33.48	sp|P35222|CTNB1_HUMAN.TS[Pho]MGGTQQQFVEGVR.+3y7.heavy	29.5
**572.254644**	716.396519	33.48	sp|P35222|CTNB1_HUMAN.TS[Pho]MGGTQQQFVEGVR.+3y6.heavy	29.5
**572.254644**	569.328106	33.48	sp|P35222|CTNB1_HUMAN.TS[Pho]MGGTQQQFVEGVR.+3y5.heavy	29.5
**572.254644**	470.259692	33.48	sp|P35222|CTNB1_HUMAN.TS[Pho]MGGTQQQFVEGVR.+3y4.heavy	29.5
**572.254644**	341.217098	33.48	sp|P35222|CTNB1_HUMAN.TS[Pho]MGGTQQQFVEGVR.+3y3.heavy	29.5
**891.911496**	577.282782	31.52	sp|O60934|NBN_HUMAN.TTTPGPSLS[Pho]QGVSVDEK.+2y5.light	44.7
**891.911496**	391.18234	31.52	sp|O60934|NBN_HUMAN.TTTPGPSLS[Pho]QGVSVDEK.+2y3.light	44.7
**891.911496**	790.863818	31.52	sp|O60934|NBN_HUMAN.TTTPGPSLS[Pho]QGVSVDEK.+2y15+2.light	44.7
**891.911496**	740.339978	31.52	sp|O60934|NBN_HUMAN.TTTPGPSLS[Pho]QGVSVDEK.+2y14+2.light	44.7
**891.911496**	691.813597	31.52	sp|O60934|NBN_HUMAN.TTTPGPSLS[Pho]QGVSVDEK.+2y13+2.light	44.7
**895.918596**	585.296981	31.52	sp|O60934|NBN_HUMAN.TTTPGPSLS[Pho]QGVSVDEK.+2y5.heavy	44.7
**895.918596**	399.196539	31.52	sp|O60934|NBN_HUMAN.TTTPGPSLS[Pho]QGVSVDEK.+2y3.heavy	44.7
**895.918596**	794.870917	31.52	sp|O60934|NBN_HUMAN.TTTPGPSLS[Pho]QGVSVDEK.+2y15+2.heavy	44.7
**895.918596**	744.347078	31.52	sp|O60934|NBN_HUMAN.TTTPGPSLS[Pho]QGVSVDEK.+2y14+2.heavy	44.7
**895.918596**	695.820696	31.52	sp|O60934|NBN_HUMAN.TTTPGPSLS[Pho]QGVSVDEK.+2y13+2.heavy	44.7
**632.96986**	952.413553	29.72	sp|Q8N122|RPTOR_HUMAN.VLDTSSLTQSAPAS[Pho]PTNK.+3y9.light	32.8
**632.96986**	794.344411	29.72	sp|Q8N122|RPTOR_HUMAN.VLDTSSLTQSAPAS[Pho]PTNK.+3y7.light	32.8
**632.96986**	697.291647	29.72	sp|Q8N122|RPTOR_HUMAN.VLDTSSLTQSAPAS[Pho]PTNK.+3y6.light	32.8
**632.96986**	459.256174	29.72	sp|Q8N122|RPTOR_HUMAN.VLDTSSLTQSAPAS[Pho]PTNK.+3y4.light	32.8
**632.96986**	397.675843	29.72	sp|Q8N122|RPTOR_HUMAN.VLDTSSLTQSAPAS[Pho]PTNK.+3y7+2.light	32.8
**635.64126**	960.427752	29.72	sp|Q8N122|RPTOR_HUMAN.VLDTSSLTQSAPAS[Pho]PTNK.+3y9.heavy	32.8
**635.64126**	802.35861	29.72	sp|Q8N122|RPTOR_HUMAN.VLDTSSLTQSAPAS[Pho]PTNK.+3y7.heavy	32.8
**635.64126**	705.305846	29.72	sp|Q8N122|RPTOR_HUMAN.VLDTSSLTQSAPAS[Pho]PTNK.+3y6.heavy	32.8
**635.64126**	467.270373	29.72	sp|Q8N122|RPTOR_HUMAN.VLDTSSLTQSAPAS[Pho]PTNK.+3y4.heavy	32.8
**635.64126**	401.682943	29.72	sp|Q8N122|RPTOR_HUMAN.VLDTSSLTQSAPAS[Pho]PTNK.+3y7+2.heavy	32.8
**659.625304**	794.344411	28.12	sp|Q8N122|RPTOR_HUMAN.VLDTSSLTQS[Pho]APAS[Pho]PTNK.+3y7.light	34.1
**659.625304**	459.256174	28.12	sp|Q8N122|RPTOR_HUMAN.VLDTSSLTQS[Pho]APAS[Pho]PTNK.+3y4.light	34.1
**659.625304**	362.20341	28.12	sp|Q8N122|RPTOR_HUMAN.VLDTSSLTQS[Pho]APAS[Pho]PTNK.+3y3.light	34.1
**659.625304**	397.675843	28.12	sp|Q8N122|RPTOR_HUMAN.VLDTSSLTQS[Pho]APAS[Pho]PTNK.+3y7+2.light	34.1
**659.625304**	349.149461	28.12	sp|Q8N122|RPTOR_HUMAN.VLDTSSLTQS[Pho]APAS[Pho]PTNK.+3y6+2.light	34.1
**662.296704**	802.35861	28.12	sp|Q8N122|RPTOR_HUMAN.VLDTSSLTQS[Pho]APAS[Pho]PTNK.+3y7.heavy	34.1
**662.296704**	467.270373	28.12	sp|Q8N122|RPTOR_HUMAN.VLDTSSLTQS[Pho]APAS[Pho]PTNK.+3y4.heavy	34.1
**662.296704**	370.217609	28.12	sp|Q8N122|RPTOR_HUMAN.VLDTSSLTQS[Pho]APAS[Pho]PTNK.+3y3.heavy	34.1
**662.296704**	401.682943	28.12	sp|Q8N122|RPTOR_HUMAN.VLDTSSLTQS[Pho]APAS[Pho]PTNK.+3y7+2.heavy	34.1
**662.296704**	353.156561	28.12	sp|Q8N122|RPTOR_HUMAN.VLDTSSLTQS[Pho]APAS[Pho]PTNK.+3y6+2.heavy	34.1
**563.250435**	939.393152	15.87	sp|P46527|CDN1B_HUMAN.VSNGS[Pho]PSLER.+2y8.light	30.3
**563.250435**	825.350224	15.87	sp|P46527|CDN1B_HUMAN.VSNGS[Pho]PSLER.+2y7.light	30.3
**563.250435**	601.330401	15.87	sp|P46527|CDN1B_HUMAN.VSNGS[Pho]PSLER.+2y5.light	30.3
**563.250435**	417.245609	15.87	sp|P46527|CDN1B_HUMAN.VSNGS[Pho]PSLER.+2y3.light	30.3

**563.250435**	470.200214	15.87	sp|P46527|CDN1B_HUMAN.VSNGS[Pho]PSLER.+2y8+2.light	30.3
**568.25457**	949.401421	15.87	sp|P46527|CDN1B_HUMAN.VSNGS[Pho]PSLER.+2y8.heavy	30.3
**568.25457**	835.358493	15.87	sp|P46527|CDN1B_HUMAN.VSNGS[Pho]PSLER.+2y7.heavy	30.3
**568.25457**	611.33867	15.87	sp|P46527|CDN1B_HUMAN.VSNGS[Pho]PSLER.+2y5.heavy	30.3
**568.25457**	427.253878	15.87	sp|P46527|CDN1B_HUMAN.VSNGS[Pho]PSLER.+2y3.heavy	30.3
**568.25457**	475.204348	15.87	sp|P46527|CDN1B_HUMAN.VSNGS[Pho]PSLER.+2y8+2.heavy	30.3
**526.262814**	952.449938	20.89	sp|O00443|P3C2A_HUMAN.VSNLQVS[Pho]PK.+2y8.light	28.7
**526.262814**	865.41791	20.89	sp|O00443|P3C2A_HUMAN.VSNLQVS[Pho]PK.+2y7.light	28.7
**526.262814**	751.374983	20.89	sp|O00443|P3C2A_HUMAN.VSNLQVS[Pho]PK.+2y6.light	28.7
**526.262814**	411.163927	20.89	sp|O00443|P3C2A_HUMAN.VSNLQVS[Pho]PK.+2y3.light	28.7
**526.262814**	301.150646	20.89	sp|O00443|P3C2A_HUMAN.VSNLQVS[Pho]PK.+2b3.light	28.7
**530.269914**	960.464137	20.89	sp|O00443|P3C2A_HUMAN.VSNLQVS[Pho]PK.+2y8.heavy	28.7
**530.269914**	873.432109	20.89	sp|O00443|P3C2A_HUMAN.VSNLQVS[Pho]PK.+2y7.heavy	28.7
**530.269914**	759.389182	20.89	sp|O00443|P3C2A_HUMAN.VSNLQVS[Pho]PK.+2y6.heavy	28.7
**530.269914**	419.178126	20.89	sp|O00443|P3C2A_HUMAN.VSNLQVS[Pho]PK.+2y3.heavy	28.7
**530.269914**	301.150646	20.89	sp|O00443|P3C2A_HUMAN.VSNLQVS[Pho]PK.+2b3.heavy	28.7
**633.294311**	966.433226	27.81	sp|P06493|CDK1_HUMAN.IGEGT[Pho]YGVVYK.+2y8.light	33.4
**633.294311**	909.411762	27.81	sp|P06493|CDK1_HUMAN.IGEGT[Pho]YGVVYK.+2y7.light	33.4
**633.294311**	310.176132	27.81	sp|P06493|CDK1_HUMAN.IGEGT[Pho]YGVVYK.+2y2.light	33.4
**633.294311**	364.702514	27.81	sp|P06493|CDK1_HUMAN.IGEGT[Pho]YGVVYK.+2y6+2.light	33.4
**633.294311**	300.155397	27.81	sp|P06493|CDK1_HUMAN.IGEGT[Pho]YGVVYK.+2b3.light	33.4
**637.301411**	974.447425	27.81	sp|P06493|CDK1_HUMAN.IGEGT[Pho]YGVVYK.+2y8.heavy	33.4
**637.301411**	917.425961	27.81	sp|P06493|CDK1_HUMAN.IGEGT[Pho]YGVVYK.+2y7.heavy	33.4
**637.301411**	318.190331	27.81	sp|P06493|CDK1_HUMAN.IGEGT[Pho]YGVVYK.+2y2.heavy	33.4
**637.301411**	368.709614	27.81	sp|P06493|CDK1_HUMAN.IGEGT[Pho]YGVVYK.+2y6+2.heavy	33.4
**637.301411**	300.155397	27.81	sp|P06493|CDK1_HUMAN.IGEGT[Pho]YGVVYK.+2b3.heavy	33.4
**558.223978**	880.415922	22.04	sp|P29803|ODPAT_HUMAN.YHGHS[Pho]MSDPGVSYR.+3y8.light	29
**558.223978**	678.35695	22.04	sp|P29803|ODPAT_HUMAN.YHGHS[Pho]MSDPGVSYR.+3y6.light	29
**558.223978**	581.304186	22.04	sp|P29803|ODPAT_HUMAN.YHGHS[Pho]MSDPGVSYR.+3y5.light	29
**558.223978**	339.682113	22.04	sp|P29803|ODPAT_HUMAN.YHGHS[Pho]MSDPGVSYR.+3y6+2.light	29
**558.223978**	498.157492	22.04	sp|P29803|ODPAT_HUMAN.YHGHS[Pho]MSDPGVSYR.+3b8+2.light	29
**561.560068**	890.424191	22.04	sp|P29803|ODPAT_HUMAN.YHGHS[Pho]MSDPGVSYR.+3y8.heavy	29
**561.560068**	688.365219	22.04	sp|P29803|ODPAT_HUMAN.YHGHS[Pho]MSDPGVSYR.+3y6.heavy	29
**561.560068**	591.312455	22.04	sp|P29803|ODPAT_HUMAN.YHGHS[Pho]MSDPGVSYR.+3y5.heavy	29
**561.560068**	344.686248	22.04	sp|P29803|ODPAT_HUMAN.YHGHS[Pho]MSDPGVSYR.+3y6+2.heavy	29
**561.560068**	498.157492	22.04	sp|P29803|ODPAT_HUMAN.YHGHS[Pho]MSDPGVSYR.+3b8+2.heavy	29
**753.329611**	1024.494566	23.95	sp|O14757|CHK1_HUMAN.VTS[Pho]GGVSESPSGFSK.+2y10.light	38.6
**753.329611**	925.426152	23.95	sp|O14757|CHK1_HUMAN.VTS[Pho]GGVSESPSGFSK.+2y9.light	38.6
**753.329611**	838.394124	23.95	sp|O14757|CHK1_HUMAN.VTS[Pho]GGVSESPSGFSK.+2y8.light	38.6
**753.329611**	709.351531	23.95	sp|O14757|CHK1_HUMAN.VTS[Pho]GGVSESPSGFSK.+2y7.light	38.6
**753.329611**	622.319502	23.95	sp|O14757|CHK1_HUMAN.VTS[Pho]GGVSESPSGFSK.+2y6.light	38.6
**757.33671**	1032.508765	23.95	sp|O14757|CHK1_HUMAN.VTS[Pho]GGVSESPSGFSK.+2y10.heavy	38.6
**757.33671**	933.440351	23.95	sp|O14757|CHK1_HUMAN.VTS[Pho]GGVSESPSGFSK.+2y9.heavy	38.6
**757.33671**	846.408323	23.95	sp|O14757|CHK1_HUMAN.VTS[Pho]GGVSESPSGFSK.+2y8.heavy	38.6
**757.33671**	717.36573	23.95	sp|O14757|CHK1_HUMAN.VTS[Pho]GGVSESPSGFSK.+2y7.heavy	38.6
**757.33671**	630.333701	23.95	sp|O14757|CHK1_HUMAN.VTS[Pho]GGVSESPSGFSK.+2y6.heavy	38.6
**565.729336**	967.388066	12.61	sp|O14757|CHK1_HUMAN.YSSS[Pho]QPEPR.+2y8.light	30.4
**565.729336**	880.356038	12.61	sp|O14757|CHK1_HUMAN.YSSS[Pho]QPEPR.+2y7.light	30.4
**565.729336**	793.32401	12.61	sp|O14757|CHK1_HUMAN.YSSS[Pho]QPEPR.+2y6.light	30.4
**565.729336**	626.32565	12.61	sp|O14757|CHK1_HUMAN.YSSS[Pho]QPEPR.+2y5.light	30.4
**565.729336**	498.267073	12.61	sp|O14757|CHK1_HUMAN.YSSS[Pho]QPEPR.+2y4.light	30.4
**570.73347**	977.396335	12.61	sp|O14757|CHK1_HUMAN.YSSS[Pho]QPEPR.+2y8.heavy	30.4
**570.73347**	890.364307	12.61	sp|O14757|CHK1_HUMAN.YSSS[Pho]QPEPR.+2y7.heavy	30.4
**570.73347**	803.332279	12.61	sp|O14757|CHK1_HUMAN.YSSS[Pho]QPEPR.+2y6.heavy	30.4
**570.73347**	636.333919	12.61	sp|O14757|CHK1_HUMAN.YSSS[Pho]QPEPR.+2y5.heavy	30.4
**570.73347**	508.275342	12.61	sp|O14757|CHK1_HUMAN.YSSS[Pho]QPEPR.+2y4.heavy	30.4
**533.741192**	758.361384	25.6	sp|Q8NCD3|HJURP_HUMAN.LPSS[Pho]PLGC[CAM]R.+2y7 -98.light	29

**533.741192**	602.307892	25.6	sp|Q8NCD3|HJURP_HUMAN.LPSS[Pho]PLGC[CAM]R.+2y5.light	29
**533.741192**	392.171064	25.6	sp|Q8NCD3|HJURP_HUMAN.LPSS[Pho]PLGC[CAM]R.+2y3.light	29
**533.741192**	477.19916	25.6	sp|Q8NCD3|HJURP_HUMAN.LPSS[Pho]PLGC[CAM]R.+2y8+2.light	29
**533.741192**	336.168316	25.6	sp|Q8NCD3|HJURP_HUMAN.LPSS[Pho]PLGC[CAM]R.+2y6 -98+2.light	29
**538.745326**	768.369653	25.6	sp|Q8NCD3|HJURP_HUMAN.LPSS[Pho]PLGC[CAM]R.+2y7 -98.heavy	29
**538.745326**	612.316161	25.6	sp|Q8NCD3|HJURP_HUMAN.LPSS[Pho]PLGC[CAM]R.+2y5.heavy	29
**538.745326**	402.179333	25.6	sp|Q8NCD3|HJURP_HUMAN.LPSS[Pho]PLGC[CAM]R.+2y3.heavy	29
**538.745326**	482.203294	25.6	sp|Q8NCD3|HJURP_HUMAN.LPSS[Pho]PLGC[CAM]R.+2y8+2.heavy	29
**538.745326**	341.17245	25.6	sp|Q8NCD3|HJURP_HUMAN.LPSS[Pho]PLGC[CAM]R.+2y6 -98+2.heavy	29
