# Supplementary material for: An oncogene addiction phosphorylation signature and its derived scores inform tumor responsiveness to targeted therapies
Source: Cell Mol Life Sci. 2022 Dec 10;80(1):6. doi: 10.1007/s00018-022-04634-2 (PMC9734221; doi:10.1007/s00018-022-04634-2)
Supplement: Supplementary file 4 — Supplementary file4 (PDF 58 KB) [file 18_2022_4634_MOESM4_ESM.pdf]

**Cell line**	**EGFR-activating mutation**	**EGFR T790M mutation**	**Constitutive MET activation (pMET)**	**Responsiveness to EGFRi**	**Responsiveness to METi**
**HCC827**	E746-A750 del	No	Yes	Yes	No
**PC-9**	E746-A750 del	No	No	Yes	No
**H1975**	L858R	Yes	No	Yes (AZD9291)	No
**H1993**	No (wt)	No	Yes	No	Yes
**A549**	No (wt)	No	Yes	No	No
**EBC-1**	No (wt)	No	Yes	No	Yes

Supplementary Table 3. Genomic background and treatment responsiveness of cell lines included in the EGFR-positive panel.
